# Improving early prediction for adverse dengue outcomes using data from the IDAMS prospective multicentre study

**DOI:** 10.1038/s41467-026-76450-2

**Published:** 2026-08-21

**Authors:** Phung Khanh Lam, Kerstin Daniela Rosenberger, Dong Thi Hoai Tam, Nguyen Tan Thanh Kieu, Tran Van Ngoc, Nguyen Tran Nam, Do Chau Viet, Nguyen Van Kinh, Sophie Yacoub, Ngoun Chanpheaktra, Varun Kumar, Lucy Lum Chai See, Jameela Sathar, Ernesto Pleités Sandoval, Gabriela Maria Marón, Ida Safitri Laksanawati, Yodi Mahendradhata, Md Zabir Hassan, Firoz Ahmed, Andrea Caprara, Bruno Souza Benevides, Ernesto T. A. Marques, Tereza Magalhaes, Patricia Brasil, Marco Netto, Adriana Tami, Sarah E. Bethencourt, Maria G. Guzman, Cameron Simmons, Nguyen Than Ha Quyen, Marcel Wolbers, Ronald B. Geskus, Thomas Jaenisch, Bridget Wills, on behalf of the International Research Consortium on Dengue Risk Assessment, Management, and Surveillance Clinical Investigators Group, Firoz Ahmed, Md Zabir Hasan, Bruno Souza Benevides, Andrea Caprara, Tereza Magalhaes, Ernesto T. A. Marques, Patricia Brasil, Marco Netto, Ngoun Chanpheaktra, Varun Kumar, Maria G. Guzman, Gabriela Maria Marón, Ernesto Pleités Sandoval, Thomas Jaenisch, Kerstin Daniela Rosenberger, Ida Safitri Laksanawati, Yodi Mahendradhata, Jameela Sathar, Lucy Lum Chai See, Sarah E. Bethencourt, Adriana Tami, Do Chau Viet, Dong Thi Hoai Tam, Ronald B. Geskus, Nguyen Tan Thanh Kieu, Nguyen Than Ha Quyen, Nguyen Tran Nam, Phung Khanh Lam, Cameron Simmons, Tran Van Ngoc, Bridget Wills, Marcel Wolbers, Nguyen Van Kinh, Sophie Yacoub

**Affiliations:** Bangladesh, Dhaka; Brazil, Fortaleza; Brazil, Recife, Pernambuco; Brazil, Rio de Janeiro; Cambodia, Siem Reap; Cuba, Havana; El Salvador, San Salvador; Germany, Heidelberg; Indonesia, Yogyakarta; Malaysia, Kuala Lumpur; Venezuela, Valencia; Viet Nam, Ho Chi Minh City; Viet Nam, Hanoi; 1 Oxford University Clinical Research Unit, Ho Chi Minh City, Viet Nam; 2 Section Clinical Tropical Medicine, Department of Infectious Diseases, Heidelberg University Hospital, Heidelberg, Germany; 3 Hospital for Tropical Diseases, Ho Chi Minh City, Viet Nam; 4 Children’s Hospital Number 2, Ho Chi Minh City, Viet Nam; 5 National Hospital for Tropical Diseases, Hanoi, Viet Nam; 6 Angkor Hospital for Children, Siem Reap, Cambodia; 7 East Tennessee State University Quillen College of Medicine, Johnson City, Tennessee, USA; 8 Faculty of Medicine, University of Malaya, Kuala Lumpur, Malaysia; 9 Universiti Malaya Medical Centre, Kuala Lumpur, Malaysia; 10 Ampang Hospital, Kuala Lumpur, Malaysia; 11 Hospital Nacional de Niños Benjamin Bloom, San Salvador, El Salvador; 12 Center for Tropical Medicine, Faculty of Medicine, Public Health and Nursing, Universitas Gadjah Mada, Yogyakarta, Indonesia; 13 BRAC James P Grant School of Public Health, BRAC University, Dhaka, Bangladesh; 14 Johns Hopkins Bloomberg School of Public Health, Baltimore, USA; 15 International Centre for Diarrhoeal Disease Research, Bangladesh, Dhaka, Bangladesh; 16 Noakhali Science and Technology University, Noakhali, Bangladesh; 17 Universidade Estadual Do Ceará, Fortaleza, Brazil; 18 Centro Universitário Christus-Unichristus, Fortaleza, Brazil; 19 Instituto Aggeu Magalhães, Fundação Oswaldo Cruz, Recife, Pernambuco, Brazil; 20 Graduate School of Public Health, Department of Infectious Diseases and Microbiology, University of Pittsburgh, Pittsburgh, USA; 21 Instituto Nacional de Infectologia Evandro Chagas, Fundação Oswaldo Cruz, Rio de Janeir, Brazil; 22 Secretaria Municipal de Saúde de Resende, Rio de Janeiro, Brazil; 23 Facultad de Ciencias de la Salud, Universidad de Carabobo, Valencia, Venezuela; 24 Department of Medical Microbiology and Infection Prevention, University Medical Center Groningen, University of Groningen, Groningen, The Netherlands; 25 Institute of Tropical Medicine Pedro Kouri, PAHO/WHO Collaborating Center for Dengue and its Control, Havana, Cuba; 26 Centre for Tropical Medicine and Global Health, Nuffield Department of Medicine, University of Oxford, Oxford, United Kingdom; 27 Heidelberg Institute of Global Health, Heidelberg University Hospital, Heidelberg, Germany

## Abstract

Early identification of individuals at risk of complications is important to improve dengue case-management and promote appropriate use of limited resources in high burden settings. In this prospective observational cohort, 7428 febrile outpatients were enrolled across 8 countries in Asia and Latin America and followed daily; 2230 individuals with confirmed dengue were included in the analysis, among whom 304 (14%) progressed to moderate/severe dengue and 38 (2%) to severe dengue during follow-up. We demonstrate evolving relationships between common clinical features, WHO clinical warning signs, and simple haematological parameters, with progression. Key predictors of moderate/severe dengue include low lymphocyte percentage, low total white blood cell count, low platelet count, and persistent vomiting and significant abdominal pain/tenderness. Multivariable models incorporating these variables predict the risk of moderate/severe dengue, initially with modest performance (AUC~0.68), but improving subsequently as new daily data is included into the predictions (AUC~0.73-0.85). We also demonstrate that using these models in combination with clinical decision-making pathways could facilitate earlier admission of high-risk patients without markedly increasing admissions of uncomplicated cases. These results should prove instrumental for updating guidelines on dengue triage and management, potentially providing major public health benefits in resource poor settings.

## Introduction

Dengue is the most common mosquito-borne viral infection in the world. Around 4 billion people from more than 125 countries are at risk^1^, and estimates of 50-96 million symptomatic cases and 10,000-20,000 deaths annually have been suggested by independent groups^2,3^. In 2013 the global economic burden was estimated at 8.9 billion USD, resulting in loss of 1.14 million disability-adjusted life-years^4^. Given this devastating impact, especially on deprived communities, WHO has set a target of zero deaths from dengue by 2030 in the roadmap for neglected tropical diseases 2021-2030^5^. To achieve this goal, improving clinical management and identifying effective therapeutics are considered key pillars in the recent global strategic response plan for dengue and other Aedes-borne arboviruses^6^.

Among symptomatic individuals the illness course is markedly heterogeneous, ranging from a self-limited non-specific viral syndrome to severe and potentially fatal disease^7^. The most notable complication is a poorly characterised vascular leak syndrome that can progress to life-threatening dengue shock syndrome (DSS), while other potential problems include bleeding and organ impairment^8^. Any of the four dengue viral (DENV) serotypes can cause severe disease. Higher viremia is known to be associated with increased clinical severity^9,10^, but since complications typically manifest as plasma viremia resolves, immunological mechanisms are also strongly implicated in pathogenesis. Notably, the risk for severe disease increases markedly with secondary heterotypic infections compared to a first/primary exposure^11^.

Despite substantial efforts to identify effective antiviral and/or immunomodulatory drugs, no specific therapeutics are currently available and case management remains primarily supportive. However, with close observation and prompt but judicious volume resuscitation for DSS cases, most patients recover^12,13^. Although the complex interplay between the four viral serotypes and the human immune response to infection has hampered vaccine development^14^, novel vector control techniques are showing considerable promise in field settings^15,16^. However, recognition that comprehensive global prevention of dengue remains a distant prospect has led to renewed efforts to identify individuals at risk for severe disease^17,18^, aiming to improve case management and facilitate appropriate use of limited resources in high burden settings^19,20^.

The World Health Organization (WHO) 2009 guidelines recommend several warning signs to identify individuals likely to progress^7,12,21^, but current evidence for any specific clinical or laboratory marker is weak and operational definitions vary considerably^22^. As emphasised in a recent review^23^, existing prediction models have mostly been derived from small, single-centre studies of patients already hospitalised, and typically address risk prediction in either paediatric or adult populations^24–27^. Only three models have been developed for use in outpatient settings during the early febrile phase^28–30^, and none can be updated to improve risk prediction as the illness episode evolves. Finally, all the models were considered to have high risk of bias, due variously to inclusion of candidate predictors in the outcome definitions, insufficient numbers of participants with the outcome, and failure to consider over-optimism in model performance^23^.

We undertook a prospective multi-centre observational cohort study of febrile patients with suspected dengue enrolled as outpatients early during the course of illness, within the remit of the International Research Consortium on Dengue Risk Assessment, Management, and Surveillance (IDAMS)^31,32^, aiming to develop more universally applicable clinical prediction models for adverse dengue outcomes (moderate/severe dengue and severe dengue^33^). We also wished to formally assess the prognostic value of WHO’s warning signs on the onset of severity later in the illness course. Serial daily observations were available both for cases managed entirely as outpatients and for those admitted, facilitating exploration of the additional contribution of repeated/sequential assessments to risk prediction.

One concern regarding risk prediction algorithms for dengue relates to the number of potentially unnecessary admissions (i.e., individuals who do not in fact progress to severe disease) that an algorithm may recommend, for each individual who does develop the outcome of interest^34^. For example WHO guidelines suggest that presence of any warning sign merits hospitalisation; however, a retrospective analysis from Singapore indicated that while applying this recommendation would have increased early identification of those who developed complications, it would also have resulted in over 30% more of the uncomplicated dengue cases being admitted^35^. Thus, we also include an assessment of whether integrating our models into clinical decision-making pathways enhances pre-emptive hospitalisation of individuals who subsequently progress and at what cost, and compare the utility of our models to the clinician’s decision alone and to a series of decision rules incorporating varying numbers of warning signs.

## Results

### Study enrolment and data completeness

**Figure 1:**
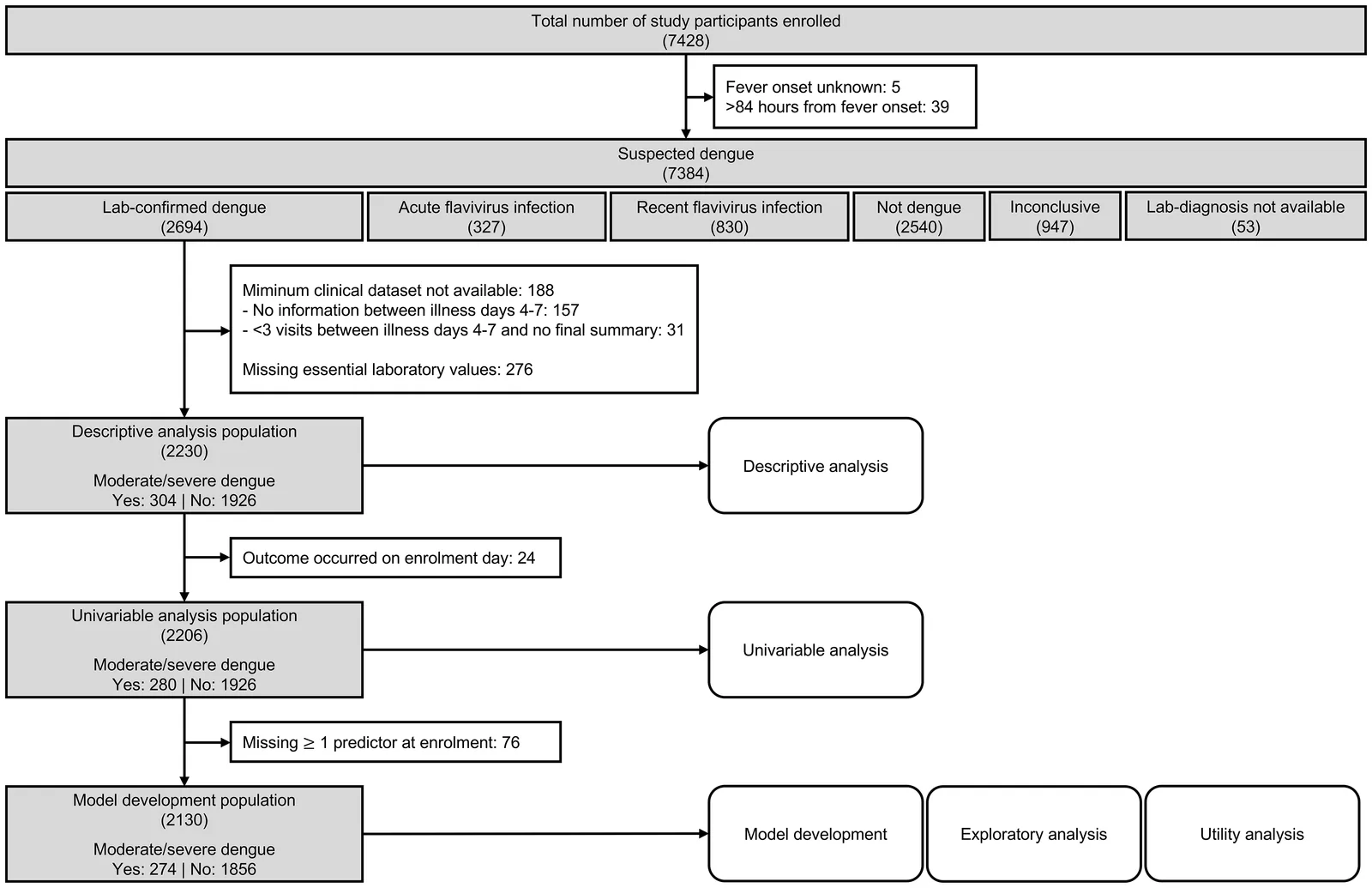
Study Flowchart The moderate/severe dengue outcome could not be defined for 276 participants due to missing information for haemoconcentration and/or liver enzyme levels between study enrolment and illness day 10. The descriptive analysis population included 2230 individuals for whom the primary outcome could be defined; however, only 2206/2230 individuals were included in the univariable analysis, since in 24 patients the final (moderate or severe) outcome occurred on the enrolment day. In this univariable analysis population, 2130/2206 (96%) participants had complete data for all candidate predictors at study enrolment (baseline) and were thus included in development of the prognostic models, the exploratory analysis, and the utility analysis.

A total of 7428 participants were recruited between October 2011 and August 2016, of whom 2694 (37%) had confirmed dengue and were recruited within 3.5 days of fever onset (Supplementary Methods 2-3 and Supplementary Table 1). Within this group, 464/2694 (17%) patients were excluded as relevant outcomes could not be defined while 2230/2694 (83%) individuals had sufficient data to define the main outcomes of interest and are included in our analysis datasets (Figure 1). The individuals included were more likely to enrol slightly later and have probable secondary infections (versus probable primary or inconclusive serostatus) than those excluded from the subsequent analyses (Supplementary Table 8). For most of the selected candidate predictors there was less than 5% missing data at baseline and up to illness day 5. However, missingness increased from illness day 6 onwards, likely reflecting resolution of the illness episode such that some study participants defaulted from the daily follow-up schedule (Supplementary Notes 1 and Supplementary Table 9). Nevertheless, a sensitivity analysis using simple imputation indicates that missing data had little impact on the model assumptions (Supplementary Notes 1 and Supplementary Tables 10 & 11), estimated coefficients (Supplementary Table 12), and model performance (Supplementary Fig. 1).

### Participant characteristics at enrolment and clinical outcomes

**Figure 2:**
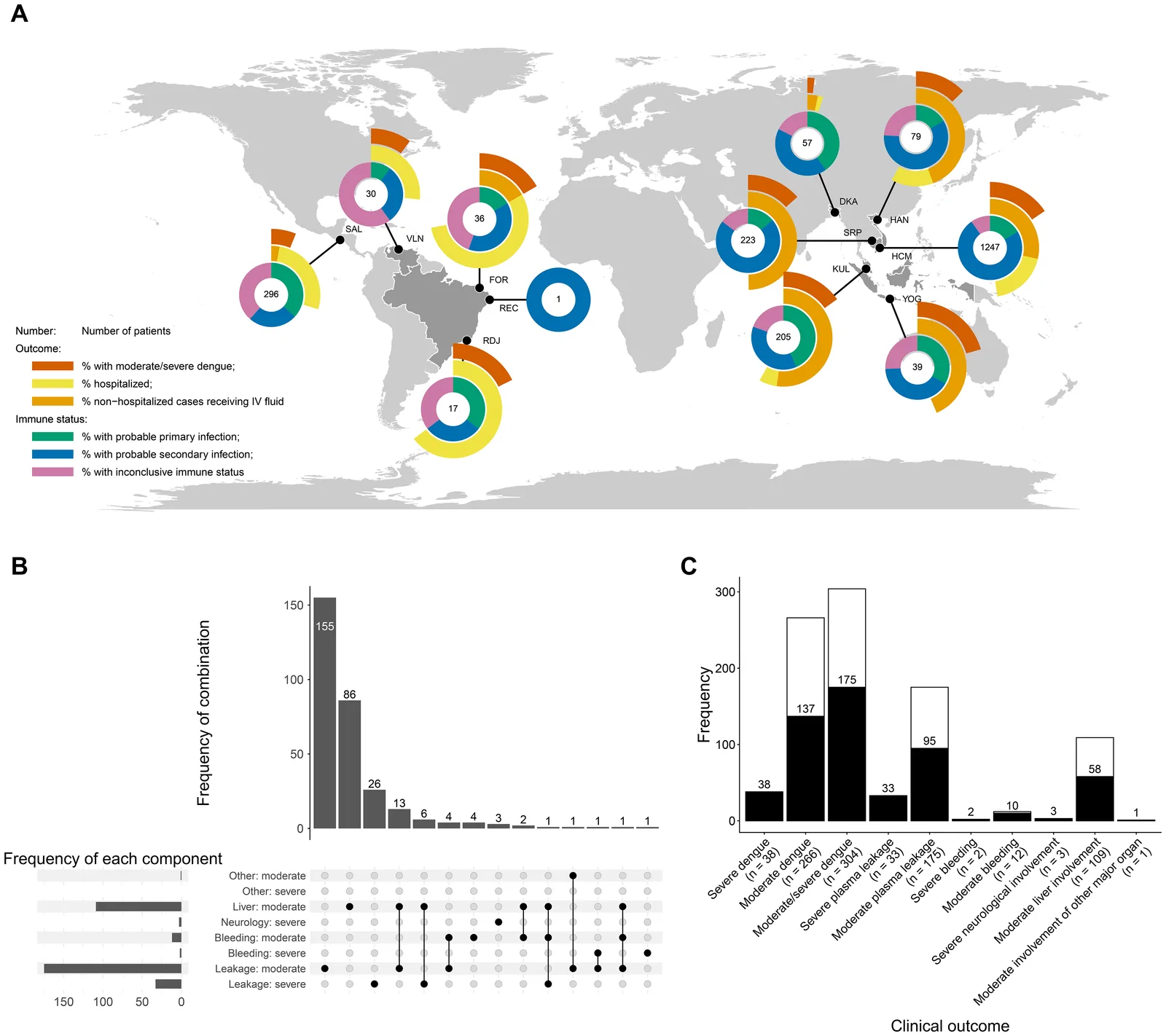
Clinical outcomes of 2230 participants included in the descriptive analysis population. A: Prevalence of outcomes and immune status categories across the study sites for the 2230 participants included in the descriptive analysis population. Study sites are Ho Chi Minh City (HCM), Ha Noi (HAN), Siem Reap (SRP), Dhaka (DKA), Yogyakarta (YOG), Kuala Lumpur (KUL), Fortaleza (FOR), Recife (REC), Rio de Janeiro (RDJ), San Salvador (SAL), Valencia (VLN). B: Components of the clinical outcome observed in the 304 patients with moderate/severe dengue. In this UpSet plot, dots indicate individual clinical entities, while the lines connect the various entities observed in combination. Horizontal bar plots show the frequency of each individual component, and vertical bar plots show the frequency of the various combinations. C: Frequency and percentage of hospitalisation for medical reasons for each type of clinical outcome in the 304 patients with moderate/severe dengue. The bars (full height) show the overall frequency of occurrence of each type of clinical outcome, while the black sections, with the respective number and percentage indicated above each one, indicate the frequency of hospitalisation for medical reasons for each type of clinical outcome. Note: This figure includes the 24 patients in whom the outcome occurred on the day of study enrolment. Source data are provided in the Source Data file. Basemap data from Natural Earth (https://www.naturalearthdata.com).

Demographic characteristics and clinical outcomes for the 2230 patients are described by continent in Table 1. In Latin America (LA) mostly children (age <15 years) were enrolled (85%), while in Asia only 45% of the participants were children, reflecting differences in the populations served at the relevant study sites. Most participants enrolled on illness days 2 or 3, though slightly later in LA compared to Asia; consistent with this finding, the proportion of patients with enrolment viremia under the detection limit was also higher in LA. While DENV-1 and DENV-4 were dominant in Asia, DENV-3 was more prevalent in LA. In Asia, most patients had probable secondary infections. In LA the proportion of probable secondary infections was only 27% but immune status was inconclusive in 40% (Table 1, Figure 2-Panel A).

Considering the main clinical outcome of interest, 304 study participants progressed to moderate/severe dengue during the acute illness. The most frequent specific outcomes observed were moderate plasma leakage (175/304 (58%)) and moderate liver involvement (109/304 (36%)) (Figure 2-Panel B). All 38 patients with severe dengue were hospitalised, together with 137/266 (52%) of those with a moderate final outcome (Figure 2-Panel C), and 468/1926 (24%) patients without specific complications. Among the patients with severe dengue, 13 of 38 developed moderate disease 1-2 days before progressing to severe dengue, another 13 progressed to severe dengue on the same day, while the remaining 12 patients progressed directly to severe dengue without an identifiable moderate stage. There were notable differences in prevalence of the moderate/severe outcome between study sites (Figure 2-Panel A, Supplementary Notes 2 and Supplementary Table 13). However, although the heterogeneity assessment also identified several differences in participant enrolment characteristics between study sites, effects on risk prediction, particularly discriminant ability, were limited (Supplementary Notes 2 and Supplementary Tables 13 & 14).

### Relationships between symptoms, clinical signs, and laboratory tests, with adverse outcomes

In addition to general predictors (age, sex, continent, past medical history, illness day at enrolment), we pre-defined a list of 24 symptoms, clinical signs, and laboratory tests as candidate predictors (Supplementary Methods 7 and Supplementary Table 3) and explored relationships between these factors, assessed at study enrolment and also daily throughout the illness course, with disease progression. For the laboratory tests we focused on haematological variables since these were assessed daily at all sites, while biochemistry tests were performed intermittently and according to local capacity at each site. Table 2 describes the univariable and multivariable associations between these candidate predictors and development of moderate/severe dengue at least one day later. Strong predictors include low lymphocyte percentage (OR [95%CI] per 10% increase: 0.64 [0.54-0.75]), and low total white blood cell (WBC) count (OR [95%CI] per 2-fold increase if below 2,000 cells/mm3: 0.07 [0.01-0.38]). Lower platelet counts were also associated with higher risk of moderate/severe dengue, with a strong association for values <150,000cells/mm^3^ (OR [95%CI] per 10,000 cells/mm3 of 1.16 [1.09-1.23] versus 1.02 [0.98-1.06] for counts ≥150,000cells/mm3). Among other WHO warning signs, persistent vomiting and significant abdominal pain/tenderness were associated with higher risks for moderate/severe dengue (OR [95%CI] of 1.50 [1.12-2.01] and 1.57 [0.93-2.63], respectively), while mucosal bleeding was associated with a lower risk but with wide confidence intervals (OR [95%CI] of 0.83 [0.41-1.70]).

**Figure 3:**
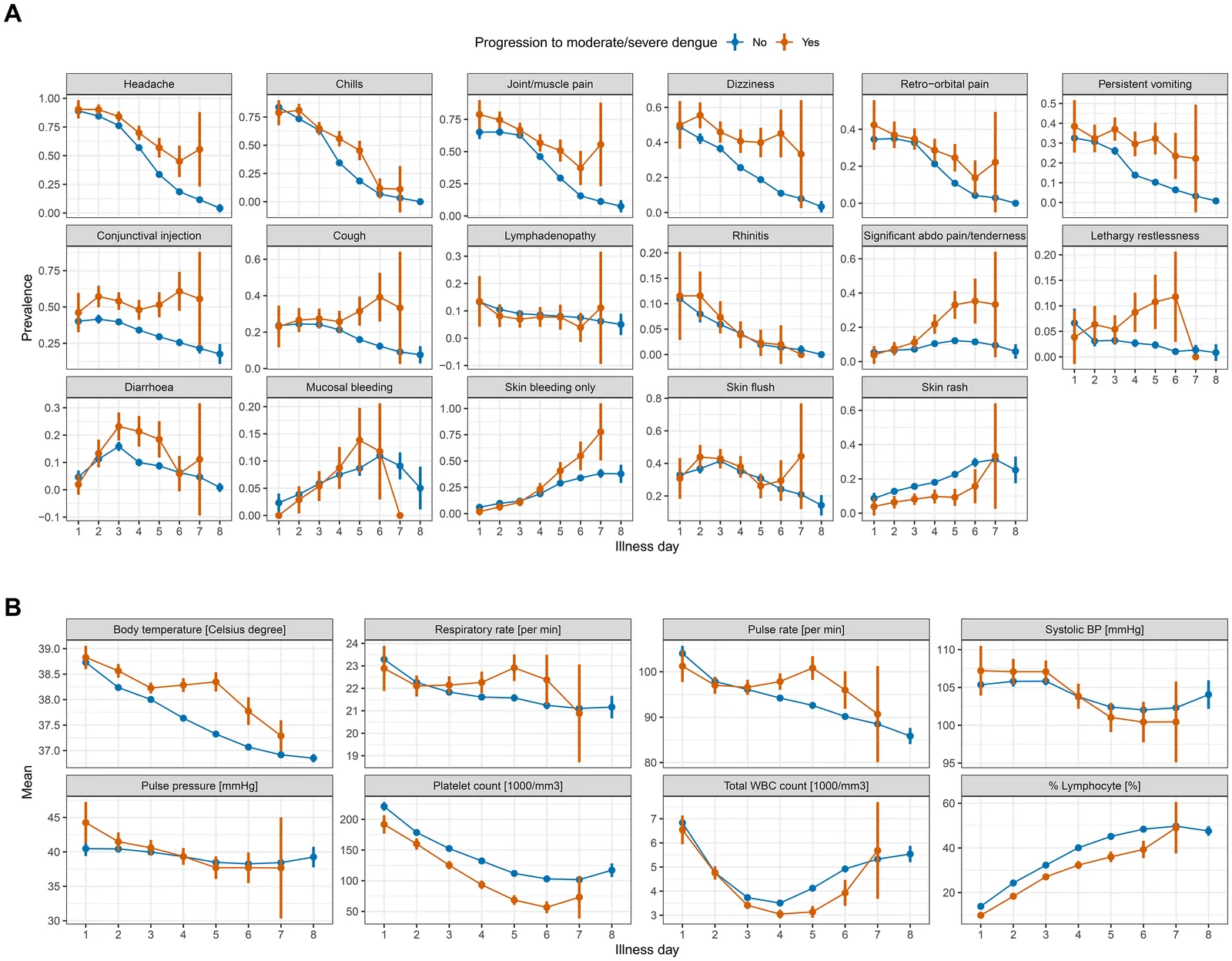
Prevalence of categorical predictors (Panel A) and mean values for continuous predictors (Panel B) by day of illness, for patients who did (in red) and did not (in blue) progress to moderate/severe dengue (n = 2206). Vertical error bars represent the 95% confidence intervals of the point estimate on each day of illness. Only patients with no evidence of moderate/severe dengue up to the day of interest are included (i.e., the 24 cases who developed moderate/severe dengue on the day of enrolment are not represented here). In panel B, data for body temperature, respiratory rate, pulse rate, systolic blood pressure, platelet count, total white cell count (WBC), and % lymphocytes represent the single daily recorded values for outpatients and the worst of the daily recorded values for inpatients (i.e., the highest body temperature, highest respiratory rate, highest systolic blood pressure, lowest platelet count, lowest total WBC and lowest % lymphocytes from the most abnormal full blood count result on that day). Source data for this figure are provided in the Source Data file.

During the first 3 days of illness no obvious differences in the profiles of most signs and symptoms were apparent between study participants who did or did not progress to moderate/severe dengue (Figure 3). However, dizziness and conjunctival injection appeared slightly more common in those who did progress, while skin rash was observed less frequently in this group. On later illness days these differences became more pronounced. Additionally, headache, joint/muscle pain, cough, abdominal pain/tenderness, lethargy/restlessness, and skin bleeding became more prominent in the moderate/severe group. Body temperature was generally higher and remained elevated for longer, and respiratory rate and pulse rate displayed profiles consistent with greater stress during the critical period (illness days 4-7), in the moderate/severe compared to the uncomplicated cases. Systolic blood pressure was also higher during the early febrile period and lower during the critical period in the moderate/severe cases. Lastly the platelet count and percentage lymphocyte count were consistently lower in the moderate/severe than the uncomplicated cases, with greater separation becoming apparent between the groups during the critical period.

### Improving early prediction of adverse clinical outcomes using multivariable prediction models

**Figure 4:**
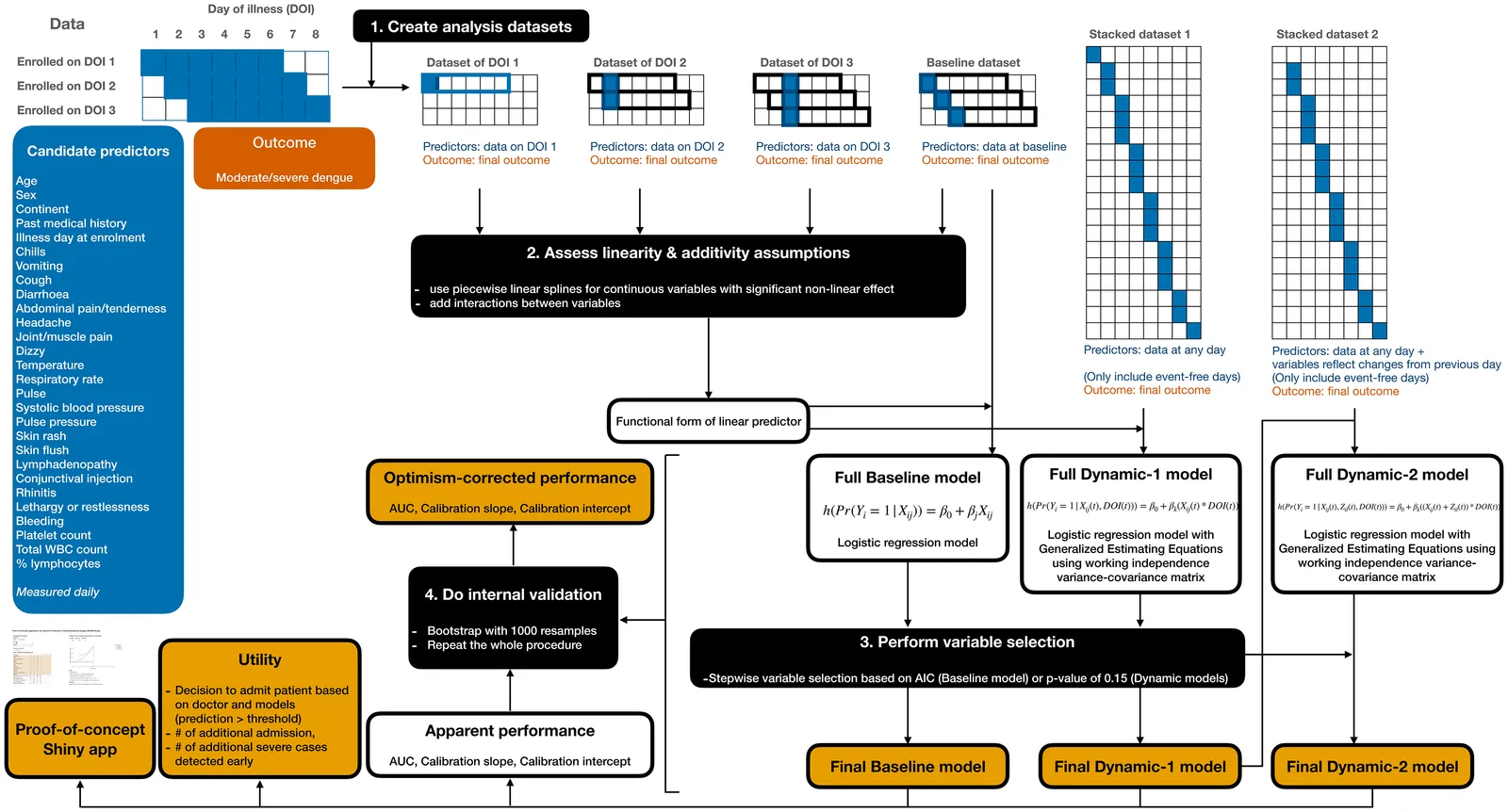
Summary of the model development procedure The prediction models were developed using 4 main steps: (1) Data of the selected candidate predictors (assessed daily), and outcomes, were arranged into a) separate datasets by day of illness (DOI) for the model assumption assessments, b) a combined baseline dataset incorporating all data obtained at enrolment for development of the Baseline model, and c) a stacked dataset that combined the DOI-specific datasets for development of the Dynamic models. (2) Linearity and additivity assumptions were assessed to decide on the functional form of continuous predictors and to include interaction terms where necessary. (3) Variable selection process was performed to choose a subset of candidate predictors for the final models. The variable selection for the Dynamic-2 model relied on the selection of variables chosen for the final Dynamic-1 model. (4) Model performance was assessed using internal validation with 1,000 bootstrapped samples and was corrected for overfitting. Note: AUC = Area Under the ROC Curve, AIC = Akaike Information Criterion.

We developed a Baseline Model using information collected at presentation only (Study Day 1), to predict development of moderate/severe dengue at least one day later (see detailed description in the methods section and Figure 4). We also developed two dynamic prediction models to update prediction over the course of the illness episode; the first (Dynamic-1) used information collected on a specific illness day to predict occurrence of the outcome on any later day, while the second (Dynamic-2) also incorporated changes in certain variables from the previous day alongside absolute values for the specific illness day.

**Figure 5:**
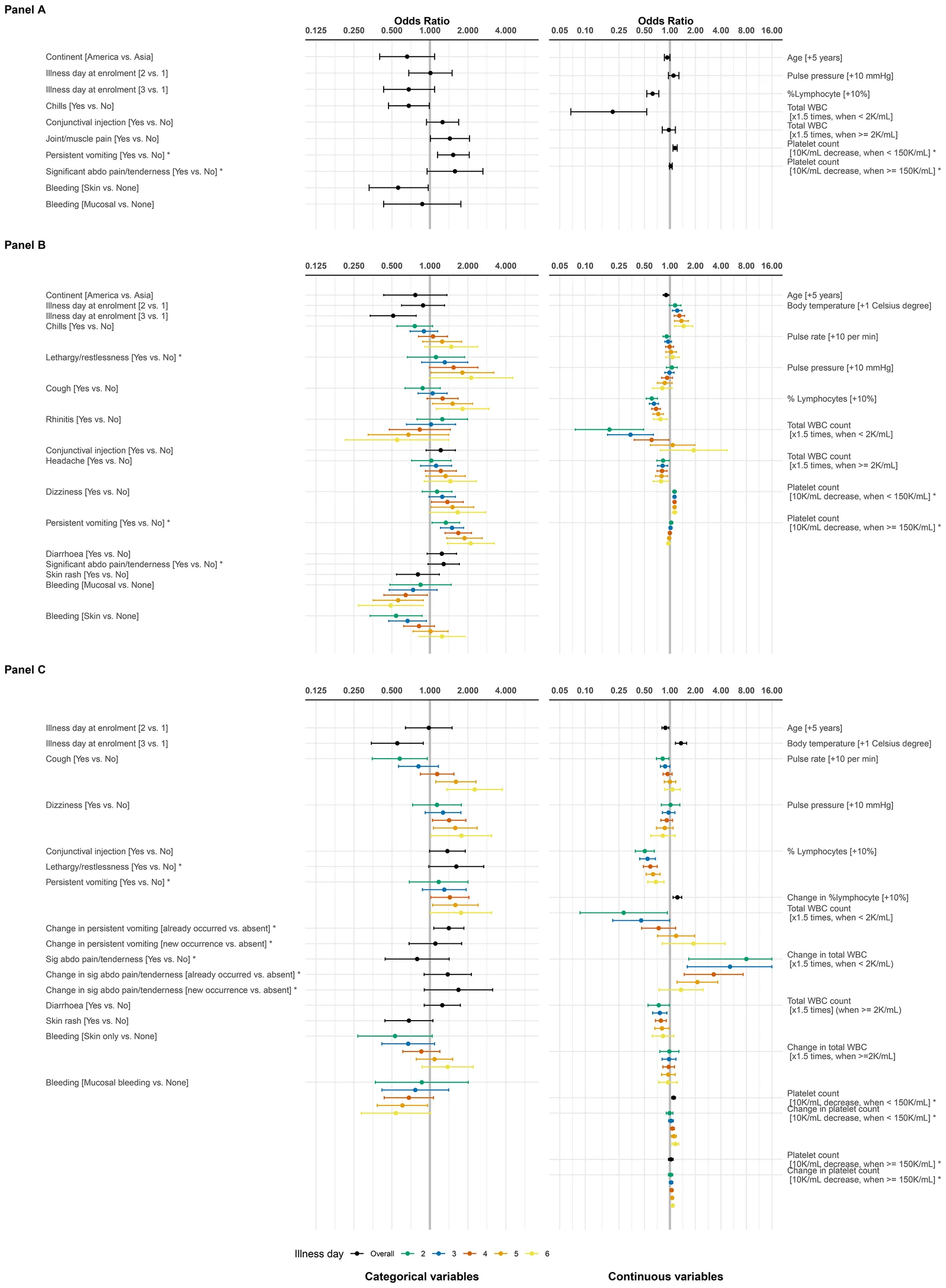
Association of candidate predictors with moderate/severe dengue in the final baseline model (Panel A), Dynamic-1 model (Panel B), and Dynamic-2 model (Panel C) (without interactions) (developed on the model development population, n = 2130). Legend: Points and corresponding horizontal error bars are odds ratios and their 95% confidence intervals for each predictor included in the developed models. Predictors with a fixed association with outcome (labelled Overall) are displayed in black. Predictors with time-varying associations with outcome are displayed in separate horizontal error bars for each illness day; however, illness day 1 is not included due to the high uncertainty arising from the limited data available for that day. WHO warning signs are shown with asterisk symbol. In Panel C, note that the 95% confidence interval on illness days 2 and 3 for the change in total white cell count (WBC), when <2,000/mL, are truncated. The true values are (1.67, 38.47) and (1.59, 16.4) respectively. Source data for this figure are provided in the Source Data file.

**Figure 6:**
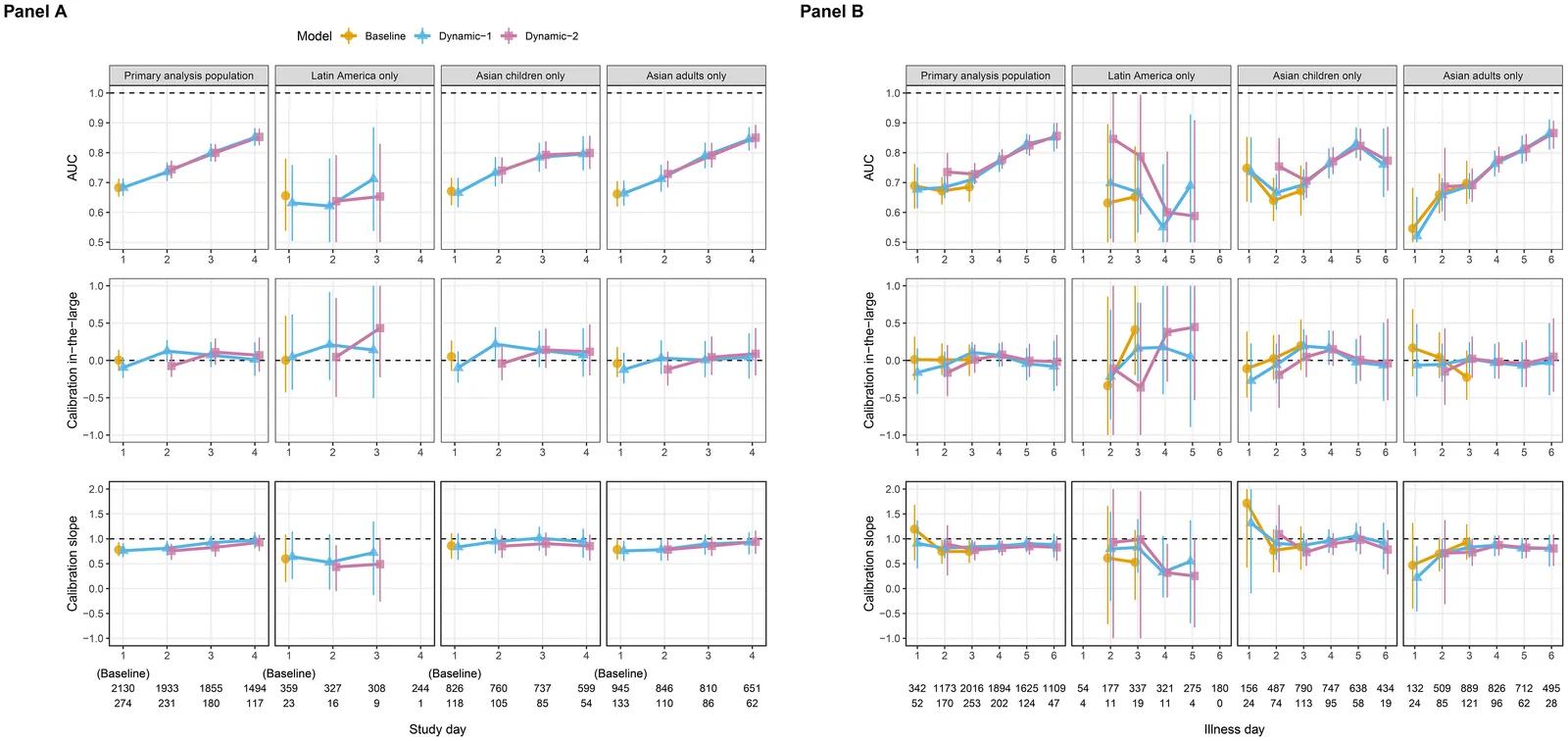
Model performance in predicting moderate/severe dengue (n = 2130). Optimism-corrected AUC (top row), calibration in-the-large (middle row) and calibration slope (bottom row) for the three models, evaluated at baseline and up to 3 days after study enrolment across study subgroups (Panel A) and by illness day (Panel B). Points denote medians, and vertical lines the 2.5th–97.5th percentiles, from 1,000 bootstrap replicates used to estimate optimism. Numbers below each plot indicate the number of participants used for model development on that day (top line) and the number who subsequently developed moderate/severe dengue (bottom line). The Dynamic-2 model could not be evaluated at baseline because it includes day-to-day change predictors. For visual clarity, values were truncated (AUC 0.5–1.0; calibration-in-the-large −1 to 1; calibration slope −1 to 2). Note: Area Under the ROC Curve (AUC) quantifies discrimination – i.e., the probability that a case has higher predicted risk than a non-case (ideal = 1). Calibration reflects agreement between model predictions and the observed outcomes; calibration-in-the-large assesses systematic over/underprediction (ideal = 0) and calibration slope reflects the average effect of the predictors on the outcome (ideal = 1). Source data for this figure are provided in the Source Data file.

The final Baseline Model incorporates 13 clinical and laboratory variables (Figure 5-Panel A). The Dynamic-1 Model additionally includes lethargy, cough, rhinitis, headache, dizziness, diarrhoea, skin rash, temperature and pulse, but excludes joint/muscle pain, with many of these associations demonstrating progressively stronger relationships over time from symptom onset (Figure 5-Panel B). Among the WHO warning signs, the association between significant abdominal pain/tenderness with the moderate/severe outcome was stable, while associations between persistent vomiting and lethargy/restlessness with the outcome increased over time. The association between platelet count above 150K with the outcome reversed over time, but was not strong. Similar relationships were observed using the Dynamic-2 Model (Figure 5-Panel C). The three models can be explored in more detail using our proof-of-concept web application (Supplementary Fig. 5), with the relevant regression coefficients presented in Table 3. We used study day as the primary timescale to assess model performance (Figure 6-Panel A), but also performed similar analyses based on illness day (Figure 6-Panel B), since this is more relevant to how the models might be used in clinical practice.

The predictive value of our models was comparable between children and adults in Asia but was lower among LA participants (Figure 6-Panel A). The Baseline and Dynamic-1 Models that included all participants performed similarly at enrolment (Study Day 1), with an Area Under the Curve (AUC) of 0.68, calibration in-the-large close to 0 and calibration slope <1. On subsequent study days the performance of the Dynamic-1 and Dynamic-2 Models improved (AUCs 0.73-0.85, calibration in-the-large close to 0 and calibration slope close to 1), with little difference between the models. In general model performance in Latin America showed more variability, especially for the dynamic models. Overall, model performance assessed by illness day was consistent with performance assessed by study day. A demonstration of the web application for the three models developed can be accessed at this link: https://lampk.shinyapps.io/IDAMSPRED/ (Supplementary Fig. 5).

### Additional exploratory models

1. Given the known influence of the infecting viral serotype and the individual’s immune status prior to infection on disease severity^9–11^, we also explored the impact of adding this information to our models. However, adding immune status and/or serotype information did not improve the discriminant ability of the models (Supplementary Fig. 2-Panel A). This result was unexpected and may indicate that any influence is mediated via clinical covariates or predictors already incorporated into the models.
2. We also assessed performance of the dynamic models in the (likely more heterogeneous) suspected-dengue population compared to the confirmed-dengue population, following the participant selection flowchart described in Supplementary Fig. 3. Summary descriptions of the relevant patient groups are presented in Supplementary Tables 15-18. We anticipated that the evolving clinical trajectories of non-dengue versus dengue cases towards moderate/severe disease would diverge, making outcome prediction easier in the suspected-dengue population. However, although we observed this among the Asian patient groups, this was not apparent among the LA participants (Supplementary Fig. 4); further exploration (Supplementary Tables 15-18) suggests that the characteristics of the suspected-dengue and confirmed-dengue cases were more similar in LA compared to Asia. Possible reasons include slightly later study enrolment in LA reducing the likelihood of achieving a confirmed-dengue versus an acute/recent flavivirus diagnosis, and differences in the background landscape of concurrent infectious diseases between the continents^31^; most notably transmission of ZIKA virus, a closely related flavivirus, became widespread in LA but not in Southeast Asia during the study enrolment period^36^.
3. Finally, we compared performance of our models with simpler models that rely on platelet count and/or WHO warning signs, in particular a model for severe dengue previously developed by our group for use in outpatient settings^29^. For this we fitted additional models that included only platelet count or warning signs, with and without illness day at enrolment and the interaction between age and continent. However, both of our dynamic models are clearly superior to platelet-only models and also to models that include only warning signs, in terms of discriminant ability (Supplementary Fig. 2-Panel B).

### Assessing the utility of the models to improve decision-making around hospitalisation

**Figure 7:**
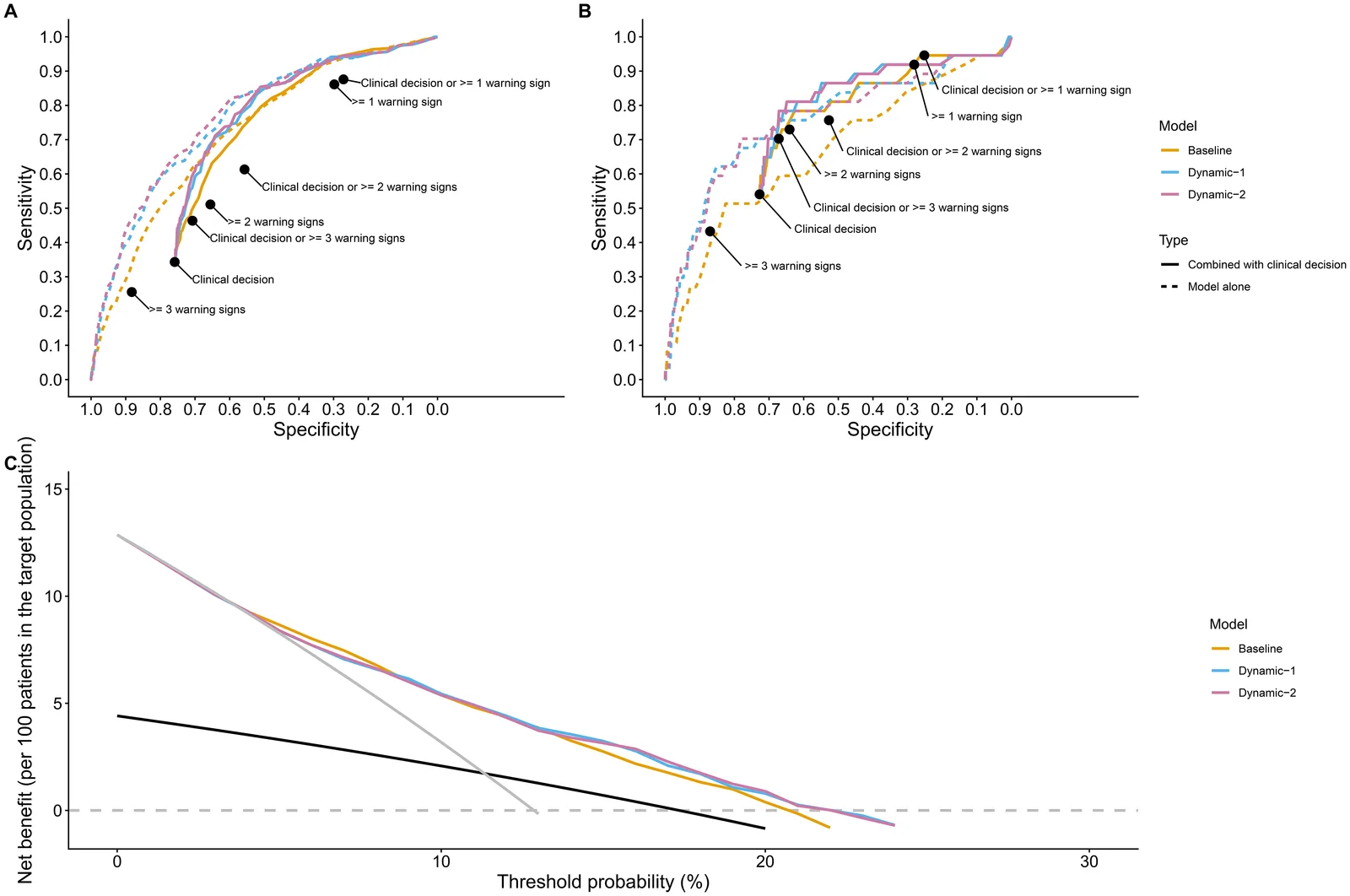
Early prediction of severity using various candidate decision rules compared to hospital admission by clinician’s decision (n = 2130). Panels A and B: Sensitivity and specificity for hospitalising patients at least one day prior to outcome onset (A: moderate/severe dengue, B: severe dengue) using different candidate models and decision rules. In addition to our models, we assessed rule-based strategies based on the number of warning signs (at least 1, 2, or 3 warning signs present up to the day of assessment, including persistent vomiting, significant abdominal pain/tenderness, lethargy/restlessness, mucosal bleeding, or a platelet count <50K/mm3), as well as clinician-initiated admission for any medical reason. Models and rules were assessed both alone and in combination with the clinician’s decision. The three models (Baseline, Dynamic-1, and Dynamic-2) were evaluated at various risk thresholds (i.e., cut-offs at which a clinician might consider the predicted risk sufficient to warrant admission) and are presented as solid lines for model-only strategies and dashed lines for strategies combining the clinician’s decision with one of the three models. Sensitivities and specificities were calculated over the course of the illness for all participants. Panel C: Decision curve analysis evaluating strategies to hospitalise dengue patients. For any given threshold probability, the clinical utility of different strategies to hospitalise patients can be measured in terms of net benefit – i.e., the proportion of pre-emptively hospitalised patients who progress to moderate/severe dengue, minus a weighted penalty for apparently unnecessary admissions. The threshold probability reflects the ratio between the cost of admitting a patient who does not develop the moderate/severe outcome and the cost of missing a patient who does (e.g., a 10% threshold equals a 1:9 ratio). The figure compares six hospitalisation strategies: hospitalise no-one (grey dashed), hospitalise all (grey), clinician’s decision alone (black), and clinician’s decision combined with Baseline (orange), Dynamic-1 (blue), or Dynamic-2 (purple) models. Clinician assessment alone outperforms the hospitalise no-one and hospitalise all strategies at threshold probabilities of 12%–17%. However, combining clinician decisions with any of the prediction models yields the greatest net benefit for threshold probabilities between 1%–22%. Source data for this figure are provided in the Source Data file.

Predicting likely progression early in the dengue disease course is challenging. Timely hospitalisation of an at-risk individual is a key transition point that should facilitate effective case management and ensure a good outcome. However, a high volume of contemporaneous precautionary admissions may compromise care for those most in need. To assess the potential utility of our models to improve decision-making around hospitalisation, we quantified their ability to correctly identify patients with adverse dengue outcomes at least one day before the outcome occurred. First, we accepted the de facto clinicians’ decisions to hospitalise any participant at any timepoint. Then we applied a series of different hospitalisation rules on all remaining participants (i.e., those not admitted at that time), and assessed overall performance in terms of sensitivity (i.e., the proportion of patients who would be admitted prior to outcome development among those with adverse outcomes) and specificity (i.e., the proportion of patients who would not be admitted among those without adverse outcomes) (Figure 7-Panels A & B). For the models, the final decision to hospitalise varies according to a risk threshold at which the responsible clinician considers the predicted risk sufficient to warrant admission. Therefore, we explored a) our three models applied across the range of possible risk-thresholds, and b) decision-rules that relied on the number of warning signs present up to the day of assessment, in each case considering the various models/decision-rules alone, and also when combined with clinicians’ decisions, taking into account the patient’s actual hospitalisation status.

In addition we carried out decision curve analysis^37^ across a range of threshold probabilities to assess the net benefit of applying our models. We explored the net benefit of six different strategies, including hospitalise no-one, hospitalise all, hospitalisation based on the clinicians’ decision alone, and hospitalisation based on different combinations of the clinicians’ decision with our developed models (Figure 7-Panel C).

For both moderate/severe and severe dengue, pre-emptive hospitalisation based only on the clinician’s de facto decision showed high specificity (75% – 80%) but low sensitivity (30% - 50%). Hospitalisation using candidate decision rules incorporating varying numbers of warning signs exhibited a wide spectrum of sensitivity (~80%-90% for at least 1 warning sign, to ~25%-45% for at least 3 warning signs) and specificity (~85%-90% for at least 3 warning signs, to ~ 30% for at least 1 warning sign). Combining the clinician’s decision with the various warning sign decision rules increased sensitivity but reduced specificity. By contrast, decision rules based on our models, both stand alone and when used alongside the clinician’s decision, achieved higher combinations of sensitivity and specificity, with greater improvement seen for the combined moderate/severe outcome than the severe outcome alone. For each assessment the Dynamic Models exhibited higher combined sensitivity and specificity than the Baseline Model.

It is also apparent that the magnitude of improvement varied considerably by the selected risk threshold. From Figure 7-Panel C we see that hospitalisation strategies that rely on clinician-initiated decisions, either alone or in combination with our models, would provide benefit at threshold probabilities <25% (i.e., if the cost ratio of admitting patients who do not progress to moderate/severe dengue versus missing patients who do progress is less than 1:3). However, all combination approaches provide greater benefit than clinician-initiated decisions alone, especially at threshold probabilities < 12% (i.e., for cost ratios less than 1:6.5) when the clinician’s decision alone strategy may provide less benefit than the hospitalise all strategy. Despite negligible differences in net benefit between combination strategies, when the risk threshold is >30% (Supplementary Fig. 6) the combined approach using the Dynamic Models results in fewer uncomplicated or non-severe dengue admissions for each patient who does subsequently develop the relevant outcome (moderate/severe or severe dengue, respectively), as compared to the Baseline Model combined approach.

Of note, the distribution of predicted risks on the day when patients were actually hospitalised (Supplementary Fig. 7), demonstrates substantial differences between study sites, even within the same country. Assuming that predicted risks are the best estimates of the true risk of moderate/severe dengue, these results highlight the considerable variation that exists between clinicians in their thresholds for hospitalisation. This also illustrates the complex nature of, and possible interactions between, severe disease progression and any interventions undertaken as the clinical episode unfolds. Thus, although hospitalisation can be treated as an outcome in itself, closer clinical monitoring and any associated interventions that result, may prevent complications that would otherwise occur.

## Discussion

The IDAMS dataset represents a detailed prospective assessment of the clinical spectrum of symptomatic dengue. More than 2200 adults and children from a range of dengue-endemic settings were closely observed from early in the febrile phase using the same study protocol, thereby facilitating comprehensive evaluation of factors associated with progression to moderate/severe dengue. In addition to a conventional baseline prediction model, we developed novel dynamic models that incorporate sequential individual-patient assessments. Performance of these models for the moderate/severe dengue outcome was modest at baseline, consistent with the recognised diversity of clinical dengue, but improved over time during the acute phase of illness. We also demonstrate that integrating our models into clinical decision-making pathways could enhance pre-emptive hospitalisation of individuals who subsequently progress.

To facilitate widespread application of any models developed we elected to include only clinical signs and symptoms (focusing on WHO’s designated warning signs) and readily accessible laboratory variables. The data highlight the markedly heterogeneous nature of dengue, with age-group and geographical location particularly associated with differences in clinical profiles and outcomes. Nevertheless, between the moderate/severe and uncomplicated groups, diverging trajectories were apparent in various clinical and laboratory variables. Given the heterogeneity we observed, the final models include a fairly large number of variables – 13 for the Baseline Model and 21 each for the Dynamic Models. There was some variation in the variables included in the different models, but the associations identified between warning signs and the moderate/severe outcome were generally stable across all three models, geographical locations and age-groups, providing evidence-based support for their inclusion in WHO guidelines^7^. Persistent vomiting, significant abdominal pain/tenderness and platelet values were invariably selected in our models, consistent with findings from previous prospective studies^29,38,39^. Secondary infection was identified as a risk factor for moderate/severe dengue in univariable analysis, and the lack of improvement in model performance following inclusion of immune status merits careful consideration; one plausible explanation is that the influence of immune status is mediated via clinical covariates or predictors already incorporated into the models.

The use of dynamic models that incorporate sequential daily information is especially relevant for dengue given the rapid clinical evolution. In an earlier study we demonstrated the utility of repeated platelet counts to update prediction for DSS^39^. In the current analysis we have incorporated a range of candidate predictors simultaneously (clinical as well as laboratory variables) and have shown clear superiority with respect to a repeated-values platelet-only model in terms of discriminant ability. Not surprisingly, as the prediction horizon decreased (i.e., as the outcome of interest approached) the performance of both dynamic models increased. However, the added complexity of the Dynamic-2 Model (including change from the previous day’s value as well as today’s new value) did not translate into superior performance of the Dynamic-2 versus the Dynamic-1 model. Notably also, the Dynamic-2 model cannot be used when patients first present to healthcare services as information from the previous day is required.

Decision rules using our prediction models within certain risk threshold limits, and also simple rules that rely only on warning signs, have moderate specificity similar to the clinicians’ de facto decisions, though with higher sensitivity to identify patients with moderate/severe dengue in advance. The moderate sensitivities in the range of 0.5-0.6 are partly explained by the fact that the pooled category of moderate/severe disease contains a wide variety of moderate disease. In effect the moderate disease category needs to be defined more stringently, but this was beyond the aims of this study^33^. These moderate sensitivities and specificities may also reflect the fact that hospital admission likely prevented evolution to more severe disease to some extent, thus diluting the endpoints.

Several limitations to this work merit consideration. Having initially intended to use severe dengue as our primary outcome it became apparent that the rarity of this outcome in an early-outpatient confirmed-dengue population (38/2230, 1.7%) precluded this option. We had originally estimated the progression rate at 3-5%, supported by expert opinion and a study in Vietnamese children hospitalised during the early febrile phase (4%)^40^. Later however, the Sanofi-Pasteur vaccine trial data identified only 2 DSS cases among their 389 confirmed-dengue episodes^41^. Recognising that we could not feasibly conduct a robust analysis involving many candidate predictors, we elected to use instead the combined endpoint of moderate/severe dengue. However, as most patients with moderate/severe dengue were included in the model development at the time when they developed moderate dengue (250/274, 91%), our findings may be more applicable to this outcome rather than to severe dengue. Future research could potentially be conducted on pooled cohort data from this study and other observational cohorts (eg., control arms of vaccine trials) in order to repeat the analysis with a larger sample size and severe dengue as the outcome.

Although severe dengue remains the most relevant clinical outcome in terms of mortality, morbidity and resource utilisation, we have shown that these prediction models can help to detect both severe and moderate cases earlier, though inevitably with some cost in terms of apparently unnecessary admissions. Further work is needed to explore what trade-off in terms of additional admissions might be acceptable to public health authorities (i.e., where to set the prediction threshold), whether this threshold differs in endemic versus non-endemic settings, and the implications in terms of health economics. The expectation is that prediction models invariably enhance clinicians’ prognostic capabilities, but prediction is only one component of the decision-making process^42^, and formal assessment is crucial to determine the true benefit of novel tools in real-world settings^43,44^. Of interest in this regard, a number of experimental biomarkers have recently been evaluated for their ability to predict dengue disease progression, in part using data and biological material from this study^19,45,46^. Although wide-scale implementation of such biomarkers remains unrealistic at present, it could be informative to explore the predictive power of different candidate combinations against our models, or potentially to consider integrating our models alongside promising candidates.

We need also to consider whether heterogeneities resulting from the multicentre nature of the study could affect the performance and generalisability of the models. Although the effect of the selected predictors on the moderate/severe outcome remained pretty robust across geographical locations and age-groups, local validation using an independent dataset and/or re-calibration of the various models should be considered^47^. When miscalibration is evident using local data, the various models can be updated by adjusting the regression coefficients^48^. Additionally, the main analysis was performed on patients with confirmed-dengue, potentially limiting applicability to this group alone. Supplementary analyses including the whole suspected-dengue population supports use of these models in Asia but the performance in LA suggests that, in the absence of appropriate rapid diagnostics, further refinements may be needed before deployment. In addition, since prediction models cannot be interpreted and applied outside the context in which they are developed (in this case the current state of dengue diagnosis and case management), our models will require updating and refinement should major changes to case management be widely adopted, or novel and effective intervention strategies become available.

Another caveat of these analyses is reliance on the assumption that hospitalisation does not change the illness course. However, closer clinical monitoring and any associated interventions after admission could have prevented disease progression that would otherwise have occurred. As this protective effect would primarily affect severe dengue rather than moderate dengue, and most patients with moderate/severe dengue were included in our model development at the time they developed moderate dengue, its effect on our utility analysis would likely be small. In reality, the overall cost (in terms of apparently unnecessary admissions) of implementing our models as part of a hospitalisation algorithm would be lower – i.e., some proportion of the patients admitted in whom the outcome did not occur will have benefited from hospitalisation and might otherwise have progressed.

Furthermore, our utility analysis relies on the assumption that our models are perfectly implemented. Although implementation in low-resource clinical settings could be challenging, the fact that our models only require readily available clinical and laboratory variables should minimise such difficulties. Given the ever-increasing mobile phone coverage globally, integrating our models into a smartphone web application could also facilitate their use in clinical settings. Finally, in tertiary hospitals with electronic data management systems, these models could be integrated into patient monitoring devices that retrieve input automatically from real-time databases.

In summary, using serial observations from a prospective multicentre study across 8 countries, we have provided valuable characterisation of the clinical evolution of dengue in moderate/severe versus uncomplicated patients, and generated prediction models for moderate/severe dengue that incorporate clinical signs, symptoms and readily available haematological markers. We were able to provide a robust quantification of the effect of established warning signs and other clinical and laboratory variables, both using baseline information available at enrolment and incorporating daily updated information for each patient. Our examination of the utility of employing these models alongside clinicians’ intuitive prediction suggests the combined approach could benefit triage and management of dengue cases by increasing pre-emptive hospitalisation of individuals who subsequently develop complications. If validated and integrated into locally tailored decision-support systems with appropriate risk thresholds, these algorithms could provide major public health benefits in outbreak settings or in endemic areas with a high case burden. Incorporation of additional variables (potentially a marker of hepatic function, or a genetic, immunological or vascular biomarker with an established risk signature^17,45^), could further improve performance in settings with access to more sophisticated laboratory facilities. Finally, their use could enhance productivity of clinical trials of early therapeutic interventions by focusing enrolment on patients likely to develop complications.

## Methods

### Ethics Committee Approvals

We obtained overarching ethical approvals from 1) the Oxford Tropical Research Ethics Committee, Oxford University, UK, since the Oxford University Clinical Research Unit in Vietnam was responsible for coordinating all the Southeast Asian sites, and 2) Ethikkomission Medizinische Fakultät Heidelberg, University Hospital Heidelberg, Germany, since the Section Clinical Tropical Medicine, in the Department of Infectious Diseases, Heidelberg University Hospital was responsible for coordination across the Latin American sites and Bangladesh. We also obtained ethical approvals from the applicable institutional and national boards for each participating centre, to ensure compliance with all relevant regulations. A summary of the various committees that approved the initial study protocol and all subsequent amendments, is provided below.

**Table:** 

Site/Partner, Country	Issuing institution
-Angkor Hospital for Children, Cambodia	-Ethics Committee, Angkor Hospital for Children, Siem Reap, Cambodia
-Gadjah Mada University, Yogyakarta, Indonesia	-Medical and Health Research Ethics Committee, Faculty of Medicine, Gadjah Mada University, Indonesia
-The University of Malaya Medical Centre, Kuala Lumpur Malaysia	-Medical Ethics Committee, University of Malaya Medical Centre, Malaysia
-Ampang Hospital, Tengku Ampuan Rahimah Hospital, Klang Hospital, Kuala Lumpur, Malaysia	Ethics Committee, Clinical Research Center, National Institutes of Health, Ministry of Health, Malaysia Medical Research & Ethics Committee, Ministry of Health, Malaysia
-Children Hospital 2, Vietnam	-Ethics Committee, Children’s Hospital 2, Ho Chi Minh City, Vietnam
-Hospital for Tropical Diseases, Vietnam	-Ethics Committee, Hospital for Tropical Diseases, Ho Chi Minh City, Vietnam
-National Hospital for Tropical Diseases, Vietnam	-Ethics Committee, National Hospital for Tropical Diseases, Ha Noi, Vietnam
-ICDDR-B, Bangladesh	-Ethics Committee, ICDDR-B, Dhaka, Bangladesh
-Universidade Estadual do Ceará (UECE) and Fundação Oswaldo Cruz (FIOCRUZ) Brazil	Comite de Etica em Pesquisa, Fundacao Universidade Estadual do Ceara, Fortaleza Comissao Nacional de Éthica em Pesquisa, Ministry of Health, Brazil Comitâ de Ética em Pesquisa em Seres Humanos do Instituto de Medicina Integral Prof. Fernando Figueira, Recife Comitâ de Ética em Pesquisa Centro de Pesquisas Aggeu Magalhaes, Recife Comitâ de Ética em Pesquisa Instituto de Pesquisa Clinica Evandro Chagas, Rio de Janeiro
-Hospital Benjamin Bloom. El Salvador	-Comité de Ética en Investigation Clinica, Hospital Nacional de Ninos Benjamin Bloom, San Salvador
-The University of Carabobo, Venezuela	Fundación Carabobeno para la Salud, Valencia, Venezuela Instituto de Investigaciones Biomedicas, Maracay, Venezuela

All patients or a responsible parent/guardian gave written informed consent after discussion with study staff. Children aged 12–17 also provided assent using a separate form after discussion with study staff. For illiterate participants the consent or assent form was read aloud in the presence of a witness. All study documents were provided in the local language using easily understandable terms.

### Clinical and Laboratory Methods (Supplementary Methods 1-5)

Study staff enrolled participants aged 5 years or more attending clinics in 8 countries across Asia and Latin America (LA) with symptoms consistent with possible dengue (suspected dengue and/or undifferentiated fever in a patient from a dengue-endemic area) and fever for less than 3.5 days, provided they were considered suitable for outpatient management by the treating clinician. Exclusion criteria included (1) having localising signs or symptoms suggesting an alternative diagnosis, or (2) being considered unlikely to attend daily follow-up (e.g., due to travelling distance from the clinic), as judged by the treating physician.

Detailed information on study procedures is available in the published protocol^31^ and in the Supplementary Methods. In brief, we used a structured case report form (CRF) to collect clinical information at enrolment and then daily thereafter until recovery or for a maximum of six days, together with demographic information including age and sex (self or parent/guardian reported).

Routine laboratory tests and dengue diagnostic samples were obtained at designated intervals, and final diagnosis and immune status were defined using specific algorithms. All management decisions were the responsibility of treating physicians, not study staff. Study participants were asked to return for review approximately one week after the last acute illness assessment, when a summary of the illness episode was completed.

### Outcome definitions and candidate predictors (Supplementary Methods 6-7)

Initially our chosen clinical outcome of interest was severe dengue, strictly defined following WHO 2009 guidelines^7^. However, as this occurred infrequently in our outpatient-enrolled cohort, we subsequently elected to use moderate/severe dengue as the main clinical outcome. Moderate/severe dengue was defined as development of moderate/severe plasma leakage or moderate/severe bleeding or moderate/severe neurological involvement or moderate/severe liver involvement or moderate/severe involvement of another organ. For each component included within this diagnostic category we applied definitions consistent with the consensus on standard clinical endpoints for dengue intervention trials (Supplementary Methods 6 and Supplementary Table 2)^33^.

From a total of 65 variables recorded in the CRF, we selected a pre-defined list of 29 candidate predictors that (1) were not part of the outcome definitions, (2) had <10% missing data at baseline, (3) occurred in 5-95% of participants (for clinical signs and symptoms) and were readily available in clinical practice in participating centres (for laboratory tests). The definition of each candidate predictor is provided in Supplementary Table 3. To avoid multicollinearity, clinical signs/symptoms with strong correlations (overall agreement index > 0.7) were combined into single variables. Among the WHO 2009 warning signs the following variables were included: persistent vomiting, moderate to severe abdominal pain/tenderness, lethargy/restlessness, mucosal bleeding, and platelet count.

### Statistical Analysis

#### Analysis populations

In line with the specific objectives, we defined distinct analysis populations for each analysis (Figure 1). Detailed definitions and descriptions of these analysis populations are provided in Supplementary Table 4.

#### Univariable analysis

Associations between each candidate predictor at enrolment and subsequent moderate/severe dengue were assessed using univariable logistic regression in the univariable analysis population. Continuous predictors were modelled as linear terms. Results are presented in Table 2.

We also explored temporal trends in candidate predictors by outcome status. Participants who did not develop moderate/severe dengue contributed daily predictor data for the full follow-up period, whereas those who developed moderate/severe dengue contributed data up to the day before onset of the outcome. Categorical clinical signs and symptoms were summarised as daily proportions and continuous laboratory tests were summarised as daily means. Wald-type 95% confidence intervals were computed for each daily proportion and mean, and the findings are shown in Figure 3.

#### Model development and assessment procedures

Only patients with laboratory confirmed dengue were included in our main analyses, which are based on cases with complete data for the candidate predictors. After exploring heterogeneities in patient characteristics and outcomes between study sites, we developed 3 prediction models for moderate/severe dengue occurring at least 1 day after assessment, using the selected clinical and laboratory variables as candidate predictors. These included 1) a model using information collected at presentation only (Baseline Model), 2) a model using information from each single illness day separately to predict moderate/severe dengue on subsequent days (Dynamic-1 Model), and 3) a model additionally incorporating change in certain variables from the previous day to update risk prediction (Dynamic-2 Model).

For validation, given the limited number of outcome events, we opted for resampling validation rather than a split-sample approach. No comparable independent dataset was available to allow external validation. We assessed generalisability by evaluating performance across subpopulations (by continent and age), in patients with suspected rather than confirmed dengue, and by examining potential heterogeneities.

The model development procedure is in line with current recommendations^47,49^, and is summarised in Figure 4, with the following major steps.

#### (1) Preparation of analysis datasets

For each day of illness, we created a dataset with values recorded for that day plus, for some clinical and laboratory variables, the change from the previous day. Data from each of the six days of follow-up were stacked. For each participant, only information collected up to the day before the clinical outcome occurred was included in the three main datasets used for development of the prediction models: 1 baseline dataset that included only the values of the candidate predictors obtained at study enrolment, and 2 stacked datasets that included the values of the candidate predictors from all study days, as well as changes from the previous day’s assessment for relevant parameters. The final clinical outcome variable was included in all three datasets.

#### (2) Assessment of model assumptions

We used logistic regression to develop our prediction models. Linearity in the effects of continuous covariates, as well as potential interactions between covariates, were assessed based on two-sided likelihood ratio tests^47,49^. These tests were conducted separately for data from illness days 1, 2, and 3, and also for all the baseline data. For linearity test, a full model incorporating all continuous variables as restricted cubic splines with 4 degrees of freedom was compared against a reduced model, which treated the specific continuous variable of interest as a linear term while maintaining spline terms for the remaining variables (Supplementary Table 5). Non-linearities with a p-value <0.01 that were identified in any of the four models – i.e., the models for illness days 1, 2, or 3 separately, and the full baseline model – were retained. Simple non-linearities identified in this step (e.g., quadratic shape) were further approximated using piecewise linear splines to enhance interpretation; cut-points were chosen based on graphs of non-linear associations between these predictors and the outcome.

We considered pre-defined interactions between a) continent and (age, chills, lethargy/restlessness, dizziness, rash, and flush), and b) age and (lethargy/restlessness, vomiting, abdominal pain/tenderness, platelet count, total WBC, and % lymphocyte count). We compared models that included all pre-defined interactions with a model that excluded the interaction of interest, and then retained any interactions with a p-value < 0.01 in any of the four models (Supplementary Table 6). In these interactions, all continuous variables were included as linear terms only. Based on these analyses, we modelled platelet count and total WBC count non-linearly (with the restricted cubic splines simplified into piecewise linear splines after assessing graphical display of the non-linearities), and included three interactions, a) continent and age, b) continent and dizziness, and c) age and lethargy/restlessness.

#### (3) Assessment of heterogeneities

We explored heterogeneities in patient characteristics and outcomes between study sites (Figure 2 and Supplementary Table 13), as well as their potential impact on model performance (Supplementary Table 14), following the principles outlined by Steyerberg EW et al^50^. First, we described enrolment numbers, outcome prevalence, and age distribution by study site. Next, we explored differences in patient characteristics using membership c-statistics, which reflect the discriminative ability to separate a specific study site from all other sites combined, based on patient characteristics. To do this, we fitted a multinomial logistic regression model with site as the outcome and candidate predictors as covariates, using the baseline data. For each study site, we used this model to estimate the predicted probabilities of a patient being from that site. We then computed c-statistics by comparing these probabilities between patients who were and were not from the site. We then explored differences in predictions between study sites by computing and comparing the distribution of the linear predictors obtained by fitting the full baseline prediction model on all participants. To assess heterogeneity in prediction performance, we calculated estimates and 95% confidence intervals of performance criteria (AUC, calibration in-the-large, and calibration slope). This was done a) separately for each site with at least 10 events of moderate/severe dengue, and b) grouped by continent, using a baseline prediction model that was developed using data from all other sites or the other continent. The model used was a simplified version of the reduced baseline prediction model with stepwise variable selection using the Akaike Information Criterion; it only included age, persistent vomiting, pulse pressure, lymphocyte percentage, platelet count, and white blood cells (all modelled as linear terms, as described in the section above).

#### (4) Model fitting and variable selection

The baseline prediction model was fitted on the baseline dataset. Following the landmark approach described by Zheng and Heagerty^51^, we used the stacked datasets to fit the dynamic prediction models, thus allowing each predictor to interact with illness day to capture potential time-varying effects of the predictors. The dynamic prediction model using single time-point values of predictors (Dynamic-1) was fitted to stacked dataset 1. As the Dynamic-1 model uses only current data (from the clinical assessment on that day itself), we also developed a dynamic prediction model that incorporates data on change (Dynamic-2), comparing current data with data from the previous day’s assessment in the form of a change variable, in order to account for clinically relevant changes in signs, symptoms and laboratory variables. To limit model complexity, Dynamic-2 was not developed on the full set of candidate predictors in the same way as the Baseline and Dynamic-1 models, but instead was based only on variables included in the final Dynamic-1 model after variable selection. Since individual patients are represented multiple times in the stacked datasets, we used generalised estimating equations with a working independence variance-covariance matrix, to assess the 95% confidence intervals for associations between each predictor and the outcome in the dynamic models. The three full models were then simplified into final models using backward stepwise variable selection with the Akaike Information Criterion (for the final Baseline model) or a p-value of 0.15 (for the final Dynamic-1 and Dynamic-2 models) as the selection measure. Regression coefficients for the final models are provided in Table 3. The proof-of-concept web application for our models is provided at this address: https://lampk.shinyapps.io/IDAMSPRED/ (Supplementary Fig. 5).

#### (5) Assessment of model performance

Model performance was assessed in term of discrimination (AUC) and calibration (calibration-in-the-large and calibration slope) on different populations of interest. Discrimination measures the probability that a model can correctly classify a pair of patients with and without moderate/severe dengue based on their predictions, i.e., the probability to develop the outcome. A perfect model would have an AUC of 1. Calibration refers to the agreement between a model’s predictions and the observed outcomes. Calibration in-the-large indicates whether a model’s predictions are systematically too low or too high compared to the observed outcomes and the perfect value would be 0. The calibration slope reflects the average effect of the predictors on the outcome and the perfect value would be 1. As the validation dataset also formed part of the dataset used to develop the models, we corrected the apparent performance for over-optimism. Over-optimism was estimated through internal validation, in which most of the model development process (including variable selection, but excluding assessments of linearity and interaction) and evaluation were repeated 1000 times using bootstrapping^47^. Since the Baseline Model is only appropriate for use at first presentation, we assessed the performance of this model on the following datasets: all baseline data; baseline data from Asian children and adults separately; baseline data from Latin American children and adults separately. Model performance of the Dynamic-1 and Dynamic-2 Models was assessed at baseline and also on subsequent study days. As the interactions identified in our development procedure could complicate interpretation of relationships between predictors and outcome, we also applied the above stepwise procedure to models without interactions (i.e., ignoring the interaction assessment step). Supplementary Table 7 compares the performance of models with and without interactions. For ease of interpretation, only models without interactions are presented in Figure 5. However, the final models do include interactions; performance of these final models is presented in Figure 6.

#### (6) Additional exploratory models

To explore the potential added value of including immune status and serotype in predicting outcome, we subsequently added these variables into the 3 final models. We calculated the optimism-corrected AUCs for these models based on 1000 bootstrap samples for each day after enrolment, and compared the findings with the three original models (Supplementary Fig. 2-Panel A). Secondly, we applied the three main models in the suspected-dengue analysis population and explored changes in optimism-corrected AUCs compared to the lab-confirmed analysis population (Supplementary Figs. 3 & 4). To understand factors that might drive differences in model performance, we investigated differences in patient characteristics at enrolment by analysis population and by continent (Supplementary Table 15). We also explored differences in univariable relationships between each candidate predictor and the moderate/severe dengue outcome by analysis population, as well as by sub-populations defined by continent and age-group (Supplementary Tables 16-18). Finally, in order to compare the performance of our models with the published literature, we used the same data and the model development approach described above to fit models that include only (1) platelet count or (2) WHO’s warning signs (persistent vomiting, significant abdominal pain/tenderness, lethargy/restlessness, mucosal bleeding, platelet count ≤ 50K/mm3), with or without illness day at enrolment and/or the interaction between age and continent (Supplementary Fig. 2-Panel B).

#### (7) Assessment of model utility to improve decision-making around hospitalisation

We defined decision rules for hospitalisation based on our developed models, and also on several simple rules that relied on the number of warning signs present up to the day of assessment. Decision rules for hospitalisation involving our developed models depend on a risk threshold or cut-off at which the predicted risk of the outcome is deemed sufficient to warrant admission; this threshold may vary among clinicians and health systems. Therefore, we varied the risk threshold from 0% to 100% to explore the whole potential performance of these decision rules. For this assessment, we assumed that hospitalisation does not change the course of the illness (which may not be true), so that we could calculate and compare the sensitivity and specificity to detect the moderate or severe outcome at least one day in advance (Figure 7-Panels A & B). Using the trade-off between the cost of admitting patients who never progress to the moderate/severe outcome and the cost of missing patients who do develop the moderate/severe outcome, we calculated the net benefit of hospitalisation. We compared the net benefit of six different strategies, including hospitalisation for no-one, hospitalisation for all, hospitalisation based on the clinicians’ decision alone, and hospitalisation based on different combinations of the clinicians’ decision with our developed models (Figure 7-Panel C, Supplementary Fig. 6).

#### (8) Sensitivity analysis to explore the potential impact of missing data

We used the mode (for categorical variables) and median (for continuous variables) to impute missing values of the candidate predictors at baseline, as well as the last observation carried forward approach to impute missing data from the sequential daily observations for each patient. Potential impacts of missing data on the model assumptions, estimated coefficients, and model performance were assessed by comparing results from the imputed dataset and the main analysis, and are presented in Supplementary Tables 10, 11, 12 and Supplementary Fig. 1.

All analyses were performed using R version 4.0.4^52^ and its companion packages^53–58^.

## Supplementary Material

Peer Review File

Reporting Summary

Source Data

Supplementary Document

## Figures and Tables

**Table 1:** Enrolment characteristics and clinical outcomes for participants with confirmed dengue included in the descriptive analysis population (n = 2230).

Characteristic		Asia (n = 1850)			Latin America (n = 380)				Total (n = 2230)
	n	Summary		n		Summary		n	Summary
Country	1850			380				2230	
Cambodia		223 (12)				0 (0)			223 (10)
Indonesia		39 (2)				0 (0)			39 (2)
Malaysia		205 (11)				0 (0)			205 (9)
Vietnam		1326 (72)				0 (0)			1326 (59)
Bangladesh		57 (3)				0 (0)			57 (3)
Brazil		0 (0)				54 (14)			54 (2)
El Salvador		0 (0)				296 (78)			296 (13)
Venezuela		0 (0)				30 (8)			30 (1)
Year of enrolment	1850			380				2230	
2011		108 (6)				0 (0)			108 (5)
2012		420 (23)				38 (10)			458 (21)
2013		376 (20)				144 (38)			520 (23)
2014		454 (25)				75 (20)			529 (24)
2015		492 (27)				123 (32)			615 (28)
Age [years]	1850	17 (10, 26)		380		9 (7, 12)		2230	14 (9, 24)
Child (<15 years old)	1850	827 (45)		380		324 (85)		2230	1151 (52)
Sex: Female	1850	776 (42)		380		158 (42)		2230	934 (42)
Illness day at enrolment	1850			380				2230	
1		300 (16)				60 (16)			360 (16)
2		776 (42)				130 (34)			906 (41)
3		774 (42)				190 (50)			964 (43)
Viremia (if detectable) [log-10 copies/mL]	1717	7·4 (6·2, 8·3)		234		7·1 (5·7, 7·9)		1951	7·3 (6·2, 8·2)
Viremia below detection limit	1840	123 (7)		379		81 (21)		2219	204 (9)
Viral serotype	1717			265				1982	
DENV-1		834 (49)				48 (18)			882 (45)
DENV-2		287 (17)				15 (6)			302 (15)
DENV-3		111 (6)				200 (75)			311 (16)
DENV-4		485 (28)				2 (1)			487 (25)
Immune status	1850			380				2230	
Probable primary infection		377 (20)				124 (33)			501 (22)
Probable secondary infection		1239 (67)				102 (27)			1341 (60)
Inconclusive serostatus		234 (13)				154 (40)			388 (17)
Hospitalised for medical reasons	1850	629 (34)		380		14 (4)		2230	643 (29)
Illness day at hospitalisation	629			14				643	
1-3		93 (15)				3 (21)			96 (15)
4		218 (35)				4 (29)			222 (35)
5		190 (30)				5 (36)			195 (30)
6		108 (17)				1 (7)			109 (17)
≥7		20 (3)				1 (7)			21 (3)
Intravenous fluid given	1850	576 (31)		380		133 (35)		2230	709 (32)
Illness day at intravenous fluid	576			133				709	
1-3		103 (18)				39 (29)			142 (20)
4		188 (33)				53 (40)			241 (34)
5		160 (28)				25 (19)			185 (26)
6		105 (18)				8 (6)			113 (16)
≥7		20 (3)				8 (6)			28 (4)
Final severity grade	1850			380				2230	
Severe		32 (2)				6 (2)			38 (2)
Moderate		242 (13)				24 (6)			266 (12)
Uncomplicated		1576 (85)				350 (92)			1926 (86)
Illness day at moderate/severe dengue	274			30				304	
1-3		22 (8)				9 (30)			31 (10)
4		40 (15)				9 (30)			49 (16)
5		78 (28)				7 (23)			85 (28)
6		80 (29)				3 (10)			83 (27)
≥7		54 (20)				2 (7)			56 (19)
Illness day at severe dengue	32			6				38	
1-3		1 (3)				1 (17)			2 (5)
4		2 (6)				2 (33)			4 (11)
5		11 (34)				1 (17)			12 (32)
6		12 (38)				2 (33)			14 (37)
7		6 (19)				0 (0)			6 (16)

**Legend for Table 1** N is the number of all patients; n is the number of patients with non-missing values. Summary is the absolute count (%) for categorical variable(s) and median (IQR) for continuous variable(s). This table includes 24 patients in whom the outcome occurred on the enrolment day. Excluding these 24 participants leaves a total of 280 patients with moderate/severe dengue, among whom 37 were severe.

**Table 2:** Univariable and multivariable analysis (without interactions) for moderate/severe dengue using enrolment information (n = 2206)

Covariate at enrolment	Moderate/severe dengue					Univariable analysis				Multivariable analysis		
	No (1926, 87%)		Yes (280, 13%)									
	n	Summary	n	Summary		OR	(95% CI)	P		OR	(95% CI)	p
Continent: Latin America	1926	350 (18)	280	24 (9)		0.42	(0.27, 0.65)	9 × 10^−5^		0.68	(0.38, 1.22)	0.194
Age (per 5 years)	1926	14 (9, 24)	280	16 (10, 24)		1.10	(0.98, 1.21)	0.118		0.93	(0.85, 1.01)	0.095
Female	1926	813 (42)	280	114 (41)		0.94	(0.73, 1.21)	0.635		1.07	(0.81, 1.43)	0.619
Past medical history	1911	318 (17)	280	41 (15)		0.86	(0.60, 1.22)	0.399		1.00	(0.68, 1.48)	0.988
Chills	1923	1580 (82)	280	231 (82)		1.02	(0.74, 1.42)	0.891		0.66	(0.46, 0.96)	0.031
Lymphadenopathy	1926	182 (9)	280	22 (8)		0.82	(0.52, 1.30)	0.391		1.02	(0.61, 1.71)	0.945
Lethargy/restlessness	1923	82 (4)	280	17 (6)		1.45	(0.85, 2.49)	0.175		1.04	(0.57, 1.89)	0.907
Cough	1926	468 (24)	280	81 (29)		1.27	(0.96, 1.68)	0.095		1.12	(0.82, 1.52)	0.488
Rhinitis	1925	166 (9)	280	32 (11)		1.37	(0.92, 2.04)	0.126		1.23	(0.80, 1.91)	0.348
Conjunctival injection	1926	841 (44)	280	155 (55)		1.60	(1.24, 2.06)	0.0002		1.24	(0.89, 1.74)	0.198
Dizziness	1922	881 (46)	280	152 (54)		1.40	(1.09, 1.80)	0.008		1.13	(0.85, 1.49)	0.398
Headache	1926	1709 (89)	280	258 (92)		1.49	(0.94, 2.35)	0.088		1.17	(0.70, 1.95)	0.542
Retro-orbital pain	1924	739 (38)	279	116 (42)		1.14	(0.88, 1.47)	0.311		0.94	(0.70, 1.26)	0.665
Joint/muscle pain	1925	1382 (72)	280	219 (78)		1.41	(1.04, 1.91)	0.025		1.40	(0.96, 2.04)	0.077
Persistent vomiting	1925	677 (35)	280	115 (41)		1.28	(0.99, 1.66)	0.055		1.50	(1.12, 2.01)	0.007
Diarrhoea	1926	264 (14)	280	40 (14)		1.05	(0.73, 1.50)	0.793		1.00	(0.67, 1.48)	0.988
Significant abdominal pain/tenderness	1919	135 (7)	280	24 (9)		1.24	(0.79, 1.95)	0.355		1.57	(0.93, 2.63)	0.089
Skin rash	1917	278 (15)	278	25 (9)		0.58	(0.38, 0.90)	0.014		1.10	(0.66, 1.81)	0.718
Skin flush	1926	838 (44)	279	125 (45)		1.05	(0.82, 1.36)	0.684		0.92	(0.66, 1.30)	0.643
Bleeding	1926		280					0.070				0.093
None		1640 (85)		252 (90)		1.00	-	-		1.00	-	-
Skin only		196 (10)		18 (6)		0.60	(0.36, 0.99)	0.044		0.56	(0.32, 0.98)	0.041
Mucosal bleeding		90 (5)		10 (4)		0.72	(0.37, 1.41)	0.340		0.83	(0.41, 1.70)	0.613
Temperature (per 1° Celsius increase)	1925	38 (38, 39)	280	39 (38, 39)		1.31	(1.12, 1.52)	0.0005		1.06	(0.88, 1.28)	0.542
Respiratory rate (per 5 breaths/min increase)	1922	22 (20, 24)	280	22 (20, 24)		1.05	(0.88, 1.25)	0.607		1.00	(0.79, 1.26)	0.992
Pulse (per 10 beats/min increase)	1924	98 (88, 108)	280	100 (88, 110)		1.02	(0.93, 1.12)	0.640		1.00	(0.90, 1.12)	0.978
Systolic blood pressure (per 10 mmHg increase)	1920	105 (100, 110)	280	109 (100, 112)		1.06	(0.96, 1.19)	0.194		0.98	(0.83, 1.16)	0.828
Pulse pressure (per 10 mmHg increase)	1920	40 (35, 46)	280	40 (38, 50)		1.17	(1.02, 1.32)	0.020		1.14	(0.93, 1.39)	0.209
Platelet count (per 10,000 cells/mm3 decrease)	1918	166 (128, 208)	278	145 (104, 190)				3 × 10^−6^				1 × 10^−7^
If platelet count < 150,000		-		-		1.12	(1.06, 1.18)	4 × 10^−6^		1.16	(1.09, 1.23)	2 × 10^−7^
If platelet count ≥ 150,000		-		-		1.01	(0.98, 1.04)	0.426		1.02	(0.98, 1.06)	0.233
Total WBC (per 2 fold increase)	1917	4 (3, 6)	278	4 (3, 6)				0.026				0.003
If total WBC < 2,000		-		-		0.11	(0.02, 0.54)	0.004		0.07	(0.01, 0.38)	0.001
If total WBC ≥ 2,000		-		-		1.14	(0.92, 1.40)	0.226		0.95	(0.69, 1.30)	0.745
% Lymphocyte (per 10% increase)	1909	22 (14, 31)	277	17 (11, 25)		0.67	(0.59, 0.76)	1 × 10^−10^		0.64	(0.54, 0.75)	2 × 10^−7^
Illness day at enrolment	1926		280					0.015				0.066
1		304 (16)		53 (19)		1.00	-	-		1.00	-	-
2		767 (40)		128 (46)		0.96	(0.68, 1.35)	0.805		1.02	(0.69, 1.53)	0.911
3		855 (44)		99 (35)		0.66	(0.46, 0.95)	0.025		0.70	(0.43, 1.12)	0.137
**Additional variables (not included in prediction models)**												
Immune status: secondary infection	1567	1110 (71)	257	217 (84)		2.23	(1.57, 3.18)	9 × 10^−6^		-	-	-
Viral serotype	1704		258					0.101				-
DENV-1		755 (44)		116 (45)		1.00	-	-		-	-	-
DENV-2		254 (15)		45 (17)		1.15	(0.79, 1.67)	0.453		-	-	-
DENV-3		279 (16)		28 (11)		0.65	(0.42, 1.01)	0.055		-	-	-
DENV-4		416 (24)		69 (27)		1.08	(0.78, 1.49)	0.640		-	-	-

Summary is the absolute count (%) for categorical variable(s) and median (1^st^ and 3^rd^ quartiles) for continuous variable(s). Odds ratios (OR), 95% confidence intervals (95% CI), and p values were calculated using logistic regression models with moderate/severe dengue as outcome. P-values are two-sided without adjustment for multiple comparisons. These results are based on the 2206 patients included in the univariable analysis population (i.e., patients who developed the outcome on the day of enrolment were excluded). The multivariable analysis was based on all 2130 patients with complete data available for all candidate predictors in our prediction models (i.e., not including immune status and serotype). Note that data were missing for immune status (inconclusive), and serotype, in 382 and 244 cases respectively. Platelet count and total white cell count (WBC) were modelled as linear terms in the univariable models and as piecewise linear splines in the multivariable regression model.

**Table 3:** Regression coefficients of the developed models.

Intercept and Predictors	Logistic regression coefficients (95% confidence interval)		
	Baseline Model	Dynamic-1 Model	Dynamic-2 Model
Intercept	-5.46 (-7.30, -3.61)	-6.33 (-9.1, -3.56)	-11.34 (-17.78, -4.9)
Continent (Latin America)	0.05 (-0.56, 0.66)	-0.16 (-0.75, 0.43)	-0.36 (-0.98, 0.26)
Age	-0.14 (-0.22, -0.05)	-0.15 (-0.24, -0.06)	-0.15 (-0.25, -0.05)
Chills	-0.36 (-0.73, 0.02)	-0.61 (-1.19, -0.03)	-
Lethargy/restlessness	-0.10 (-0.73, 0.52)	-0.36 (-1.3, 0.58)	0.47 (-0.03, 0.98)
Cough	-	-0.52 (-1.07, 0.03)	-1.25 (-2.1, -0.4)
Rhinitis	-	0.63 (-0.14, 1.41)	-
Conjunctival injection	0.26 (-0.04, 0.55)	0.21 (-0.06, 0.48)	0.32 (-0.01, 0.65)
Dizziness	0.25 (-0.04, 0.53)	-0.03 (-0.52, 0.47)	-0.07 (-0.92, 0.79)
Headache	-	-0.16 (-0.78, 0.45)	-0.2 (-1.09, 0.69)
Joint/muscle pain	0.40 (0.03, 0.76)	0.17 (-0.11, 0.45)	-
Persistent vomiting	0.38 (0.09, 0.68)	0.08 (-0.37, 0.53)	-0.03 (-0.93, 0.88)
Diarrhoea	-	0.19 (-0.08, 0.46)	0.21 (-0.13, 0.54)
Significant abdominal pain/tenderness	0.48 (-0.03, 1.00)	0.26 (-0.02, 0.55)	-0.24 (-0.82, 0.35)
Bleeding (Skin only)	-0.61 (-1.15, -0.06)	-1.1 (-1.89, -0.31)	-1.17 (-2.3, -0.04)
Bleeding (Mucosal bleeding)	-0.16 (-0.86, 0.55)	0.11 (-0.81, 1.03)	0.09 (-1.32, 1.49)
Temperature	-	0.01 (-0.27, 0.28)	0.3 (0.14, 0.45)
Pulse	-	-0.15 (-0.32, 0.02)	-0.33 (-0.62, -0.05)
Pulse pressure	0.05 (-0.02, 0.12)	0.09 (-0.05, 0.22)	0.04 (-0.19, 0.27)
% Lymphocyte	-0.22 (-0.31, -0.14)	-0.29 (-0.42, -0.17)	-0.43 (-0.62, -0.24)
Illness day at enrolment (day 2)	0.03 (-0.37, 0.43)	-0.12 (-0.51, 0.28)	-0.02 (-0.45, 0.41)
Illness day at enrolment (day 3)	-0.36 (-0.83, 0.11)	-0.66 (-1.08, -0.24)	-0.59 (-1.06, -0.12)
Platelet count (when ≤ 150,000)	-0.15 (-0.20, -0.09)	-0.13 (-0.21, -0.04)	-0.1 (-0.15, -0.05)
Platelet count (when > 150,000)	-0.02 (-0.06, 0.02)	-0.07 (-0.14, 0)	-0.02 (-0.08, 0.04)
Total WBC (when ≤ 2,000)	-2.35 (-3.99, -0.71)	-4.46 (-7.08, -1.85)	-8.76 (-15.05, -2.47)
Total WBC (when > 2,000)	-0.01 (-0.32, 0.29)	-0.23 (-0.68, 0.23)	-0.6 (-1.44, 0.23)
Continent (Latin America) * Age	0.34 (0.15, 0.53)	0.33 (0.13, 0.52)	0.35 (0.11, 0.6)
Continent (Latin America) * Dizziness	-1.16 (-2.19, -0.13)	-0.66 (-1.59, 0.27)	-
Age * Lethargy/restlessness	0.31 (0.00, 0.62)	0.46 (0, 0.91)	-
Day of illness	-	0.5 (-0.16, 1.16)	1.23 (-0.06, 2.51)
Day of illness * Chills	-	0.16 (0.01, 0.32)	-
Day of illness * Lethargy/restlessness	-	0.18 (-0.08, 0.43)	-
Day of illness * Cough	-	0.18 (0.04, 0.33)	0.34 (0.14, 0.54)
Day of illness * Rhinitis	-	-0.21 (-0.45, 0.04)	-
Day of illness * Dizziness	-	0.09 (-0.05, 0.24)	0.09 (-0.13, 0.32)
Day of illness * Headache	-	0.08 (-0.07, 0.24)	0.09 (-0.13, 0.3)
Day of illness * Persistent vomiting	-	0.11 (-0.02, 0.24)	0.09 (-0.12, 0.3)
Day of illness * Bleeding (Skin only)	-	0.22 (0.04, 0.4)	0.25 (0.01, 0.49)
Day of illness * Bleeding (Mucosal bleeding)	-	-0.14 (-0.35, 0.08)	-0.12 (-0.42, 0.18)
Day of illness * Temperature	-	0.06 (-0.01, 0.14)	-
Day of illness * Pulse	-	0.04 (-0.02, 0.09)	0.07 (-0.01, 0.14)
Day of illness * Pulse pressure	-	-0.03 (-0.07, 0.01)	-0.02 (-0.09, 0.04)
Day of illness * % Lymphocyte	-	0.03 (0, 0.06)	0.04 (0, 0.08)
Day of illness * Platelet count (when > 150,000)	-	0.02 (0, 0.03)	-
Day of illness * Platelet count (when ≤ 150,000)	-	0 (-0.02, 0.02)	-
Day of illness * Total WBC (when ≤ 2,000)	-	0.91 (0.28, 1.54)	1.55 (0.3, 2.8)
Day of illness * Total WBC (if > 2,000)	-	-0.03 (-0.14, 0.09)	0.07 (-0.12, 0.26)
Age * Day of illness * Lethargy/restlessness	-	-0.08 (-0.2, 0.03)	-
Change in persistent vomiting (already occurred)	-	-	0.34 (0.06, 0.61)
Change in persistent vomiting (new occur)	-	-	0.12 (-0.37, 0.6)
Change in significant abdominal pain/tenderness (already occurred)	-	-	0.37 (-0.06, 0.81)
Change in significant abdominal pain/tenderness (new occur)	-	-	0.53 (-0.09, 1.16)
Change in % Lymphocyte	-	-	0.1 (0.04, 0.16)
Change in Platelet count (when ≤ 150,000)	-	-	0.09 (-0.07, 0.25)
Change in Platelet count (when > 150,000)	-	-	0.01 (-0.07, 0.1)
Change in Total WBC (when ≤ 2,000)	-	-	4.85 (0.66, 9.05)
Change in Total WBC (when > 2,000)	-	-	0.03 (-0.71, 0.78)
Day of illness * Change in Platelet count (when ≤ 150,000)	-	-	-0.04 (-0.08, 0)
Day of illness * Change in Platelet count (when > 150,000)	-	-	-0.02 (-0.03, 0)
Day of illness * Change in Total WBC (when ≤ 2,000)	-	-	-0.72 (-1.48, 0.04)
Day of illness * Change in Total WBC (when > 2,000)	-	-	-0.03 (-0.2, 0.15)

**Legend for Table 3**

* Interaction between variables -Regression coefficient not relevant to that model or not selected in the development process WBC: total white cell count The predicted probability of moderate/severe dengue according to illness day (up to illness day 8) can be calculated using the following formula: Pr(moderate/severe dengue) = 1/(1 + exp(- linear predictor)), where the linear predictor is the product of the logistic regression coefficients provided in this table and the actual values of the predictors.

## Data Availability

Data will be deposited at the European Genome-phenome Archive (EGA), which is hosted by the European Molecular Biology Laboratory-European Bioinformatics Institute and the Centre for Genomic Regulation. Public deposition of the full dataset is in progress and data from sites with fully executed Data Transfer Agreements are being uploaded on a rolling basis, with the complete dataset anticipated to be available within the coming months. In the interim, the IDAMS data are available under restricted access, as the original informed consent did not include explicit language for public data sharing, necessitating additional ethics committee approvals and legal agreements across multiple participating sites. Requests to access the dataset for research aligned with the scope of the initial study (e.g., dengue or other arbovirus research) will be considered by the Data Access Committee at the Oxford University Clinical Research Unit in Ho Chi Minh City (https://www.oucru.org/data-sharing-policy/; dac@oucru.org). Additional source data for data-dependent figures are provided with this paper in the Source Data file.

## Code availability

All analyses were performed using R version 4.0.4 and its companion packages. All codes for analyses are available at this GitHub repository: https://github.com/lampk/IDAMS_prediction^59^.
