# Supplementary material for: Improving early prediction for adverse dengue outcomes using data from the IDAMS prospective multicentre study
Source: Nat Commun. Author manuscript; Available in PMC 2026 Sep 26. (PMC13590484; doi:10.1038/s41467-026-76450-2)
Supplement: Supplementary file 1 [file EMS218154-supplement-Peer_Review_File.pdf]

nature portfolio

Peer Review File

Improving early prediction for adverse dengue outcomes using data from the IDAMS prospective multicentre study

Corresponding Author: Dr Lam Phung Khanh

This manuscript has been previously reviewed at another journal. This document only contains information relating to versions considered at Nature Communications.

This file contains all reviewer reports in order by version, followed by all author rebuttals in order by version.

Version 1:

Reviewer comments:

Reviewer #1

(Remarks to the Author)
The revised manuscript adequately addresses the issues highlighted in the previous review.

(Remarks on code availability)
Not applicable

Reviewer #2

(Remarks to the Author)
The authors have sufficiently addressed my comments.

(Remarks on code availability)

Reviewer #3

(Remarks to the Author)
The authors have addressed all the concerns raised during the previous round of review in a comprehensive and professional manner. The revised manuscript significantly improves upon the original submission by incorporating several key methodological and presentational enhancements.

Key Improvements and Responses:

Methodological Rigor: The authors have added a Decision Curve Analysis (DCA) in Figure 7 (Panel C), which provides a clearer understanding of the clinical utility of the proposed models compared to standard clinical decision rules.

Transparency and Accessibility: The regression coefficients for the Baseline, Dynamic-1, and Dynamic-2 models have been moved from the supplementary material to the main text (Table 3). This change greatly enhances the transparency of the model development process. Furthermore, the functional web application link has been restored, allowing for practical evaluation of the tool.

Data Analysis: The inclusion of a sensitivity analysis using multiple imputations for missing data addresses the previous concerns regarding potential bias. The results demonstrate that the findings are robust and not significantly affected by the missingness in the dataset.

Clarification of Definitions: The authors provided a sound justification for the use of a combined "moderate/severe dengue" outcome, citing the clinical relevance and the statistical necessity due to the rarity of isolated severe cases in the study population.

Structural Adjustments: Several critical tables and figures were moved from the supplement to the main manuscript as requested, making the paper more self-contained and easier to follow for the reader.

The authors have demonstrated a high level of responsiveness to the reviewers' feedback. The additional analyses and clarifications have strengthened the manuscript's conclusions and clinical relevance. I believe the paper is now suitable for publication in its current form.

(Remarks on code availability)

Open Access This Peer Review File is licensed under a Creative Commons Attribution 4.0 International License, which permits use, sharing, adaptation, distribution and reproduction in any medium or format, as long as you give appropriate credit to the original author(s) and the source, provide a link to the Creative Commons license, and indicate if changes were made.
In cases where reviewers are anonymous, credit should be given to 'Anonymous Referee' and the source.
The images or other third party material in this Peer Review File are included in the article’s Creative Commons license, unless indicated otherwise in a credit line to the material. If material is not included in the article’s Creative Commons license and your intended use is not permitted by statutory regulation or exceeds the permitted use, you will need to obtain permission directly from the copyright holder.
To view a copy of this license, visit https://creativecommons.org/licenses/by/4.0/

Point-by-point rebuttal to reviewer comments

Black : reviewer comment
Blue : response to reviewer
Red : updated text in manuscript
Green : line numbers of updated text

REVIEWER #1

Early identification of individuals at risk of severe dengue is crucial for effective case management and resource allocation in high-burden areas. The manuscript discusses a multicenter study aimed at improving early prediction of severe dengue outcomes using clinical and hematological data from a large cohort of suspected dengue cases. The study involved 7428 suspected dengue cases across 8 countries in Asia and Latin America; 2694 (37%) confirmed dengue cases and of these 2230 individuals had sufficient data for analysis. The authors used multiple modelling approaches for predicting outcomes.

Comments:

1. Abstract: Some data may be provided to support the statements.

Response 1.1: Thank you for the comment. We have modified the Abstract to include data and numerical results to support our statements.

> *"Early identification of individuals at risk of complications is important to improve dengue case-management and promote appropriate use of limited resources in high burden settings. In this large prospective observational cohort, 7428 febrile outpatients were enrolled early and followed daily across 8 countries in Asia and Latin America; 2230 individuals with confirmed dengue were included in the analysis, among whom 304 (14%) progressed to moderate/severe dengue and 38 (2%) to severe dengue during follow-up. We demonstrate relationships that evolve over time between common clinical features, WHO clinical warning signs, and simple haematological parameters, with progression. Key predictors of moderate/severe dengue include low lymphocyte percentage, low total white blood cell count, low platelet count, and persistent vomiting and significant abdominal pain/tenderness. Multivariable models incorporating these variables predict the risk of moderate/severe dengue, initially with moderate performance (AUC~0.68), but improving subsequently as new daily data are included into the predictions (AUC~0.73-0.85). We also demonstrate that using these models in combination with clinical decision-making pathways could facilitate earlier admission of high-risk patients without markedly increasing admissions of uncomplicated cases. These results will be instrumental for updating guidelines on dengue triage and management, potentially providing major public health benefits in resource poor settings." (lines 2-17)*

2. The authors are requested to justify the change in the definition of the outcome – from severe dengue to moderate/ severe dengue. There is lack of clarity about the criteria used for defining moderate dengue. The cited reference (33) proposes criteria for severity of various endpoints- plasma leakage, bleed, liver disease, thrombocytopenia, etc but does not explicitly mention definition of moderate dengue. The authors have chosen to define moderate/severe dengue by presence of one or more of moderate/severe endpoints. However, such a definition for moderate dengue is not validated. Presence of multiple moderate endpoints cannot be equated with a single moderate endpoint.

Response 1.2: In the Discussion of this paper we explained that the outcome was changed to moderate/severe dengue as the number of severe cases was small, reflecting the early recruitment and clinical heterogeneity of dengue. Reference 33 states in the Discussion that "achieving any one severe or moderate endpoint during a

laboratory-confirmed dengue illness would be sufficient to designate the case as severe or moderate, respectively." While we agree that this definition of moderate dengue has not been formally validated, the same limitation applies to all other dengue outcome classification systems, including severe dengue and DF/DHF/DSS. We also acknowledge that the presence of multiple moderate endpoints could indicate greater severity than a single moderate endpoint, but since our primary outcome is defined as moderate or severe dengue, this distinction would not be expected to affect the analysis.

We have added an explanatory sentence about timing to the Results section, and modified the Discussion to acknowledge the fact that our findings might be more applicable to moderate dengue than severe dengue as most patients with moderate/severe dengue were actually analysed when they developed moderate dengue.

> *"Among the patients with severe dengue, 13 of 38 developed moderate disease 1-2 days before progressing to severe dengue, another 13 progressed to severe dengue on the same day, while the remaining 12 patients progressed directly to severe dengue without an identifiable moderate stage." (lines 111-114)*
>
> *"Recognising that we could not feasibly conduct a robust analysis involving many candidate predictors, we elected to use instead the combined endpoint of moderate/severe dengue. However, as most patients with moderate/severe dengue were included in the model development when they developed moderate dengue (250/274, 91%), our findings may be more applicable to this outcome than severe dengue. Future research could potentially be conducted on pooled cohort data from this study and other observational cohorts (eg. control arms of vaccine trials) in order to repeat the analysis with a larger sample size and severe dengue as the outcome." (lines 328-335)*

3. While the protocol mentions inclusion of subjects with fever of upto 72 hrs, the manuscript mentions including those with duration of upto 3.5 days. This modification needs justification. How was this cut-off practically implemented?

Response 1.3: While the protocol used an inclusion criteria of fever up to 72 hrs, we modified this threshold to 3.5 days to align with the practical working day – i.e. individuals presenting on the third febrile day to study outpatient facilities during the clinics' standard working hours were enrolled, even if the fever had started during the night before the first febrile day. We provide justification for this modification in Table S1 in the Supplementary Information:

> *"Although the inclusion criteria limited enrolment to within 72 hours of fever onset, in practice recruitment was aligned with the working day; 87 patients who enrolled between 72-84 hours from fever onset (i.e. within 3.5 days) were considered as enrolling on illness day 3." (page 3 Supplemtary Information)*

4. It is important to know about the treatment advised and received by the study subjects. Even though there is no specific treatment for dengue, appropriate fluid therapy may affect the outcomes. There is lack of clarity whether this aspect was included in the modelling.

Response 1.4: The proportion of patients who received IV fluid during the study is reported in Table 1. As noted in the definition of candidate predictors, no variables related to fluid intervention were included in our models. Incorporating fluid therapy appropriately into a prediction model would require very detailed information on daily fluid usage. However, all management decisions were at the discretion of the responsible clinician and varied between the study sites. Since detailed information was not available we chose not to include this in the current models. However, we agree that the influence of fluid therapy remains an important topic for future studies.

5. There is discrepancy in number of clinical and lab parameters studied- mentioned as 24 and 27 and different places in the text (29 listed in table A.7).

Response 1.5: We included a total of 29 variables as candidate predictors (listed in Table A.7), of which 24 are clinical signs, symptoms, or laboratory tests. However, a total of 27 variables are included in the description of

data missingness, as two variables (continent and illness day at enrolment) were available by design for all patients. We have reconciled the numbers and revised the text generally to improve clarity for readers.

> *"From a total of 65 variables available in the database, 29 were considered as candidate predictors. To be selected, these variables must…" (page 6 Supplemtary Information)*

6. It will be good to present the actual prediction model which a practitioner could use rather than presenting the Ors of various components of the model in the figures.

Response 1.6: Originally we presented the model coefficients, developed a web application, and provided a screenshot of the application in the Supplementary Information. Unfortunately however, during the blinding process for peer review the link to the web application was removed from the manuscript, for which we apologize sincerely. This information has now been restored.

Risk score or point-based algorithms are generally considered less useful than web/computer-based applications, as they offer lower precision and are less practical for complex models (Moon KGM et al., 2015, http://dx.doi.org/10.7326/M14-0698). Therefore, we prefer to provide the detailed model coefficients along with the web application for prediction calculations.

To enhance visibility, we have moved the final regression coefficients from the Supplementary Information to the main Table 3, and provide this link to the web application,

> *"A demonstration of the web application for the three models developed can be accessed at this link: https://lampk.shinyapps.io/IDAMSPRED/ (SupplementaryInformation _FigureS5)." (lines 184-186)*

We also include instructions on how to calculate predictions using the model coefficients provided.

> *"The predicted probability of moderate/severe dengue according to illness day (up to illness day 8) can be calculated using the following formula: Pr(moderate/severe dengue) = 1/(1 + exp(- linear predictor)), where the linear predictor is the product of the logistic regression coefficients provided in this table and the actual values of the predictors." (lines 510-513, footnote for Table 3)*

7. The references appear to be incorrectly numbered in the manuscript. For example, the reference to the Supplementary Methods is cited as number 24, whereas in the actual reference list it appears as number 31. Kindly review and correct the numbering of all references to ensure consistency and accuracy throughout the manuscript.

Response 1.7: We have reviewed the numbering of references to ensure consistency and corrected the reference number that refers to the published protocol:

> *"Detailed information on study procedures is available in the published protocol[31]" (line 582)*

REVIEWER #2

This manuscript describes the development of baseline and dynamic clinical prediction models for adverse dengue outcomes using data from a large, prospective multicenter cohort study. This is an important and timely contribution. The dataset is among the largest of its kind, the prospective design with serial daily data is a clear strength, and the focus on outpatient cases increases relevance for real-world clinical decision-making.

Major comments

1) The authors combine moderate and severe dengue into a single outcome for prediction. I get this, given that severe is more rare, but still wonder if this might obscure clinically important differences. Many of the predictors may be strongly associated with plasma leakage or thrombocytopenia but not with the most severe outcomes such as shock, organ failure or bleeding. Even if exploratory, I think the paper would be strengthened by

stratification or at a minimum, acknowledging that the predictors identified may largely be reflective of risk of moderate disease.

Response 2.1: Thank you for the comment. We agree that our findings, including predictors and developed models, are applicable more to moderate dengue than severe dengue. As noted above under Response 1.2 we have added an explanatory sentence about timing to the Results section, and modified the Discussion to acknowledge the fact that our findings might be more applicable to moderate dengue than severe dengue as most patients with moderate/severe dengue were actually analysed when they developed moderate dengue.

> *"Among the patients with severe dengue, 13 of 38 developed moderate disease 1-2 days before progressing to severe dengue, another 13 progressed to severe dengue on the same day, while the remaining 12 patients progressed directly to severe dengue without an identifiable moderate stage." (lines 111-114)*

> *"Recognising that we could not feasibly conduct a robust analysis involving many candidate predictors, we elected to use instead the combined endpoint of moderate/severe dengue. However, as most patients with moderate/severe dengue were included in the model development when they developed moderate dengue (250/274, 91%), our findings may be more applicable to this outcome than severe dengue. Future research could potentially be conducted on pooled cohort data from this study and other observational cohorts (eg. control arms of vaccine trials) in order to repeat the analysis with a larger sample size and severe dengue as the outcome." (lines 328-335)*

2) The fact that immune status doesn't improve model performance is surprising. Secondary infections are, after all, a widely accepted risk factor for severe disease. It is possible this is due to misclassification or some mediation of factors included in the model?

Response 2.2: Thank you for the comment. The fact that immune status did not improve the model performance in the multivariable model might seem surprising at first sight, but it is in line with the hypothesis that the effect of a secondary immune response is actually mediated through other clinical covariates or predictors that were included in the model. Thus, in the univariable model, immune status is identified as a predictor, but is no longer visible when other covariates or predictors are included – these parameters likely represent mechanisms or pathways through which immune status manifests itself.

We have added an explanatory phrase in the Results:

> *"This result was unexpected and may indicate that any influence is mediated via clinical covariates or predictors already incorporated into the models." (lines 192-194)*

and expanded the Discussion:

> *"Secondary infection was identified as a risk factor for moderate/severe dengue in univariate analysis and the lack of improvement in model performance following inclusion of immune status merits careful consideration; one plausible explanation is that the influence of immune status is mediated via clinical covariates or predictors already incorporated into the models." (lines 298-302)*

Minor comments

1) While overall numbers of dengue cases and those analyzed match between the main text and supplement, it is still a bit confusing. It might be helpful to clarify by explicitly stating in the main text that 464 were excluded due to insufficient outcome data, with geogprahic breakdowns provided in the supplement.

Response 2.3: Thank you for the comment. We have modified the Results section to explicitly describe number of patients excluded due to insufficient outcome data.

> *"Within this group, 464/2694 (17%) patients were excluded as relevant outcomes could not be defined, while 2230/2694 (83%) individuals had sufficient data to define the main outcomes of interest and are included in our analysis datasets (Figure.1)." (lines 82-84)*

A detailed description of this group of patients, including a geographical breakdown, is provided in Table S8 in the Supplementary Information.

2) Excluded participants were largely DENV1. Given that Latin America was largely DENV-3 and Asia was dominated by DENV1&4, might this bias the comparison?

Response 2.4: Thank you for the comment. In our analysis, we compared values of readily available candidate predictors between groups of patients with and without moderate/severe dengue and developed prognostic models to predict moderate/severe dengue. As serotype data was only available in 89% of participants, we did not include this variable in the model development but only investigated its potential predictive value in the univariate and sensitivity analyses. It is correct that the excluded participants were largely infected with DENV-1, but this is mainly driven by the fact that 84% of the excluded participants were from Asia, consistent with the recruitment patterns in Asia versus Latin America. Thus the characteristics of patients who were included and excluded are quite similar, including continent of residence (84% Asia for the excluded group and 83% Asia for the included group) and infecting viral serotype (57% DENV-1 & 10% DENV-3 for the excluded group and 45% DENV-1 & 16% DENV-3 for the included group).

3) While the authors do acknowledge that hospitalization modifies disease course, it would be helpful to expand the discussion on how the dual role complicates interpretation of the models. Because hospitalization both reflects clincal judgement and alters disease progression through closer monitoring and care, the predictive models may be estimating risk under conditions that are not independent of the intervention. This needs to be clarified to strengthen the understanding of the limitations of conducting model-based vs clinician-based admission decision strategies.

Response 2.5: Thank you for raising this important point. We agree that our prediction models cannot be interpreted and applied outside of the clinical context, i.e. the current state of dengue case management. Given the current lack of effective early therapeutic options, there is no clear evidence that hospitalisation could have prevented progression to moderate dengue. However closer clinical monitoring and any associated intervention (e.g. parenteral fluid therapy) may well have prevented development of severe dengue. Indeed the small number of severe cases overall (38) may reflect this fact. The majority of these severe cases were identified at the moderate stage before progressing to severe, 13 on the same day and 13 within the following 1-2 days. In 12 cases an intermediate phase was not detected, suggesting very rapid progression in this tiny proportion of cases overall. It is important to highlight here that in the context of this observational study, all management decisions were left to the discretion of the treating clinicians - but we acknowledge that any close observational study with daily follow up like this could result in better clinical outcomes compared to settings without research, simply because of the attention and documentation involved. In our view, however, this makes the findings more relevant as we might have underestimated the true effect of the clinical signs and symptoms that were investigated.

In response, we have added to the Discussion an acknowledgement that our model's performance depends on the current clinical context and therefore will need to be updated or refined should the management landscape change.

> *"In addition, since prediction models cannot be interpreted and applied outside the context in which they are developed (in this case the current stage of dengue diagnosis and case management), our models will require updating and refinement should major changes to dengue case management become widely adopted or novel and effective intervention strategies become available." (lines 360-364)*

We also conducted an additional utility analysis restricted to moderate dengue only, to remove the potential protective effect of hospitalisation on severe dengue. Results from this analysis are very similar to our main analysis for moderate/severe dengue, which could be explained by the fact that most patients with the

moderate/severe dengue outcome were included in the model development when they developed moderate dengue (250/274, 91%). For this reason we have not included this additional analysis in the manuscript but would be happy to do so if this is considered helpful. We have expanded our Discussion on this issue in addition to our previous elaborations that our results may underestimate the net benefit of using our models for hospitalisation if a protective effect of hospitalisation exists:

> *"However, closer clinical monitoring and any associated interventions after admission may have prevented development of more severe disease that would otherwise have occurred. As this protective effect would primarily affect severe dengue rather than moderate dengue and most patients with moderate/severe dengue were included in our model development when they developed moderate dengue, its effect on our utility analysis would likely be small. In reality, the overall cost (in terms of 'unnecessary hospitalisations') of implementing our models as part of a hospitalisation algorithm would be lower – i.e. some proportion of the patients admitted in whom the outcome did not occur actually benefited from hospitalisation and might otherwise have progressed." (lines 366-374)*

REVIEWER #3

Summary of Key Results, Originality and Significance

The authors developed three prognostic models (Baseline, Dynamic-1, and Dynamic-2) using prospective data from over 2,200 confirmed dengue cases across 8 countries. These models predict moderate/severe dengue outcomes using clinical signs, symptoms, and hematological parameters. The dynamic models update predictions over the course of illness and show improved discrimination compared to clinicians' judgments and WHO warning sign-based strategies.

The manuscript is original in its use of dynamic modeling (landmark analysis and GEE) in a large multinational dengue cohort. Prior models have been largely cross-sectional, single-center, or lacked time-updated predictions. This study advances the methodological field and provides potentially valuable insights for triage in outpatient settings.

1. However, the novelty is limited if not coupled with a usable tool. Similar work with dynamic components and early prognosis has been published (e.g., Nguyen et al., CID 2017; Lam et al., PLOS NTD 2017). A clearer comparison with these studies is necessary to delineate the added value of this work.

Response 3.1: Thank you for acknowledging the originality of our manuscript. As the reviewer noted, our research group has previously published related work. However, Nguyen et al. (CID, 2017) focused only on a single prediction at enrolment in children, while Lam et al. (PLOS NTD, 2017) examined only the predictive value of repeated platelet counts. In contrast, the present study provides a comprehensive investigation of dynamic prediction using platelet count together with other readily available predictors of severe outcomes in both children and adults enrolled with suspected dengue at an early stage.

The previous work was mentioned in the Introduction section:

> *"Only three models have been developed for use in outpatient settings during the early febrile phase,28–30 and none can be updated to improve risk prediction as the illness episode evolves." (lines 52-54)*

We have revised the Results and Discussion sections to more clearly highlight the novelty of this work relative to our prior studies.

In the Results section

> *"3. Finally, we compared performance of our models with simpler models that rely on platelet count and/or WHO warning signs, in particular a model for severe dengue previously developed by our group for use in outpatient settings[29]. For this we fitted additional models that included only platelet count or*

warning signs, with and without illness day at enrolment and the interaction between age and continent. However, both of our dynamic models are clearly superior to platelet-only models and models that include only warning signs, in terms of discriminant ability (SupplementaryInformation_Figure.S2-PanelB)." (lines 209-215)

In the Discussion section

"The use of dynamic models that incorporate sequential daily information is especially relevant for dengue given the rapid clinical evolution. In an earlier study we demonstrated the utility of repeated platelet counts to update prediction for DSS.[39] In the current analysis we have incorporated a range of candidate predictors simultaneously (clinical as well as laboratory variables) and have shown clear superiority with respect to a repeated-values platelet-only model in terms of discriminant ability." (lines 303-308)

Data & Methodology

Strengths:

Robust, multicenter, prospective dataset with standard protocols. Appropriate use of logistic regression, landmark modeling, and GEE. Internal validation through bootstrapping is correctly applied.

Weaknesses:

2. Model development lacks transparency: Variable selection is guided by AIC and clinical judgment, but detailed selection criteria, handling of multicollinearity, and final model equations or coefficients are not presented.

Response 3.2: Thank you for these comments. In the previous version, we summarised only the key aspects of the model development procedure in the main text and provided a detailed description (including the variable selection procedure, handling of multicollinearity, and final model coefficients) in the Supplementary Information. As suggested by the reviewer, we have now moved the detailed information on the variable selection criteria (including both pre-selection and AIC-guided selection) and our approach to handling multicollinearity to the Methods section, and we have included the final model coefficients in Table 3 of the manuscript.

In the Method section

"From a total of 65 variables recorded in the CRF, we selected a pre-defined list of 29 candidate predictors that (1) were not part of the outcome definitions, (2) had <10% missing data at baseline, (3) occurred in 5-95% of participants (for clinical signs and symptoms) and were readily available in clinical practice in participating centres (for laboratory tests). The definition of each candidate predictor is provided in Table S3. To avoid multicollinearity, clinical signs/symptoms with strong correlations (overall agreement index > 0.7) were combined into single variables." (lines 599-604)

"The three full models were then simplified into final models using backward stepwise variable selection with the Akaike Information Criterion (for the final Baseline model) or a p-value of 0.15 (for the final Dynamic-1 and Dynamic-2 models) as the selection measure. Regression coefficients for the final models are provided in Table.3. The proof-of-concept web application for our models is provided at this address: https://lampk.shinyapps.io/IDAMSPRED/ (SupplementaryInformation _FigureS5)." (lines 704-710)

3. No data imputation: Missing data increases significantly after illness day 5. The decision not to impute is not sufficiently justified, and no sensitivity analysis is provided.

Response 3.3: As suggested by the reviewer, we have added a sensitivity analysis in which we used imputed datasets to explore the potential impact of missingness on our results. As shown in Supplementary Tables S10-12 and Supplementary Figure S1, we found no major differences in the model assumption assessments, estimated coefficients, or model performance, between the main and sensitivity analyses. We describe our

approach for the sensitivity analysis in the Methods (lines 770-776), summarise findings in the Results (lines 91-94), and provide detailed results in section B5 in the Supplementary Information (Supplementary Tables S10-12 and Supplementary Figure S1).

In the Results section

> *"Sensitivity analysis using simple imputation suggests that missing data had little impact on the model assumptions (SupplementaryTable S10&S11), estimated coefficients (SupplementaryTable S12), and model performance (SupplementaryFigure S1)." (lines 91-94)*

In the Methods section

> *" (8) Sensitivity analysis to explore potential impact of missing data: We used the mode (for categorical variables) and median (for continuous variables) to impute missing values of the candidate predictors at baseline, as well as the last observation carried forward approach to impute missing data from the sequential daily observations for each patient. Potential impacts of missing data on the model assumptions, estimated coefficients, and model performance were assessed by comparing results from the imputed dataset and the main analysis, and are presented in SupplementaryTables S10, S11, S12 and SupplementaryFigureS1." (lines 770-776)*

4. No external or temporal validation, despite recognized differences in model performance between Latin America and Asia.

Response 3.4: This is a unique prospective cohort with detailed daily measurements from a large number of patients enrolled in the early stage of dengue infection, and no comparable independent dataset is available for external validation. Given the limited number of events, we opted for resampling validation rather than a split-sample approach. We share the reviewer's concern regarding the generalizability of our models and therefore assessed this in several ways, including evaluating model performance in sub-populations (by continent and by age), in patients with suspected dengue infection, and by examining potential heterogeneities. We have modified the Methods section to include this statement:

> *"For validation, given the limited number of outcome events, we opted for resampling validation rather than a split-sample approach. No comparable independent dataset was available to allow external validation. We assessed generalisability by evaluating performance across subpopulations (by continent and age), in patients with suspected rather than confirmed dengue, and by examining potential heterogeneities." (lines 632-636)*

5. Supplementary information holds critical content (e.g., variable definitions, modeling steps) that should be incorporated into the main text.

Response 3.5: Thank you for this comment. As suggested, we have moved the detailed information on recruitment criteria and outcome definitions, as well as model development, which were previously described in the Supplementary Information, into the Methods section in the main text:

> *"Exclusion criteria included (1) having localising signs or symptoms suggesting an alternative diagnosis, or (2) being considered unlikely to attend daily follow-up (e.g. due to travelling distance from the clinic), as judged by the treating physician." (lines 578-581)*

> *"Moderate/severe dengue was defined as development of moderate/severe plasma leakage or moderate/severe bleeding or moderate/severe neurological involvement or moderate/severe liver involvement or moderate/severe involvement of another organ." (lines 594-596)*

> *"From a total of 65 variables recorded in the CRF, we selected a pre-defined list of 29 candidate predictors that (1) were not part of the outcome definitions, (2) had <10% missing data at baseline, (3) occurred in 5-95% of participants (for clinical signs and symptoms) and were readily available in clinical practice in participating centres (for laboratory tests). The definition of each candidate predictor is*

provided in Table.S3. To avoid multicollinearity, clinical signs/symptoms with strong correlations (overall agreement index > 0.7) were combined into single variables.*" (lines 599-604)*

"The three full models were then simplified into final models using backward stepwise variable selection with the Akaike Information Criterion (for the final Baseline model) or a p-value of 0.15 (for the final Dynamic-1 and Dynamic-2 models) as the selection measure. Regression coefficients for the final models are provided in Table.3. The proof-of-concept web application for our models is provided at this address: https://lampk.shinyapps.io/IDAMSPRED/ (SupplementaryInformation _FigureS5)." (lines 704-710)

In addition, we also modified Figure 1 and added Supplementary Table S4 to clarify different analysis populations used in our manuscript.

6. Provide complete final model equations and coefficients in the main text or supplementary materials.

Response 3.6: In the previous version, we provided the final model coefficients in the Supplementary Information. Because the models include many variables with non-linear transformations and interactions, we implemented risk calculation via a web application rather than presenting a complex equation; the link was removed during blinded review. In this revision, we have moved the model coefficients to Table 3, added instructions on how to derive risk predictions from these coefficients, and reinstated the web application link in both the Methods and Results sections.

In the Results section

"A demonstration of the web application for the three models developed can be accessed at this link: https://lampk.shinyapps.io/IDAMSPRED/ (SupplementaryInformation _FigureS5)." (lines 184-186)

Footnote of Table 3

"The predicted probability of moderate/severe dengue according to illness day (up to illness day 8) can be calculated using the following formula: Pr(moderate/severe dengue) = 1/(1 + exp(- linear predictor)), where the linear predictor is the product of the logistic regression coefficients provided in this table and the actual values of the predictors.." (lines 510-513)

In the Methods section

"Regression coefficients for the final models are provided in Table.3. The proof-of-concept web application for our models is provided at this address: https://lampk.shinyapps.io/IDAMSPRED/ (SupplementaryInformation _FigureS5)." (lines 707-710)

7. Add a parsimonious model (e.g., 4–6 variables) suitable for bedside use without digital support.

Response 3.7: We thank the reviewer and understand the rationale for this suggestion. However, in addition to developing the three main models we also examined several smaller models that include only well recognized predictors, such as the platelet count and other warning signs. The results indicate that these additional models performed worse than the main models. Given the complexity of outcome prediction in dengue, we feel that introducing another smaller model with an arbitrary set of predictors is unlikely to provide added value.

8. Conduct sensitivity analyses using imputed datasets or describe the pattern and impact of missingness.

Response 3.8: As noted in our earlier response (Response 3.3), we have added a sensitivity analysis using imputed datasets. The results showed no meaningful differences between the main and sensitivity analyses in model assumption checks, coefficient estimates, or model performance. We describe our approach for the sensitivity analysis in the Methods (lines 770-776), summarise findings in the Results (lines 91-94), and provide detailed results in section B5 in the Supplementary Information (Supplementary Tables S10-12 and Supplementary Figure S1).

9. Where external datasets are unavailable, consider temporal split-sample validation to better assess generalizability.

Response 3.9: As noted in our earlier response (Response 3.4), we used resampling rather than split-sample methods because of the limited number of outcome events. For generalizability, we assessed this aspect in several ways, including evaluating model performance in sub-populations (by continent and by age), in patients with suspected dengue infection, and by examining potential heterogeneities. We have modified the Methods section to include this explanation.

Use of Statistics and Uncertainty

The authors use appropriate performance metrics (AUC, calibration slope/in-the-large). Time-varying covariates and interactions are well modeled.

10. However, decision curve analysis (DCA) is not performed. Without this, the clinical net benefit of using the models remains speculative.

Response 3.10: Thank you for this helpful suggestion. We have now performed a decision curve analysis and include this analysis in our manuscript. We describe our approach for this analysis in the Methods section (lines 762-769), summarize the findings in the Results section (lines 236-240, 253-263), and have added the decision curve plot as panel C in figure 5.

In the Results section:

> *"In addition we carried out decision curve analysis[37] across a range of threshold probabilities to assess the net benefit of applying our models. We explored the net benefit of six different strategies, including hospitalisation for no-one, hospitalisation for all, hospitalisation based on the clinicians' decision alone, and hospitalisation based on different combinations of the clinicians' decision with our developed models (Figure 7-PanelC)." (lines 236-240)*
>
> *"From Figure 7-PanelC we see that hospitalisation strategies relying on clinicians' decisions, either alone or in combination with our models, would provide benefit at threshold probabilities <25% (i.e. if the cost ratio of admitting patients who do not progress to moderate/severe dengue versus missing patients who do progress is less than 1:3). However, all combination approaches provide greater benefit than clinicians' decisions alone, especially at threshold probabilities < 12% (i.e. for cost ratios less than 1:6.5) when the "clinician's decision alone" strategy may provide less benefit than the "hospitalisation for all" strategy. Despite negligible differences in net benefit between combination strategies, when the risk threshold is >30% (SupplementaryFigure_S6) the combined approach using the Dynamic Models results in fewer uncomplicated or non-severe dengue admissions for each patient who does subsequently develop the relevant outcome (moderate/severe or severe dengue, respectively), as compared to the Baseline Model combined approach." (lines 253-263)*

In the Methods section:

> *"Using the trade-off between the cost of admitting patients who never go on to develop the moderate/severe outcome and the cost of missing patients who do proceed to the moderate/severe outcome, we calculated the net benefit of hospitalisation. We compared the net benefit of six different strategies, including hospitalisation for no-one, hospitalisation for all, hospitalisation based on the clinicians' decision alone, and hospitalisation based on different combinations of the clinicians' decision with our developed models (Figure 7-Panel C, SupplementaryInformation _FigureS6)." (lines 762-769)*

11. The assumption that hospitalization does not influence disease progression is acknowledged but not formally tested or modeled.

Response 3.11: Since the reasons for hospitalization vary between hospitals and between clinicians, appropriately modeling or testing the impact of hospitalization using our data is not straightforward. Although

inverse probability weighting could potentially adjust for the confounding effect of hospitalization in causal analyses, the usefulness of this approach for evaluating the performance and utility of prediction models is unclear. Therefore, we chose to acknowledge and discuss this limitation in the Discussion section rather than performing formal testing.

To further explore this issue, we performed an additional utility analysis restricted to moderate dengue to minimise any potential protective effect of hospitalisation on progression to severe dengue. The results were very similar to those of our main moderate/severe dengue analysis, likely because nearly all patients with moderate/severe dengue first developed moderate dengue (250/274, 91%). We have expanded the Discussion accordingly, including our earlier point that if a protective effect of hospitalisation exists, our results may underestimate the net benefit of using the models to guide hospitalisation decisions.

> *"However, closer clinical monitoring and any associated interventions after admission may have prevented development of more severe disease that would otherwise have occurred. As this protective effect would primarily affect severe dengue rather than moderate dengue and most patients with moderate/severe dengue were included in our model development when they developed moderate dengue, its effect on our utility analysis would likely be small. In reality, the overall cost (in terms of 'unnecessary hospitalisations') of implementing our models as part of a hospitalisation algorithm would be lower – i.e. some proportion of the patients admitted in whom the outcome did not occur actually benefited from hospitalisation and might otherwise have progressed." (lines 366-374)*

12. Include DCA plots to quantify clinical utility at different decision thresholds.

Response 3.12: Thank you for this helpful suggestion. We have now performed a decision curve analysis and include this analysis in our manuscript. We describe our approach for this analysis in the Methods section (lines 762-769), summarize the findings in the Results section (lines 236-240, 253-263), and have added the decision curve plot as panel C in figure 5. We described details of these changes earlier under Response 3.10.

13. Discuss and, if feasible, model the potential impact of hospitalization on outcomes (e.g., using inverse probability weighting or sensitivity analysis).

Response 3.13: As described in Response 3.11, modelling or formally testing the impact of hospitalisation using our data is not straightforward and is beyond the scope of this manuscript. Although inverse probability weighting could, in principle, address confounding by hospitalisation in causal analyses, its relevance for evaluating the performance and clinical utility of prediction models is unclear. We therefore acknowledge this as a limitation in the Discussion rather than conducting formal tests. We have expanded the Discussion (lines 366-374) to note that any protective effect of hospitalisation would most likely influence progression to severe dengue rather than moderate dengue; and because nearly all patients with moderate/severe dengue first developed moderate dengue, the impact on our utility analysis is likely to be small.

Conclusions: Validity and Reliability

The conclusion that the models could enhance early triage and hospital admission decisions is supported in theory by the statistical analyses. However, these conclusions are not operationalizable due to:

14. The absence of a risk score or point-based algorithm.

Response 3.14: Thank you for this comment. However, risk score or point-based algorithms are generally considered less useful than web/computer-based applications, as they offer lower precision and are less practical for complex models (Moon KGM et al., 2015, http://dx.doi.org/10.7326/M14-0698). Therefore, we prefer to provide the detailed model coefficients along with the web application for prediction calculations. In this revision, we have moved the model coefficients to Table 3, added instructions on how to derive risk predictions from these coefficients, and reinstated the web application link in both the Methods and Results sections. We described details of these changes in Response 3.6.

15. No proposed thresholds for decision-making.

Response 3.15: Thank you for this comment. While data-driven risk thresholds could theoretically be established for our models, experts in prognostic model development have noted that such thresholds are rarely widely applicable. Thresholds are context-dependent and are generally best determined through a health economic analysis after a model has been externally validated (Wynants L et al., 2019, https://doi.org/10.1186/s12916-019-1425-3). Therefore, proposing a specific risk threshold for a specific use case would require further dedicated research with additional data on benefits and costs generated by different decisions. Within the scope of our study, our models provide continuous risk predictions, allowing for more nuanced, individualized decision-making and enabling the selection of different risk thresholds that can be tailored to specific clinical contexts.

In our revised Results section, the decision curve analysis suggests that combining clinicians' decisions with our models may provide greater net benefit than clinicians' decisions alone when the threshold probability is <25% (i.e., when the cost of admitting patients who do not progress to moderate/severe dengue, relative to missing those who do progress, is <1:3). In particular, at threshold probabilities <12% (cost ratio <1:6.5), the "clinicians' decision alone" strategy may yield less net benefit than a "hospitalise all" strategy. Although these threshold probabilities indicate scenarios in which our models could add value beyond clinicians' decisions alone, they should not be interpreted as recommended risk thresholds for decision-making.

In the Results:

> *"From Figure 7-PanelC we see that hospitalisation strategies relying on clinicians' decisions, either alone or in combination with our models, would provide benefit at threshold probabilities <25% (i.e. if the cost ratio of admitting patients who do not progress to moderate/severe dengue versus missing patients who do progress is less than 1:3). However, all combination approaches provide greater benefit than clinicians' decisions alone, especially at threshold probabilities < 12% (i.e. for cost ratios less than 1:6.5) when the "clinician's decision alone" strategy may provide less benefit than the "hospitalisation for all" strategy. Despite negligible differences in net benefit between combination strategies, when the risk threshold is >30% (SupplementaryFigure_S6) the combined approach using the Dynamic Models results in fewer uncomplicated or non-severe dengue admissions for each patient who does subsequently develop the relevant outcome (moderate/severe or severe dengue, respectively), as compared to the Baseline Model combined approach." (lines 253-263)*

16. Lack of external validation.

Response 3.16: As we mentioned in Response 3.9, this unique prospective cohort has no comparable external dataset and includes a limited number of events. Therefore, validation either using the split-sample strategy or obtaining a new independent dataset is impractical. We acknowledge this limitation and have modified the Methods section to include this justification.

17. No prototype of clinical integration (e.g., app, paper tool, EHR module).

Response 3.17: We had developed a Web application for risk calculation, but the link was unfortunately removed when we edited the manuscript for blinded review. In this revision, we have reinstated the web application link in both the Methods and Results sections. We described details of these changes in Response 3.6.

18. Thus, the models currently offer limited real-world applicability, and this limitation should be acknowledged more directly.

Response 3.18: Even though our models are not applicable for immediate use in the real world, our manuscript provides critical information on potential performance of the models, how to use them in practice, and summarises situations where these models could provide the most net benefit. We have revised the Discussion to highlight the need for further external validation and integration into locally relevant decision-support systems with appropriate risk thresholds.

"Although the effect of the selected predictors on the moderate/severe dengue outcome remained pretty robust across geographical locations and age-groups, local validation using an independent dataset and/or re-calibration of the various models should be considered.[47]" (lines 352-355)

"If validated and integrated into locally tailored decision-support systems with appropriate risk thresholds, these algorithms could provide major public health benefits in outbreak settings or in endemic areas with a high case burden." (lines 391-394)

Suggested Improvements

To justify publication and maximize the study's impact, the following improvements are essential:

Transparency:

19. Publish full model coefficients, preferably as a point-based scoring system.

Response 3.19: In this revision, we have moved the model coefficients to Table 3, added instructions on how to derive risk predictions from these coefficients, and reinstated the web application link in both the Methods and Results sections. We described details of these changes in Response 3.6. We also provided justification for using the Web application instead of a point-based scoring system in Response 3.14.

20. Clarify the selection process for predictors, with justification for inclusion/exclusion.

Response 3.20: We have moved detailed information on the recruitment criteria, outcome definitions, and model development (including variable selection), previously described in the Supplementary Information, to the Methods section in the main text. We described the details of these changes earlier under Response 3.2 and Response 3.5.

Clinical Translation:

21. Develop and present a simplified risk score with a manageable number of variables.

Response 3.21: We had developed a Web application, which can be used to calculate the predicted risk quickly via mobile phone or tablet, and have now reinstated the web application link in both the Methods and Results sections. Details of these changes are provided under Response 3.6, and our rationale for not developing a more parsimonious model is discussed in Response 3.7.

22. Provide a link, screenshot, or usability description of the mentioned web tool.

Response 3.22: We have reinstated the web application link in both the Methods and Results sections (details of these changes are provided in Response 3.6). We have provided a screenshot of this application as Figure S5 in the Supplementary Information.

23. Suggest risk thresholds for triage, even as illustrative examples.

Response 3.23: In our revised results, the decision curve analysis suggests that combining clinicians' decisions with our models may provide greater net benefit than clinicians' decisions alone when the threshold probability is <25% (i.e., when the cost of admitting patients who do not progress to moderate/severe dengue, relative to missing those who do progress, is <1:3). In particular, at threshold probabilities <12% (cost ratio <1:6.5), the "clinicians' decision alone" strategy may yield less net benefit than a "hospitalise all" strategy. Although these threshold probabilities indicate scenarios in which our models could add value beyond clinicians' decisions alone, they should not be interpreted as recommended risk thresholds for decision-making. We provide our reasons for not recommending risk thresholds derived from this study in Response 3.15.

Validation and Implementation:

24. Conduct temporal or geographic cross-validation within the current data.

Response 3.24: We conducted a geographic cross-validation type analysis by examining potential heterogeneities and evaluating model performance in subpopulations (by continent and by age) among patients with suspected dengue infection. These results are presented in the Results section (lines 114-118 and 195-208). We did not perform temporal or external validation due to the small number of outcome events in our study and the lack of a suitable external dataset. We have described this rationale in more detail under Response 3.4 and added this explanation to the Methods section accordingly.

25. Discuss feasibility of implementation in low-resource settings.

Response 3.25: Thank you for raising this point. The web application is designed for use on a mobile phone. We have expanded the Discussion section to cover implementation in low resource settings and to include more detail on local calibration.

> *"Although the effect of the selected predictors on the moderate/severe dengue outcome remained pretty robust across geographical locations and age-groups, local validation using an independent dataset and/or re-calibration of the various models should be considered.[47] When miscalibration is evident using local data, the various models can be updated by adjusting the regression coefficients.[48]" (lines 352-356)*

> *"In addition, our utility analysis relies on the assumption that our models are perfectly implemented. Although implementation in low-resource clinical settings could be challenging, the fact that our models only require readily available clinical and laboratory variables should minimise such difficulties. Given the ever-increasing mobile phone coverage globally, integrating our models into a smartphone web application could also facilitate their use in clinical settings. Finally, in tertiary hospitals with electronic data management systems, these models could be integrated into patient monitoring devices that retrieve input automatically from real-time databases." (lines 374-381)*

26. Address explicitly the lack of external validation and how the tool might be calibrated locally.

Response 3.26: We have modified the Discussion to emphasize the need for external validation and to provide suggestions for calibrating our models locally:

> *"Although the effect of the selected predictors on the moderate/severe dengue outcome remained pretty robust across geographical locations and age-groups, local validation using an independent dataset and/or re-calibration of the various models should be considered.[47] When miscalibration is evident using local data, the various models can be updated by adjusting the regression coefficients.[48]" (lines 352-356)*

We have also modified the Methods to include the rationale for not using a split-sample approach to validation (as discussed in more detail under Response 3.4).

> *"For validation, given the limited number of outcome events, we opted for resampling validation rather than a split-sample approach. No comparable independent dataset was available to allow external validation. We assessed generalisability by evaluating performance across subpopulations (by continent and age), in patients with suspected rather than confirmed dengue, and by examining potential heterogeneities." (lines 632-636)*

Clarity and Accessibility:

27. Move critical elements (model structure, assumptions, predictor definitions) from Supplement to the main text.

Response 3.27: As mentioned in Response 3.20, we have moved detailed information on the recruitment criteria, outcome definitions, and model development (including variable selection), previously described in the Supplementary Information, to the Methods section in the main text. We also modified Figure 1 and added

Supplementary Table S4 to clarify different analysis populations used in our manuscript. We described the details of these changes earlier under Response 3.2 and Response 3.5.

28. Improve figure legends for accessibility, especially in Figures 4 and 5.

Response 3.28: Thank you for the comment. Accordingly, we have modified the legends for Figure 6 (previously Figure 4) and Figure 7 (previously Figure 5) to improve accessibility:

Legend for Figure 6 (previously Figure 4):

> *"Optimism-corrected AUC (top row), calibration in-the-large (middle row) and calibration slope (bottom row) for the three models, evaluated at baseline and up to 3 days after study enrolment across study subgroups (Panel A) and by illness day (Panel B). Points denote medians and vertical lines the 2.5th–97.5th percentiles from 1,000 bootstrap replicates used to estimate optimism. Numbers below each plot indicate the number of participants used for model development on that day and, in bold, the number who subsequently developed moderate/severe dengue. The Dynamic-2 model could not be evaluated at baseline because it includes day-to-day change predictors. For visual clarity, values were truncated as follows: AUC 0.5–1.0; calibration-in-the-large −1 to 1; calibration slope −1 to 2.*
>
> *Note: AUC quantifies discrimination (i.e. the probability that a case has higher predicted risk than a non-case; ideal = 1). Calibration reflects the level of agreement between the model predictions and the observed outcomes; calibration-in-the-large assesses systematic over/underprediction (ideal = 0) while the calibration slope reflects the average effect of the predictors on the outcome (ideal = 1)." (lines 519-531)*

Legend for Figure 7 (previously Figure 5):

> *"Panels A-B: Sensitivity and specificity for hospitalizing patients at least one day before outcome onset (A: moderate/severe dengue, B: severe dengue), using different candidate models and decision rules. In addition to our models, we assessed rule-based strategies based on the number of warning signs (at least 1, 2, or 3 warning signs present up to the day of assessment, with warning signs being persistent vomiting, significant abdominal pain/tenderness, lethargy/restlessness, mucosal bleeding, platelet count ≤ 50K/mm^3), and clinician-initiated admission for any medical reason (clinician's decision). Models and rules were assessed alone and in combination with the clinician's decision. The three models (Baseline, Dynamic-1 and Dynamic-2) were evaluated at different risk thresholds (i.e. cut-offs at which a responsible clinician might consider the predicted risk sufficient to warrant admission) and are presented as solid lines for model-only strategies, and dashed lines for strategies combining the clinician's decision with one of the three models. Sensitivities, specificities were calculated over the illness course for all participants.*
>
> *Panel C: Decision curve analysis for various combinations of the clinician's decision with our developed models to hospitalise patients with dengue.*
>
> *For any given threshold probability, the clinical utility of different strategies to hospitalise patients with dengue can be measured in terms of "net benefit" - i.e. the number of pre-emptively hospitalised patients who progress to moderate/severe dengue, after discounting for the number of hospitalised patients who do not develop the moderate/severe outcome. The threshold probability reflects the ratio between the cost of admitting a patient who does not develop the moderate/severe outcome and the cost of missing a patient who does develop the moderate/severe outcome; for example, a threshold probability of 10% refers to a ratio of 1:9.*
>
> *This threshold can also be used to define the risk threshold to hospitalise using prediction models. The figure compares net benefits for six hospitalisation strategies: hospitalisation for no-one (grey dashed line), hospitalisation for all (grey line), hospitalisation based on the clinician's decision alone (black line), and hospitalisation based on a combination of the clinician's decision plus one of the three developed*

models (Baseline, Dynamic-1, Dynamic-2 shown in red, blue and green respectively). This graph shows that hospitalisation based on the clinicians' decisions alone could provide higher net benefit compared to the "hospitalisation for no-one" and "hospitalisation for all" strategies for threshold probabilities between 12% and 17%. Combining clinicians' decisions with these models to hospitalise patients with dengue could provide greater benefit than all other strategies for threshold probabilities between 1% and 22%." (lines 538-569)

References

29. References are generally appropriate, with inclusion of recent systematic reviews and WHO guidance. However, there is insufficient discussion of related dynamic or outpatient-focused models. Relevant works, such as Nguyen et al. (CID 2017), Tanner et al. (PLoS NTD 2008), and Lam et al. (PLOS NTD 2017), should be cited and compared more explicitly to highlight advancements or limitations.

Response 3.29: All three citations are already included in the manuscript and actually two of them are from our own group. We cited them in the Introduction, conducted exploratory analyses comparing our results to the Nguyen CID 2017 model, and discussed Lam PLOS NTD 2017 paper in the Discussion. We have revised the Results and Discussion to make these comparisons and discussions clearer for the reader. We described details of these changes in Response 3.1.

EDITOR

1. Please add data to the Abstract, per Reviewer #1, keeping to our 200-word limit. Additionally, please include concise definitions of moderate and severe dengue, along with the study's inclusion and exclusion criteria, directly in the main text rather than referring readers to the Supplementary Materials.

Response 4.1: We have revised the Abstract to add data within the 200-word limit to address request from Reviewer 1 (Response 1.1). We also revised the Methods section to include definitions of moderate/severe dengue and the study's inclusion and exclusion criteria.

Revised text in the Methods:

> *"Study staff enrolled participants aged 5 years or more attending clinics in 8 countries across Asia and Latin America (LA) with symptoms consistent with possible dengue (suspected dengue and/or undifferentiated fever in a patient from a dengue-endemic area) and fever for less than 3.5 days, provided they were presently suitable for outpatient management. We obtained ethical approvals from the relevant institutional and national boards for each participating centre, and all patients or a responsible parent/guardian gave written informed consent. Exclusion criteria included (1) having localising signs or symptoms suggesting an alternative diagnosis, or (2) being considered unlikely to attend daily follow-up (e.g. due to travelling distance from the clinic), as judged by the treating physician." (lines 573-581)*

> *"Moderate/severe dengue was defined as development of moderate/severe plasma leakage or moderate/severe bleeding or moderate/severe neurological involvement or moderate/severe liver involvement or moderate/severe involvement of another organ. For each component included within this diagnostic category we applied definitions consistent with the consensus on standard clinical endpoints for dengue intervention trials (SupplementaryInformation_A.6, Table.S2).[33]" (lines 594-598)*

2. Finally, please expand the section on ethical approvals to include institution specific details, and provide information on how to access the data supporting the study's findings in the Data Availability statement.

Response 4.2: We have revised the Ethical declarations section to include an Ethics Committee approval subsection, detailing the issuing institution, approval type, and approval date (lines 811-813, pages 56-60). We have also updated the Data availability statement to describe the current procedure for accessing the data supporting our findings (lines 778-783, page 56).

Ethics Committee approval section:

> *"We obtained ethical approvals from the Oxford Tropical Research Ethics Committee and all relevant institutional and national boards for each participating centre. All patients or a responsible parent/guardian gave written informed consent." (lines 812-813)*
>
> *(We added a table described name of the issuing institution, approval type, and approval date in pages 56-60))*

Data availability section:

> *"Data will be deposited at the European Genome-phenome Archive (EGA), which is hosted by the European Molecular Biology Laboratory-European Bioinformatics Institute and the Centre for Genomic Regulation. The accession number will be communicated in due course. In the meantime, the corresponding authors will act as contact points for data requests. Further information about EGA can be found at https://ega-archive.org and The European Genome-phenome Archive in 2021: 10.1093/nar/gkab1059" (lines 779-783)*
