# Supplementary material for: Improving early prediction for adverse dengue outcomes using data from the IDAMS prospective multicentre study
Source: Nat Commun. Author manuscript; Available in PMC 2026 Sep 26. (PMC13590484; doi:10.1038/s41467-026-76450-2)
Supplement: Supplementary file 2 [file EMS218154-supplement-Reporting_Summary.pdf]

nature portfolio

Corresponding author(s): Phung Khanh Lam, Bridget Wills

Last updated by author(s): Jul 1, 2026

Reporting Summary

Nature Portfolio wishes to improve the reproducibility of the work that we publish. This form provides structure for consistency and transparency in reporting. For further information on Nature Portfolio policies, see our Editorial Policies and the Editorial Policy Checklist.

Statistics

For all statistical analyses, confirm that the following items are present in the figure legend, table legend, main text, or Methods section.

n/a	Confirmed	
☐	☒	The exact sample size (n) for each experimental group/condition, given as a discrete number and unit of measurement
☐	☒	A statement on whether measurements were taken from distinct samples or whether the same sample was measured repeatedly
☐	☒	The statistical test(s) used AND whether they are one- or two-sided<br>*Only common tests should be described solely by name; describe more complex techniques in the Methods section.*
☐	☒	A description of all covariates tested
☐	☒	A description of any assumptions or corrections, such as tests of normality and adjustment for multiple comparisons
☐	☒	A full description of the statistical parameters including central tendency (e.g. means) or other basic estimates (e.g. regression coefficient) AND variation (e.g. standard deviation) or associated estimates of uncertainty (e.g. confidence intervals)
☐	☒	For null hypothesis testing, the test statistic (e.g. F, t, r) with confidence intervals, effect sizes, degrees of freedom and P value noted<br>*Give P values as exact values whenever suitable.*
☒	☐	For Bayesian analysis, information on the choice of priors and Markov chain Monte Carlo settings
☒	☐	For hierarchical and complex designs, identification of the appropriate level for tests and full reporting of outcomes
☒	☐	Estimates of effect sizes (e.g. Cohen's d, Pearson's r), indicating how they were calculated

Our web collection on statistics for biologists contains articles on many of the points above.

Software and code

Policy information about availability of computer code

Data collection	Data were collected on a standardized paper case report form and then entered into a Microsoft Access database using a customized data entry software tool
Data analysis	All analyses were performed using R version 4.0.4 and its companion packages. All codes for analyses will be made available to editors and reviewers at this GitHub repository: https://github.com/lampk/IDAMS_prediction

For manuscripts utilizing custom algorithms or software that are central to the research but not yet described in published literature, software must be made available to editors and reviewers. We strongly encourage code deposition in a community repository (e.g. GitHub). See the Nature Portfolio guidelines for submitting code & software for further information.

Data

Policy information about availability of data

All manuscripts must include a data availability statement. This statement should provide the following information, where applicable:

- Accession codes, unique identifiers, or web links for publicly available datasets
- A description of any restrictions on data availability
- For clinical datasets or third party data, please ensure that the statement adheres to our policy

Data will be deposited at the European Genome-phenome Archive (EGA), which is hosted by the European Molecular Biology Laboratory-European Bioinformatics Institute and the Centre for Genomic Regulation. Public deposition of the full dataset is in progress and data from sites with fully executed Data Transfer Agreements are being uploaded on a rolling basis, with the complete dataset anticipated to be available within the coming months. In the interim, the IDAMS data

are available under restricted access, as the original informed consent did not include explicit language for public data sharing, necessitating additional ethics committee approvals and legal agreements across multiple participating sites. Reasonable requests to access the dataset for research aligned with the scope of the initial study (e.g., dengue or other arbovirus research) will be considered by the Data Access Committee at the Oxford University Clinical Research Unit in Ho Chi Minh City (https://www.oucru.org/data-sharing-policy/; dac@oucru.org). Additional source data for data-dependent figures are provided with this paper in the Source Data file.

Research involving human participants, their data, or biological material

Policy information about studies with human participants or human data. See also policy information about sex, gender (identity/presentation), and sexual orientation and race, ethnicity and racism.

Reporting on sex and gender	All individuals who met the enrolment criteria were included, regardless of sex and gender. Information on the sex of participants (self-reported) was recorded by the study team. We describe the frequency of sex, overall and by continent in Table 1. We also considered sex as a candidate predictor in our models and present univariate and multivariable analyses of relationships between sex and other candidate predictors with clinical outcome in Table 2. No other formal analysis specifically relating to sex was planned or conducted.
Reporting on race, ethnicity, or other socially relevant groupings	We collected and reported information on study sites, country and continent of participants, but not on race/ethnicity. Information on study sites, country and continent are reported in Table 1, Figure 2. Due to potential differences between Asia and Latin America regarding background transmission and immunity for dengue, most analyses were reported for Asia and Latin America separately.
Population characteristics	A total of 2,230 patients with laboratory-confirmed dengue infection were enrolled in the study, of whom 83% were recruited from sites in Asia. The median age of participants was 14 years (interquartile range, 9 to 24), and 52% were children younger than 15 years. In Latin American sites, most enrolled participants were children under 15 years of age (85%), whereas in Asia, only 45% were children, reflecting differences in the populations served by the respective study sites. Females were underrepresented in the overall study population, accounting for 42% of participants, and this pattern was observed across both continents. Most participants were enrolled on illness days 2 or 3, although enrolment occurred slightly later in Latin America than in Asia. Consistent with this, the proportion of patients with viremia below the detection limit at enrolment was higher in Latin America. DENV-1 and DENV-4 were the predominant serotypes in Asia, whereas DENV-3 was more common in Latin America. In Asia, most patients had probable secondary infections. In contrast, only 27% of patients in Latin America had probable secondary infections, while immune status was inconclusive in 40%.
Recruitment	Participants were recruited from 24 outpatient health facilities in 8 countries across Asia and Latin America. Adults and children (at least 5 years) presenting to study sites with febrile illness consistent with dengue and within the first 3.5 days of fever onset were enrolled, after appropriate informed consent. Written informed consent was obtained from all patients or from the parent/guardian of children.<br>There is no potential for self-selection bias in this study. However, the fact that hospitalisation may change the illness course could affect our assessment of the models' utility with respect to pre-emptive admission of patients who later progress to more severe disease.
Ethics oversight	We obtained ethical approvals from the Oxford Tropical Research Ethics Committee and all relevant institutional and national boards for each participating centre. Names of the institute/organization whose ethical committee approved the protocol, as well as the names of the Ethics committees include: Ethics Committee (Angkor Hospital for Children, Siem Reap, Cambodia), Medical and Health Research Ethics Committee (Faculty of Medicine, Gadjah Mada University, Indonesia), Medical Ethics Committee (University of Malaya Medical Centre, Malaysia), Ethics Committee (Clinical Research Center, National Institutes of Health, Ministry of Health, Malaysia), Medical Research & Ethics Committee (Ministry of Health, Malaysia), Ethics Committee (Children's Hospital 2, Ho Chi Minh City, Vietnam), Ethics Committee (Hospital for Tropical Diseases, Ho Chi Minh City, Vietnam), Ethics Committee (National Hospital for Tropical Diseases, Ha Noi, Vietnam), Ethics Committee (ICDDR-B, Dhaka, Bangladesh), Comite de Etica em Pesquisa (Fundacao Universidade Estadual do Ceara, Fortaleza), Comissao Nacional de Éthica em Pesquisa (Ministry of Health, Brazil), Comitê de Ética em Pesquisa em Seres Humanos (Instituto de Medicina Integral Prof. Fernando Figueira, Recife, Brazil), Comitê de Ética em Pesquisa (Centro de Pesquisas Aggeu Magalhaes, Recife, Brazil), Comitê de Ética em Pesquisa (Instituto de Pesquisa Clinica Evandro Chagas, Rio de Janeiro, Brazil), Comité de Ética en Investigación Clínica (Hospital Nacional de Ninos Benjamin Bloom, San Salvador), Fundación Carabobeno para la Salud, Valencia, Venezuela; Instituto de Investigaciones Biomedicas, Maracay, Venezuela.<br><br>All patients or a responsible parent/guardian gave written informed consent after discussion with study staff. Children aged 12–17 also provided assent using a separate form after discussion with study staff. For illiterate participants the consent or assent form was read aloud in the presence of a witness. All study documents were provided in the local language using easily understandable terms.

Note that full information on the approval of the study protocol must also be provided in the manuscript.

Field-specific reporting

Please select the one below that is the best fit for your research. If you are not sure, read the appropriate sections before making your selection.

☒ Life sciences ☐ Behavioural & social sciences ☐ Ecological, evolutionary & environmental sciences

For a reference copy of the document with all sections, see nature.com/documents/nr-reporting-summary-flat.pdf

Life sciences study design

All studies must disclose on these points even when the disclosure is negative.

Sample size	The estimated sample size was 7-8,000 cases based on assumptions regarding prevalence of lab-confirmed dengue (50% in Asia, 20-30% in Latin America), prevalence of negative diagnosis (80%), proportion of cases progressing to severe dengue (5%), and expected number of patients progressing to severe dengue (100-150 patients).
Data exclusions	No data was excluded. Patients with lab-confirmed dengue and sufficient data to define outcome were included in the main analysis. However, all participants were included in the additional analysis to explore the performance of the models on a population with suspected dengue.
Replication	Double data entry was used to limit data entry errors. Variable derivations were coded and cross-checked by team members with experience in programming and data analysis. Derivation of individual outcomes were based on a computer algorithm replicating outcome definitions and these were verified by an endpoint review committee.
Randomization	This is an observational study and no randomization was required.
Blinding	Assessment of candidate predictors was completed without knowledge of the patients' final outcomes. Individual clinical outcomes were derived after the data collection was finished, based on a computer algorithm replicating outcome definitions that were verified by an endpoint review committee.

Reporting for specific materials, systems and methods

We require information from authors about some types of materials, experimental systems and methods used in many studies. Here, indicate whether each material, system or method listed is relevant to your study. If you are not sure if a list item applies to your research, read the appropriate section before selecting a response.

Materials & experimental systems

n/a	Involved in the study	
☒	☐	Antibodies
☒	☐	Eukaryotic cell lines
☒	☐	Palaeontology and archaeology
☒	☐	Animals and other organisms
☐	☒	Clinical data
☒	☐	Dual use research of concern
☒	☐	Plants

Methods

n/a	Involved in the study	
☒	☐	ChIP-seq
☒	☐	Flow cytometry
☒	☐	MRI-based neuroimaging

Clinical data

Policy information about clinical studies

All manuscripts should comply with the ICMJE guidelines for publication of clinical research and a completed CONSORT checklist must be included with all submissions.

Clinical trial registration	NCT01550016 (https://clinicaltrials.gov/study/NCT01550016)
Study protocol	https://bmcinfectdis.biomedcentral.com/articles/10.1186/s12879-016-1440-3
Data collection	This prospective, multi-centre observational study involved 7,428 participants aged 5 years or more who presented with febrile illness suggestive of dengue at outpatient health facilities in eight countries across Asia and Latin America. Individuals were eligible if they presented within 3.5 days of fever onset and showed no signs of severe disease. We used a standardized case report form to collect a broad range of clinical and laboratory data at enrolment, daily for up to six days during the acute phase of illness, as well as at a follow-up visit one week later. Routine laboratory tests and dengue diagnostic samples were collected at specified time points, and final diagnoses and immune status were determined using defined algorithms.
Outcomes	Our main clinical outcome is moderate/severe dengue, defined as development of moderate/severe plasma leakage or moderate/severe bleeding or moderate/severe neurological involvement or moderate/severe liver involvement or moderate/severe involvement of another organ. These outcomes were defined using definitions consistent with the consensus on standard clinical endpoints for dengue intervention trials. We provide detailed definition of these outcomes in section 6 of the Supplementary Methods. To derive and verify individual clinical outcomes, we developed a computer algorithm based on these criteria and definitions and used it to automatically classify the clinical outcome for each patient. For all those who met the criteria for moderate/severe dengue, an endpoint review committee carefully checked the information in the CRF and, where necessary, verified the final category using additional information from hospital records and/or personal communication with local researchers.

Plants

Seed stocks

Report on the source of all seed stocks or other plant material used. If applicable, state the seed stock centre and catalogue number. If plant specimens were collected from the field, describe the collection location, date and sampling procedures.

Novel plant genotypes

Describe the methods by which all novel plant genotypes were produced. This includes those generated by transgenic approaches, gene editing, chemical/radiation-based mutagenesis and hybridization. For transgenic lines, describe the transformation method, the number of independent lines analyzed and the generation upon which experiments were performed. For gene-edited lines, describe the editor used, the endogenous sequence targeted for editing, the targeting guide RNA sequence (if applicable) and how the editor was applied.

Authentication

Describe any authentication procedures for each seed stock used or novel genotype generated. Describe any experiments used to assess the effect of a mutation and, where applicable, how potential secondary effects (e.g. second site T-DNA insertions, mosiacism, off-target gene editing) were examined.
