# Supplementary material for: Improving early prediction for adverse dengue outcomes using data from the IDAMS prospective multicentre study
Source: Nat Commun. Author manuscript; Available in PMC 2026 Sep 26. (PMC13590484; doi:10.1038/s41467-026-76450-2)
Supplement: Supplementary file 3 [file EMS218154-supplement-Supplementary_Document.pdf]

IMPROVING EARLY PREDICTION FOR ADVERSE DENGUE OUTCOMES USING DATA FROM THE IDAMS PROSPECTIVE MULTICENTRE STUDY

TABLE OF CONTENTS

LIST OF ALL MEMBERS OF THE INTERNATIONAL RESEARCH CONSORTIUM ON DENGUE RISK ASSESSMENT, MANAGEMENT, AND SURVEILLANCE CLINICAL INVESTIGATORS GROUP......4

SUPPLEMENTARY METHODS: STUDY PROCEDURES AND DEFINITIONS......5

1. Administration......5

2. Study population and participant enrolment......5

3. Important study definitions......5

Supplementary Table 1: Study definitions for time-dependent events......6

4. Clinical and laboratory assessments and clinical management......6

5. Dengue diagnostic testing and definitions......7

6. Definitions for clinical outcomes......8

Supplementary Table 2: Working definitions for all components of the clinical outcome......9

7. Candidate predictors......10

Supplementary Table 3: Definitions for candidate predictors......12

SUPPLEMENTARY NOTES......14

1. Missing data......14

2. Heterogeneity assessment......15

Supplementary Table 4: Summary of the different analysis populations.......16

Supplementary Table 5: Summary of linearity assessment (n = 2130).......17

Supplementary Table 6. Summary of interaction assessment (n = 2130)..18

Supplementary Table 7: Optimism-corrected AUC of models with and without interaction assessment in the development procedure (n = 2130). 19

Supplementary Table 8: Enrolment characteristics of participants with confirmed dengue who were excluded from or included in the analysis population (n = 2694). 20

Supplementary Table 9: Description of missing data for the analysis population (n = 2230)...........22

Supplementary Table 10: Summary of linearity assessment (n = 2206, imputed data).....................24

Supplementary Table 11: Summary of interaction assessment (n = 2206, imputed data). 25

Supplementary Table 12: Regression coefficients of models using complete case & imputed data. .26

Supplementary Figure 1: Apparent performance of the three models, evaluated at baseline (study day 1) and up to 3 days after study enrolment in different subsets of the study population in the imputed dataset (n = 2206)..28

Supplementary Table 13: Characteristics, linear predictors and membership c-statistics for each study site (n = 2206)..29

Supplementary Table 14: Prediction performance a) separately for each site with at least 10 events of moderate/severe dengue, and b) grouped by continent, based on a baseline prediction model that was developed on all other sites or the other continent..31

Supplementary Figure 2: Optimism-corrected AUCs of additional exploratory models applied on each of the first 3 study days..32

Supplementary Figure 3: Study flowchart to describe sample sizes for analyses of suspected dengue (results presented in Supplementary Fig. 4 and Supplementary Tables 15-18). 33

Supplementary Figure 4: Optimism-corrected AUCs of models applied to the confirmed dengue (n = 2130) and suspected dengue populations (n = 5290) on each of the first 3 study days.....................34

Supplementary Table 15: Distribution of candidate predictors at enrolment in the lab-confirmed dengue (n = 2206) and suspected-dengue populations (n = 5520), separately for Latin America and Asia..35

Supplementary Table 16. Univariable relationships between each candidate predictor and outcome in the lab-confirmed dengue and suspected-dengue populations in Latin America. 36

Supplementary Table 17: Univariable relationships between each candidate predictor and outcome in the lab-confirmed dengue and suspected-dengue populations in Asian children. 37

Supplementary Table 18: Univariable relationships between each candidate predictor and outcome in the lab-confirmed dengue and suspected-dengue populations in Asian adults. 38

Supplementary Figure 5: A demonstration of the web application for the three models developed.39

(URL: https://lampk.shinyapps.io/IDAMSPRED/) 39

Supplementary Figure 6: Number of cases with uncomplicated dengue (neither moderate nor severe) (Panel A) or non-severe dengue (Panel B) hospitalised for each patient pre-emptively admitted prior to development of the relevant outcome, at different risk thresholds. 40

Supplementary Figure 7: Distributions of the predicted risks derived from the Dynamic-1 and Dynamic-2 models on the day of hospitalisation, for patients who were hospitalised for medical reasons, stratified by study site (n = 2130). 41

3. TRIPOD Checklist: Prediction Model Development 42

SUPPLEMENTARY REFERENCES 44

List of all members of the International Research Consortium on Dengue Risk Assessment, Management, and Surveillance Clinical Investigators Group

Bangladesh, Dhaka: Firoz Ahmed (FA), Md Zabir Hasan (MZH), Malabika Sarker (MS)

Brazil, Fortaleza: Bruno Souza Benevides (BSB), Andrea Caprara (AC), Kilma Wanderley Lopes Gomes (KWLG), Lyvia Patricia Soares Mesquita (LPSM)

Brazil, Recife, Pernambuco: Tereza Magalhaes (TM), Ernesto T. A. Marques (ETAM)

Brazil, Rio de Janeiro: Patricia Brasil (PB), Guilherme Calvet (GC), Marco Netto (MN)

Cambodia, Siem Reap: Ngoun Chanpheaktra (NC), Bophal Chuop (BC), Varun Kumar (VK), Sonyrath Ouk (SO), Sopheary Sun (SS)

Cuba, Havana: Mayling Alvarez (MA), Maria Guzman (MG)

El Salvador, San Salvador: Guillermo Edgardo Barahona (GEB), Bladimir Rodolfo Cruz Peña (BRCP), Gabriela Maria Marón (GMM), Ernesto Pleités Sandoval (EPS)

Germany, Heidelberg: Thomas Jaenisch (TJ), Kerstin Daniela Rosenberger (KDR)

Indonesia, Yogyakarta: Eggi Arguni (EA), Rizka Humardewayanti (RH), Ratni Indrawanti (RI), Ida Safitri Laksanawati (ISL), Yodi Mahendradhata (YM)

Malaysia, Kuala Lumpur: Juraina Abd-Jamil (JAJ), Sazaly AbuBakar (SAB), Edmund Prakash Das (EPD), Bee Kiau Ho (BKH), Santha Kumari Natkunam (SKN), Sharifah Faridah Syed Omar (SFSO), Jameela Sathar (JS), Lucy Lum Chai See (LLCS)

Venezuela, Valencia: Sarah E. Bethencourt (SEB), Erley Ferlipe Lizarazo (EFL), Adriana Tami (AT), Robert Tovar (RT), María Fernanda Vincenti-González (MFVG)

Viet Nam, Ho Chi Minh City: Cao Thi Tam (CTT), Dinh Thi Tri Hong (DTTrH), Do Chau Viet (DCV), Dong Thi Hoai Tam (DTHT), Ronald B. Geskus (RBG), Huynh Lam Thuy Trinh (HLTT), Huynh Le Anh Huy (HLAH), Huynh Thi Le Duyen (HTLD), Lai Thi Cong Thanh (LTCT), Nguyen Tan Thanh Kieu (NTTK), Nguyen Than Ha Quyen (NTHQ), Nguyen Thi Hong Van (NTHV), Nguyen Thi Thu Hiep (NTTH), Nguyen Tran Nam (NTN), Phung Khanh Lam (PKL), Cameron Simmons (CS), Tran Thi Kim Van (TTKV), Tran Thi Nhu Thuy (TTNT), Tran Van Ngoc (TVN), Truong Thi Thu Thuy (TTTT), Vo Thanh Luan (VTL), Bridget Wills (BW), Marcel Wolbers (MW)

Viet Nam, Hanoi: Dang Thi Bich (DTB), Dinh Thi Thu Huong (DTTH), Dinh Van Huy (DVH), Nguyen Nguyen Huyen (NNH), Nguyen Van Kinh (NVK), Vu Thi Thu Huong (VTTH), Sophie Yacoub (SY)

SUPPLEMENTARY METHODS: Study procedures and definitions

1. Administration

The study was coordinated centrally from the Section Clinical Tropical Medicine, Heidelberg University Hospital, Heidelberg, Germany, and the Oxford University Clinical Research Unit (OUCRU) in Ho Chi Minh City (HCMC), Vietnam. The study is registered with ClinicalTrials.gov, registration number NCT01550016.

2. Study population and participant enrolment

Children and adults presenting to outpatient facilities at participating study sites in 8 countries across Latin America and Southeast Asia, with symptoms consistent with dengue, were screened for potential study enrolment.

Inclusion criteria

- Age ≥5 years
- Fever or history of fever for ≤72 hours
- Clinical symptoms consistent with possible dengue (suspected dengue and/or undifferentiated fever in a patient from a dengue-endemic area)
- Suitable for outpatient care (i.e. no signs of severe disease) at the time of study enrolment, at the discretion of the treating physician
- Written informed consent

Exclusion criteria

- Localising signs or symptoms suggesting an alternative diagnosis
- Unlikely to attend daily follow-up (e.g. due to travelling distance from the clinic), as judged by the treating physician

3. Important study definitions

Due to the rapidly evolving nature of clinical dengue, data collection was linked to the relevant illness day in accordance with the definitions described in Supplementary Table 1.

For Illness Day, note that although the inclusion criteria limited enrolment to within 72 hours of fever onset, in practice recruitment was aligned with the working day. Thus 87 patients who enrolled between 72-84 hours from fever onset (i.e. within 3.5 days) were considered as enrolling on illness day 3.

Supplementary Table 1: Study definitions for time-dependent events

Term	Definition
Illness day	The duration in days from the date of fever onset to the date of interest, including that day.
Study day	The duration in days from study enrolment to the current day, where the enrolment day is considered as day 1
Early phase	Period between illness days 1 and 5
Convalescent phase	Period between illness days 6 and 10
Acute illness	Period between illness days 1 and 10
Acute illness visit	Any study visit (either out-patient or in-patient) between enrolment and the day when the patient had fully recovered, up to a maximum of 6 days from study enrolment
Final acute illness visit	The last study visit during the acute illness
Follow-up visit	An out-patient study visit after recovery, between illness days 10 and 14. Very rarely, for hospitalised participants not discharged by day 14, this visit was performed by study staff in the hospital.

4. Clinical and laboratory assessments and clinical management

Following informed consent, a detailed clinical assessment was recorded in the case report form (CRF) at study enrolment and then daily until the individual had fully recovered, or for up to 6 days from enrolment. Demographic information, including age and sex (self-reported or as indicated by a parent/guardian), as well as past history, relevant family history etc. were also recorded at the enrolment visit. All patients were also asked to attend a follow-up visit between illness days 10-14, at which time a final clinical summary of the illness episode was completed. Individuals who did not attend the follow-up visit were contacted by telephone and asked to provide summary information of their progress since the final acute illness visit.

Management decisions throughout the acute illness were at the discretion of the responsible physicians, independent of study staff. Any patient requiring hospital admission continued to be followed daily by study staff up to study day 6. For the analysis, hospitalisation refers to all hospital admissions except those driven

only by social or practical reasons, such as "a sibling already in hospital with dengue", "parent concerned that the child may transmit infection to others".

A full blood count was performed at each acute illness visit, and a 3-5 ml (age-dependent) research blood sample was obtained at enrolment, the final acute illness visit, and the follow-up visit. Biochemistry profiles were performed at enrolment and intermittently during the acute illness; however, the range and frequency of tests done after study enrolment varied depending on local capacity.

All research tests and study medical consultations were paid for by the study, to ensure that participation did not result in cost implications for the patient or family. Travel costs at appropriate local rates were reimbursed for attendance at follow-up visits, but no other financial incentives were offered.

5. Dengue diagnostic testing and definitions

DENV:	Dengue virus
RT-PCR	Reverse transcription-polymerase chain reaction
NS1:	Non-structural protein 1
IgM:	Immunoglobulin M
IgG:	Immunoglobulin G
ELISA:	Enzyme linked immunosorbent assay
JEV:	Japanese encephalitis virus

Dengue diagnostic testing included DENV RT-PCR, NS1 antigen detection (Platelia NS1, Biorad) and DENV IgM and IgG Capture ELISAs (Panbio, Australia), performed in accordance with established methodology[1] and/or the manufacturers' instructions. Tests were performed in batches at a designated laboratory in each country, or at the OUCRU laboratory in HCMC if in-country diagnostics were not feasible. NS1 detection and viral RT-PCR were attempted on all enrolment plasma samples while IgG serology was performed on paired plasma samples.

Confirmed dengue

Participants were classified as having confirmed dengue if positive on either RT-PCR or NS1 testing on the enrolment sample. Given the potential for cross-reactivity with other locally circulating flaviviruses such as Zika and JEV, serological responses were not considered for the confirmed dengue group.

Other diagnostic categories

Participants who were negative on both RT-PCR and NS1 testing were classified into the following diagnostic groups based on their serological responses:

- Acute flavivirus infection: either IgM or IgG seroconversion on paired samples
- Recent flavivirus infection: IgM or IgG positive in a single sample or in paired samples
- Not dengue: both IgG and IgM serology negative on paired samples with the second sample obtained >= 6 days after illness onset and >2 days after the first sample
- Inconclusive: all other circumstances

Classification of immune status

- Probable primary infection: negative or equivocal dengue-specific IgG results on two consecutive specimens taken at least 2 days apart, with at least one specimen obtained in the convalescent phase (illness days 6-10). Of note, patients without an IgG result in the convalescent phase but with a negative or equivocal IgG after day 10 were considered to have had a negative or equivocal IgG between illness days 6-10.
- Probable secondary infection: positive dengue-specific IgG identified in the early phase (illness days 1-5) and/or convalescent phase (illness days 6-10).
- Inconclusive immune status: All other cases, due to the absence of suitable specimens at appropriate time-points.

6. Definitions for clinical outcomes

The clinical outcome of interest for this analysis was moderate/severe dengue, which we defined as development of moderate/severe plasma leakage or moderate/severe bleeding or moderate/severe neurological involvement or moderate/severe liver involvement or moderate/severe involvement of another organ. Each component is defined in Supplementary Table 2 below.

All information available in the CRF up to and including a particular day/date was used to assign the outcome for that day/date. A similar process was applied for all acute illness days for which data were available. For all moderate/severe outcomes, the first occasion when the necessary criteria for the respective outcome were fulfilled was considered as the day/date of first occurrence.

We required a minimum dataset in order to derive a meaningful clinical outcome for individual participants. In addition to enrolment data, we considered either of the following scenarios as providing adequate information:

- at least 3 acute illness visits during illness days 4-7 (critical phase for complications),

- at least 1 acute illness visit during illness days 4-7 (critical phase for complications) together with an informative final follow-up visit summary

Supplementary Table 2: Working definitions for all components of the clinical outcome

Component	Definition
**Plasma leakage**	
Severe	Clinical shock and/or respiratory distress due to plasma leakage noted in the CRF by the study doctor
Moderate	Did not fulfill criteria for severe plasma leakage AND there was evidence of haemoconcentration* ≥ 20% and/or clinical or radiological evidence of fluid accumulation
Neither moderate nor severe	Did not fulfill criteria for severe or moderate plasma leakage AND intravenous fluid was not given for any of the following reasons - rehydration, bleeding, rising haematocrit - AND patient was afebrile at the last acute illness visit
Undetermined	All other situations
**Bleeding**	
Severe	Received packed red cells and/or whole blood during the acute illness for any reason except known pre-existing anaemia (with a consistent enrolment haemoglobin value), and/or occurrence of specific rare complications such as bleeding into a critical organ (brain, spinal cord)
Moderate	Severe bleeding noted in the CRF by study doctor but no supporting evidence for the severe criteria noted above, and/or the patient received blood products such as FFP, cryoprecipitate, or platelets but NOT packed red cells or whole blood, and/or the patient required an intervention for bleeding (eg nasal packing), and/or the patient received packed red cells/whole blood but had pre-existing anaemia according to past medical history plus a consistent haemoglobin value
Neither moderate nor severe	All other situations
**Neurological involvement**	
Severe	Any neurological signs/symptoms in a patient who was admitted, or occurrence of a convulsion accompanied by any other neurological signs/symptoms even if the patient was not hospitalised
Moderate	Single convulsion without other neurological signs/symptoms or need for hospitalisation
Neither moderate nor severe	All other situations
**Liver involvement**	
Severe	Visible jaundice and/or coagulopathy and/or encephalopathy
Moderate	ALT >= 400 IU/L and/or AST >= 400 IU/L
Neither moderate nor severe	ALT and/or AST available and all values < 400 IU/L
Undetermined	No ALT or AST values available between enrolment and illness day 10
**Other major organ failure**	
Severe	CK abnormalities (or other enzymes if available [eg troponin]) AND functional abnormalities (reduced EF <50% or new ECG abnormalities) and/or specific intervention needed (eg inotropic support)
Moderate	Troponin and/or CK abnormalities AND a specific diagnostic intervention (eg cardiac ultrasound, cardiac MRI) but without EF <50%
Neither moderate nor severe	All other situations

Legend for Supplementary Table 2:

*Haemoconcentration = (peak haematocrit – baseline haematocrit)/baseline haematocrit, where:
- peak haematocrit is defined as the maximum documented haematocrit between illness day 4-7
- baseline haematocrit is defined as the minimum documented haematocrit between illness days 1-3

ALT: Alanine aminotransferase. AST: Aspartate aminotransferase. CK: Creatine Kinase

EF: Ejection fraction. ECG: Electrocardiogram. MRI: Magentic resonance imaging.

To derive and verify individual clinical outcomes, we developed a computer algorithm based on these criteria and definitions and used it to automatically classify the clinical outcome for each patient. For all those who met the criteria for moderate/severe dengue, an endpoint review committee (BW, TJ, KR, LP) carefully checked the information in the CRF and, where necessary, verified the final category using additional information from hospital records and/or personal communication with local researchers.

Patients for whom the minimum dataset was available for all the components of the clinical outcome, but who did not meet the criteria for either moderate or severe dengue, were classified as uncomplicated.

By contrast, patients for whom the clinical outcome could not be defined due to missing information were excluded from the analyses.

7. Candidate predictors

Selection of candidate predictors

From a total of 65 variables available in the database, 29 were considered as candidate predictors. To be selected, these variables must:

- Not be included in any of the outcome definitions
- Have less than 10% missing data at baseline (see Supplementary Table 6)
- For clinical signs and symptoms, occur in 5-95% of participants
- For laboratory tests, be readily available in clinical practice in participating centres

Additionally, since we wished to develop models that use baseline information (Baseline Model) and models that use daily information on signs/symptoms and laboratory tests (Dynamic Models), such variables were only considered as candidate predictors if they featured among the assessments due to be recorded at each acute illness visit according to the study protocol.

As hemoconcentration was included in the definition of plasma leakage, haematocrit was not considered as candidate predictor.

Definitions for the selected candidate predictors are given in Supplementary Table 3.

Combination and transformation of candidate predictors

Signs and symptoms that were strongly correlated (overall agreement index > 0.7) were combined into composite variables: “Abdominal pain” and “abdominal tenderness” become “abdominal pain/tenderness”, “Joint pain” and “muscle pain” become “joint/muscle pain”, “Lethargy” and “restlessness” become “lethargy/restlessness”

Total white blood cell count (WBC) was log-transformed before the analysis due to its skewed distribution.

Candidate predictors used in the dynamic models

To limit the complexity of the final dynamic models, only well-recognised warning signs (persistent vomiting, lethargy/restlessness, significant abdominal pain/tenderness) and continuous variables (body temperature, platelet count, total WBC, lymphocyte percentage) were considered for inclusion in these models.

For the Dynamic-2 Model continuous variables, the change variable was the absolute difference between the current value and the previous value.

For the Dynamic-2 Model binary variables, the change variable had 3 possible values, defined by data on the previous day and the current day, as summarised below:

**Previous value**	**Current value**	**Value of the change variable**
- No	- No	- Absent
- Yes	- Yes/No	- Already occurred
- No	- Yes	- New occurrence

Supplementary Table 3: Definitions for candidate predictors

Candidate predictor	Definition	Type	Value
Age	Age	Continuous	Age in years
Sex	Sex (self-reported)	Binary	Female or Male
Continent	Study continent	Binary	Asia (Vietnam, Cambodia, Bangladesh, Indonesia, Malaysia),<br>Latin America (Brazil, El Salvador, Venezuela)
Past medical history	Any past medical history	Binary	Yes if at least one entry in the past medical history form is "Yes"<br>No if all entries in the past medical history form are "No"
Illness day at enrolment	Time since fever onset at enrolment	Categorical	Day 1, 2, 3
Chills*	Chills	Binary	Yes, No
Vomiting*	Persistent vomiting	Binary	Yes if >5 vomits in last 24 hours, No if no vomiting OR ≤5 vomits in last 24 hours
Cough*	Cough	Binary	Yes, No
Diarrhoea*	Passage of 3 or more liquid stools in a 24 hour period	Binary	Yes, No
Abdominal pain or tenderness*	Significant abdominal pain - interferes with or significantly impacts on activities of daily living but is relieved or improved with simple analgesia (++) or strong analgesia e.g. opiate-based medication (+++)) or if abdominal tenderness is elicited	Binary	Yes if significant abdominal pain (severity of ++/+++) or abdominal tenderness are present<br>No if both are absent
Headache*	Headache	Binary	Yes, No
Joint or muscle pain*	Joint pain or muscle pain	Binary	Yes if joint pain or muscle pain are present, No if both joint pain and muscle pain are absent
Retro-orbital pain*	Retro-orbital pain	Binary	Yes, No
Dizzy*	Dizziness	Binary	Yes, No
Temperature*	Axillary temperature. A single value recorded at the current outpatient visit, OR the maximum value recorded in the previous 24 hours for in-patients	Continuous	Body temperature measured in degrees Celsius
Respiratory rate*	Respiratory rate. A single value recorded at the current outpatient visit, OR the maximum value recorded in the previous 24 hours for inpatients	Numeric	Respiratory rate per minute
Pulse*	Pulse rate. A single value recorded at the current outpatient visit, OR the maximum value recorded in the previous 24 hours for inpatients	Numeric	Pulse rate per minute
Systolic blood pressure*	Systolic blood pressure. A single value recorded at the current outpatient visit, OR the minimum value recorded in the previous 24 hours for inpatients	Continuous	Systolic blood pressure measured in mmHg
Pulse pressure*	Pulse pressure	Continuous	Difference in mmHg between systolic BP and diastolic BP
Skin rash*	Skin rash (inflammatory rashes only - macular, maculopapular, vesicular or recovery rash)	Binary	Yes, No
Skin flush*	Skin flush (a red appearance to the skin that blanches with pressure)	Binary	Yes, No
Lymphadenopathy*	Swelling or enlargement of the lymph nodes for any reason	Binary	Yes, No
Conjunctival injection*	Presence of abnormal redness on the sclera of the eye due to hyperemia of the local blood vessels. In patients with dengue it is most often seen inside the eyelid, rather than on the white sclera. It should be distinguished from redness due to local bleeding – i.e. subconjunctival haemorrhage.	Binary	Yes, No
Rhinitis*	Presence of nasal discharge due to irritation or inflammation of the nasal mucosa	Binary	Yes, No
Lethargy or restlessness*	Lethargy (feeling so tired it is difficult to get out of bed to go to the toilet) or restlessness (increased psychomotor activity, an observed clinical sign)	Binary	Yes if lethargy or restlessness are present, No if both lethargy or restlessness are absent
Bleeding*	Bleeding	Categorical	Mucosal bleeding – observed or reported bleeding from the nose, oral cavity, the gastrointestinal tract (including vomiting of blood or the passage of melaena), or haematuria. In female postpubertal patients unusual vaginal bleeding is also included – i.e. unusually heavy menstrual bleeding for that individual or unexpected inter-menstrual bleeding. (With or without skin bleeding.)<br>Skin only bleeding – the presence of petechiae or spontaneous bruising without evidence of mucosal bleeding<br>None if no bleeding
Platelet count*	Lowest platelet count	Numeric	Platelet count in 1000 per mm3
Total WBC count*	Total WBC count	Numeric	Total WBC count in 1000 per mm3
% lymphocytes*	Percentage of lymphocytes	Numeric	Percentage of lymphocytes in total WBC count

Legend for Supplementary Table 3:

* Variables recorded for a given study day include all events in the preceding 24 hours since the last assessment

WBC: white blood cell count

SUPPLEMENTARY NOTES

1. Missing data

There were 464/2694 patients (17%) for whom the status of moderate/severe dengue could not be defined and these individuals were excluded from the analysis.

For predictor variables, the percentage of missing data was generally low (Supplementary Table 9). At enrolment, there was less than 1% missing data per variable except for laboratory tests (creatinine, creatinine kinase, immune status, serotype and viremia), and only 100/2230 (4%) of patients had one or more missing candidate predictors at this time. On subsequent days the percentage of missing data was generally less than 5%, but increased up to a maximum of 16% towards the end of the observation period; however, data on biochemical tests are not included in this summary since these tests were not scheduled to be performed every day at all sites. A total of 61/2230 (3%) of patients had information on one or more candidate predictors missing on each observation day between illness day 1 to 8.

We performed sensitivity analyses to explore the impact of missing data on the model assumptions (Supplementary Tables 10-11), coefficients (Supplementary Table 12), and model performance (Supplementary Fig. 1). Missing data at enrolment and during follow-up were imputed using the following procedure:

- Missing data at enrolment: we used the most common category (for categorical variables) or the median value (for continuous variables) from patients with complete data to impute for missing values in the corresponding variable.
- Missing data during follow-up: missing data on any specific day for a particular patient were imputed using the last non-missing data value from the preceding days for that patient.

Results from the assumption assessments are in line with results using complete case data (Supplementary Tables 10-11). There are no major differences in the variables selected in the final models or the magnitude of their coefficients, between the analyses (Supplementary Table 12). The apparent performance of the final models is consistent between the two analyses, when sample sizes are sufficient (i.e. in all patients and in the Asian populations) (Supplementary Fig. 1). This suggests that there is little impact of the missing data on the overall results.

2. Heterogeneity assessment

We explored heterogeneities in enrolment patient characteristics and outcomes between study sites (Fig. 1 and Supplementary Table 13), as well as their potential impact on model performance (Supplementary Table 14), following the principles outlined by Steyerberg EW et al[2]. We explored differences in patient characteristics by using membership c-statistics, which reflect the discriminative ability to separate a specific study site from all other sites combined, based on patient characteristics. We explored differences in predictions between study sites by assessing the distribution of the linear predictors obtained from fitting the full baseline prediction model on all participants.

Supplementary Table 13 shows that there were notable differences in enrolment numbers, prevalence of the moderate/severe outcome, and age distribution between study sites. Baseline profiles were also different across the sites; using this information, especially age, it was possible to differentiate patients enrolled at a certain site from patients enrolled at the other sites (reflected in the membership c-statistics). However, these heterogeneities in patient characteristics are not likely to have much impact on outcome prediction as the distribution of the fitted linear predictors is fairly similar over the sites (mean ranges from -3.03 to -1.22 for all sites, and from -2.88 to -1.82 for sites with at least 10 patients). These results suggest considerable differences in patient characteristics at enrolment and in clinical outcome profiles between study sites, which can be attributed largely to a difference in age distribution. However, the effect of this heterogeneity on prediction was not strong, as the distribution of the linear predictor when applying the full baseline prediction model at each site separately does not indicate notable differences.

Supplementary Table 14 shows that the AUCs by site range from 0.59 to 0.79 but the 95% confidence intervals largely overlap. Regarding calibration, the models applied to sites 2 and 81 (sites with low prevalence of moderate/severe dengue) overestimate the prevalence of the outcome, while the models applied to site 3 (a site with high prevalence of moderate/severe dengue) underestimate the prevalence of the outcome. By continent, the model developed in Latin America and applied to Asia is overfitted, while the confidence intervals of the AUCs for the two continents overlap. In addition, the model applied to site 31 is overfitted with a calibration slope significantly less than 1.

With respect to prediction performance there is potential for overfitting and also miscalibration-in-the-large for sites with outcome prevalences at the extremes. However, discriminative ability is unlikely to be affected.

Supplementary Table 4: Summary of the different analysis populations.

Analysis population	Description	Analysis	Sample size				
			**Total**	**Non-moderate/severe**	**Moderate/severe dengue**	**Severe dengue ***	**Severe dengue only ****
Descriptive analysis population	Patients with:<br>- lab-confirmed dengue<br>- sufficient data to define moderate/severe dengue	Descriptive analysis	2230	1926	304	38	25
Univariable analysis population	Patients with:<br>- lab-confirmed dengue<br>- sufficient data to define moderate/severe dengue<br>- outcome occurred after enrolment	Univariable analysis	2206	1926	280	37	24
Model development population	Patients with:<br>- lab-confirmed dengue<br>- sufficient data to define moderate/severe dengue<br>- outcome occurred after enrolment<br>- complete predictors data at enrolment	Model development<br><br>Exploratory analysis<br><br>Utility analysis	2130	1856	274	37	24
Imputed dataset	Dataset of the univariable analysis population but using simple imputation method to impute missing data	Sensitivity analysis	2206	1926	280	37	24

Legend for Supplementary Table 4:

* includes both patients in whom severe dengue developed without an intermediate moderate phase, and those in whom moderate dengue occurred before severe dengue

** excludes patients in whom moderate dengue occurred before severe dengue

Supplementary Table 5: Summary of linearity assessment (n = 2130).

Covariate	Illness Day 1 (n = 342)		Illness Day 2 (n = 1177)		Illness Day 3 (n = 2034)		All Baseline Data (n = 2130)	
	**Deviance**	**p-value**	**Deviance**	**p-value**	**Deviance**	**p-value**	**Deviance**	**p-value**
Age	3.58	0.311	1.99	0.575	3.70	0.295	2.50	0.475
Temperature	3.52	0.318	5.21	0.157	1.58	0.664	10.36	0.016
Respiratory rate	0.73	0.866	0.54	0.910	0.34	0.952	3.58	0.311
Pulse rate	0.69	0.875	2.92	0.403	5.02	0.171	3.28	0.350
Systolic blood pressure	1.27	0.732	1.53	0.675	6.91	0.075	1.87	0.599
Pulse pressure	3.29	0.348	1.56	0.668	2.62	0.453	1.98	0.577
Platelet count	1.32	0.724	6.14	0.105	8.79	0.032	12.86	0.005
Total while blood cell count	2.08	0.557	15.32	0.002	6.90	0.075	13.95	0.003
% Lymphocyte	1.82	0.610	0.28	0.964	3.41	0.333	6.76	0.080

Legend for Supplementary Table 5:

Deviances and P-values were based on two-sided likelihood ratio tests comparing the model that included all continuous variables as restricted cubic spline with 4 degrees of freedom with one that modelled the continuous variable of interest as a linear term.

Illness day 1, Illness day 2, Illness day 3, refer to complete case datasets with information for illness day 1, illness day 2, illness day 3, respectively. In the last column all the baseline data is collated into one combined dataset.

Supplementary Table 6. Summary of interaction assessment (n = 2130).

Interaction test	Illness Day 1 (n = 342)	Illness Day 2 (n = 1177)	Illness Day 3 (n = 2034)	All Baseline Data (n = 2130)
Overall interaction test	0.006	0.017	0.129	0.013
Continent * Age	0.001	0.002	0.084	0.002
Continent * Chills	0.012	0.532	0.737	0.282
Continent * Lethargy/Restlessness	-	0.884	-	0.734
Continent * Dizzyness	0.001	0.227	0.110	0.025
Continent * Rash	0.010	0.239	0.726	0.086
Continent * Flush	0.999	0.917	0.539	0.391
Age * Lethargy/Restlessness	0.224	0.009	0.379	0.029
Age * Persistent vomiting	0.749	0.569	0.045	0.848
Age * Significant abdominal pain/tenderness	0.546	0.525	0.925	0.410
Age * Platelet count	0.356	0.903	0.141	0.869
Age * Total white blood cell count	0.873	0.837	0.639	0.584
Age * % Lymphocyte	0.158	0.508	0.984	0.221

Legend for Supplementary Table 6:

Values are p-values derived from two-sided likelihood ratio tests comparing the model that included all pre-defined interactions between continent and age with the selected covariates and the model that excluded the interaction of interest. Each individual interaction was only considered if the overall interaction test for that model (simultaneous test of all interactions) had a p-value < 0.05.

Illness day 1, Illness day 2, Illness day 3, refer to complete case datasets with baseline information for illness day 1, illness day 2, illness day 3, respectively. In the last column all the baseline data is collated into one combined dataset

Supplementary Table 7: Optimism-corrected AUC of models with and without interaction assessment in the development procedure (n = 2130).

Evaluation	With all interactions	Without interactions
Baseline model	0.682	0.674
Dynamic-1 model		
Study day 1	0.684	0.673
Study day 2	0.735	0.731
Study day 3	0.799	0.797
Study day 4	0.851	0.849
Dynamic-2 model		
Study day 1	-	-
Study day 2	0.744	0.740
Study day 3	0.799	0.794
Study day 4	0.853	0.849

Legend for Supplementary Table 7:

Values are optimism-corrected Area Under the ROC Curves (AUCs) of three prediction models (Baseline, Dynamic-1, Dynamic-2) included and excluded all interactions.

Supplementary Table 8: Enrolment characteristics of participants with confirmed dengue who were excluded from or included in the analysis population (n = 2694).

Characteristic	Excluded (N=464)		Included (N=2230)	
	n	Summary	n	Summary
Continent	464		2230	
Asia		388 (84)		1850 (83)
Latin America		76 (16)		380 (17)
Country	464		2230	
Cambodia		79 (17)		223 (10)
Indonesia		53 (11)		39 (2)
Malaysia		54 (12)		205 (9)
Vietnam		179 (38)		1326 (59)
Bangladesh		23 (5)		57 (3)
Brazil		57 (12)		54 (2)
El Salvador		10 (2)		296 (13)
Venezuela		9 (2)		30 (1)
Year of enrollment	464		2230	
2011		14 (3)		108 (5)
2012		77 (17)		458 (21)
2013		107 (23)		520 (23)
2014		115 (25)		529 (24)
2015		150 (32)		615 (28)
2016		1 (<1)		0 (0)
Age [years]	464	17 (10, 24)	2230	14 (9, 24)
Child (<15 years old)	464	202 (44)	2230	1151 (52)
Sex: Female	464	194 (42)	2230	934 (42)
Illness day at enrollment	464		2230	
1		148 (32)		360 (16)
2		189 (41)		906 (41)
3		127 (27)		964 (43)
Viremia at enrolment [log-10 copies/mL]	338	7.3 (6.0, 8.5)	1951	7.3 (6.2, 8.2)
Viral serotype	346		1982	
DENV-1		199 (57)		882 (45)
DENV-2		58 (17)		302 (15)
DENV-3		35 (10)		311 (16)
DENV-4		54 (16)		487 (25)
Immune status	464		2230	
Probable primary		73 (16)		501 (22)
Probable secondary		121 (26)		1341 (60)
Inconclusive serostatus		270 (58)		388 (17)

Legend for Supplementary Table 8:

N is the number of all patients; n is the number of patients with non-missing values.

Summary is the absolute count (%) for categorical variable(s) and median (1st and 3rd quartiles) for numeric variable(s).

Supplementary Table 9: Description of missing data for the analysis population (n = 2230).

Variable	Baseline	Illness day							
		1	2	3	4	5	6	7	8
N	2230	360	1264	2222	2221	2031	1536	776	203
**Age ***	0 (<1)	-	-	-	-	-	-	-	-
**Sex ***	0 (<1)	-	-	-	-	-	-	-	-
**Past medical history ***	15 (<1)	-	-	-	-	-	-	-	-
**Chills**	3 (<1)	0 (<1)	5 (<1)	1 (<1)	3 (<1)	1 (<1)	4 (<1)	9 (1)	14 (7)
Anorexia	0 (<1)	0 (<1)	1 (<1)	1 (<1)	3 (<1)	0 (<1)	4 (<1)	9 (1)	13 (6)
Nausea	3 (<1)	2 (<1)	2 (<1)	2 (<1)	3 (<1)	0 (<1)	4 (<1)	9 (1)	13 (6)
**Persistent vomiting**	1 (<1)	0 (<1)	3 (<1)	2 (<1)	2 (<1)	1 (<1)	4 (<1)	9 (1)	13 (6)
**Cough**	0 (<1)	0 (<1)	21 (2)	73 (3)	114 (5)	117 (6)	107 (7)	76 (10)	30 (15)
**Diarrhoea**	0 (<1)	0 (<1)	2 (<1)	2 (<1)	2 (<1)	2 (<1)	4 (<1)	9 (1)	13 (6)
Significant abdominal pain	1 (<1)	0 (<1)	3 (<1)	2 (<1)	5 (<1)	4 (<1)	5 (<1)	9 (1)	13 (6)
Significant abdominal tenderness	0 (<1)	0 (<1)	6 (<1)	19 (<1)	45 (2)	45 (2)	38 (2)	31 (4)	19 (9)
**Significant abdominal pain/tenderness**	7 (<1)	0 (<1)	12 (<1)	27 (1)	51 (2)	51 (3)	40 (3)	31 (4)	19 (9)
**Headache**	0 (<1)	0 (<1)	2 (<1)	2 (<1)	2 (<1)	1 (<1)	5 (<1)	9 (1)	13 (6)
Joint pain	0 (<1)	0 (<1)	2 (<1)	3 (<1)	2 (<1)	1 (<1)	5 (<1)	10 (1)	13 (6)
Muscle pain	1 (<1)	0 (<1)	3 (<1)	2 (<1)	2 (<1)	1 (<1)	5 (<1)	10 (1)	13 (6)
**Joint or muscle pain**	1 (<1)	0 (<1)	3 (<1)	3 (<1)	2 (<1)	1 (<1)	5 (<1)	10 (1)	13 (6)
**Retro-orbital pain**	3 (<1)	1 (<1)	3 (<1)	3 (<1)	2 (<1)	1 (<1)	5 (<1)	10 (1)	13 (6)
**Dizzyness**	4 (<1)	0 (<1)	4 (<1)	4 (<1)	2 (<1)	1 (<1)	4 (<1)	10 (1)	13 (6)
Sore throat *	3 (<1)	-	-	-	-	-	-	-	-
**Temperature**	1 (<1)	1 (<1)	8 (<1)	25 (1)	54 (2)	61 (3)	50 (3)	33 (4)	20 (10)
**Respiratory rate**	4 (<1)	1 (<1)	9 (<1)	29 (1)	53 (2)	65 (3)	50 (3)	34 (4)	20 (10)
**Pulse rate**	2 (<1)	0 (<1)	9 (<1)	27 (1)	53 (2)	62 (3)	49 (3)	33 (4)	20 (10)
**Systolic blood pressure**	6 (<1)	1 (<1)	14 (1)	33 (1)	57 (3)	62 (3)	51 (3)	35 (5)	20 (10)
Diastolic blood pressure	6 (<1)	1 (<1)	14 (1)	33 (1)	57 (3)	62 (3)	51 (3)	35 (5)	20 (10)
**Pulse pressure**	6 (<1)	1 (<1)	14 (1)	33 (1)	57 (3)	62 (3)	51 (3)	35 (5)	20 (10)
**Skin rash**	11 (<1)	2 (<1)	12 (<1)	22 (<1)	40 (2)	40 (2)	37 (2)	31 (4)	19 (9)
**Skin flush**	1 (<1)	1 (<1)	6 (<1)	18 (<1)	41 (2)	43 (2)	38 (2)	31 (4)	19 (9)
**Lymphadenopathy**	0 (<1)	0 (<1)	6 (<1)	19 (<1)	41 (2)	43 (2)	39 (3)	33 (4)	19 (9)
**Conjunctival injection**	0 (<1)	0 (<1)	6 (<1)	18 (<1)	40 (2)	44 (2)	37 (2)	30 (4)	19 (9)
Pharyngeal injection	2 (<1)	1 (<1)	7 (<1)	20 (<1)	40 (2)	43 (2)	37 (2)	30 (4)	19 (9)
**Rhinitis**	1 (<1)	0 (<1)	6 (<1)	19 (<1)	39 (2)	43 (2)	36 (2)	30 (4)	19 (9)
Oedema	0 (<1)	0 (<1)	6 (<1)	19 (<1)	40 (2)	44 (2)	37 (2)	31 (4)	19 (9)
Clinical pleural effusion	0 (<1)	0 (<1)	6 (<1)	19 (<1)	41 (2)	44 (2)	37 (2)	31 (4)	19 (9)
Rales	0 (<1)	0 (<1)	6 (<1)	19 (<1)	42 (2)	45 (2)	37 (2)	31 (4)	19 (9)
Jaundice	2 (<1)	1 (<1)	7 (<1)	19 (<1)	42 (2)	44 (2)	38 (2)	31 (4)	19 (9)
Ascites	0 (<1)	0 (<1)	6 (<1)	19 (<1)	46 (2)	46 (2)	39 (3)	32 (4)	19 (9)
Liver palpable	0 (<1)	0 (<1)	6 (<1)	19 (<1)	45 (2)	47 (2)	38 (2)	31 (4)	19 (9)
Liver enlargement[a]	0 (<1)	0 (<1)	6 (<1)	19 (<1)	45 (2)	47 (2)	38 (2)	31 (4)	19 (9)
Spleen palpable	0 (<1)	0 (<1)	6 (<1)	19 (<1)	45 (2)	47 (2)	39 (3)	31 (4)	19 (9)
Lethargy	0 (<1)	0 (<1)	6 (<1)	17 (<1)	35 (2)	38 (2)	37 (2)	31 (4)	19 (9)
Restlessness	4 (<1)	2 (<1)	8 (<1)	17 (<1)	37 (2)	41 (2)	37 (2)	31 (4)	19 (9)
**Lethargy or restlessness**	3 (<1)	1 (<1)	8 (<1)	17 (<1)	36 (2)	41 (2)	38 (2)	31 (4)	19 (9)
Convulsions **	2230 (100)	360 (100)	912 (72)	981 (44)	37 (2)	41 (2)	37 (2)	31 (4)	19 (9)
Bruising	0 (<1)	0 (<1)	6 (<1)	15 (<1)	35 (2)	36 (2)	36 (2)	30 (4)	19 (9)
Petechiae	1 (<1)	0 (<1)	7 (<1)	15 (<1)	35 (2)	36 (2)	36 (2)	30 (4)	19 (9)
Gum bleeding	0 (<1)	0 (<1)	6 (<1)	15 (<1)	35 (2)	36 (2)	36 (2)	30 (4)	19 (9)
Nose bleeding	0 (<1)	0 (<1)	6 (<1)	15 (<1)	35 (2)	36 (2)	36 (2)	30 (4)	19 (9)
Hematemesis	0 (<1)	0 (<1)	6 (<1)	15 (<1)	35 (2)	36 (2)	36 (2)	30 (4)	19 (9)
Melaena	1 (<1)	1 (<1)	6 (<1)	15 (<1)	35 (2)	36 (2)	36 (2)	30 (4)	19 (9)
Hematuria	0 (<1)	0 (<1)	6 (<1)	15 (<1)	35 (2)	36 (2)	36 (2)	30 (4)	19 (9)
Unusual vaginal bleeding***	2 (<1)	0 (<1)	3 (<1)	7 (<1)	15 (2)	12 (1)	12 (2)	10 (3)	5 (5)
**Bleeding[b]**	0 (<1)	0 (<1)	7 (<1)	16 (<1)	41 (2)	41 (2)	38 (2)	31 (4)	19 (9)
**Platelet count**	10 (<1)	3 (<1)	14 (1)	33 (1)	64 (3)	94 (5)	84 (5)	57 (7)	24 (12)
**Total white blood cell count**	11 (<1)	4 (1)	14 (1)	33 (1)	64 (3)	94 (5)	83 (5)	58 (7)	24 (12)
% neutrophils	22 (<1)	4 (1)	20 (2)	46 (2)	83 (4)	110 (5)	103 (7)	74 (10)	33 (16)
**% lymphocytes**	20 (<1)	4 (1)	18 (1)	46 (2)	81 (4)	110 (5)	102 (7)	74 (10)	33 (16)
% monocytes	21 (<1)	4 (1)	19 (2)	46 (2)	84 (4)	111 (5)	104 (7)	75 (10)	34 (17)
Haemoglobin	11 (<1)	3 (<1)	14 (1)	36 (2)	66 (3)	94 (5)	84 (5)	57 (7)	24 (12)
Haematocrit	11 (<1)	3 (<1)	14 (1)	35 (2)	67 (3)	96 (5)	87 (6)	57 (7)	24 (12)
Albumin ****	45 (2)	9 (3)	359 (28)	1229 (55)	1907 (86)	1388 (68)	750 (49)	316 (41)	78 (38)
Aspartate aminotransferase ****	35 (2)	4 (1)	360 (28)	1217 (55)	1885 (85)	1358 (67)	724 (47)	298 (38)	67 (33)
Alanine aminotransferase ****	29 (1)	4 (1)	356 (28)	1212 (55)	1879 (85)	1347 (66)	720 (47)	296 (38)	67 (33)
Creatinine ****	90 (4)	13 (4)	390 (31)	1236 (56)	1903 (86)	1376 (68)	746 (49)	319 (41)	72 (35)
Creatine kinase ****	323 (14)	72 (20)	502 (40)	1309 (59)	1960 (88)	1452 (71)	823 (54)	369 (48)	89 (44)
Non-structural protein 1 ****	17 (<1)	1 (<1)	9 (<1)	17 (<1)	17 (<1)	13 (<1)	8 (<1)	2 (<1)	1 (<1)
Immune status ****	388 (17)	90 (25)	247 (20)	383 (17)	383 (17)	311 (15)	171 (11)	72 (9)	15 (7)
Serotype ****	248 (11)	35 (10)	130 (10)	245 (11)	246 (11)	206 (10)	127 (8)	57 (7)	17 (8)
Viremia ****	279 (13)	43 (12)	147 (12)	275 (12)	277 (12)	228 (11)	146 (10)	66 (9)	18 (9)

Legend for Supplementary Table 9:

N: total participants, -: not assessed

*: only assessed at enrollment (baseline)

**: not assessed at enrollment (baseline)

***: only assessed in females

****: not assessed daily

[a]: liver ≥2 cm below costal margin in the mid-clavicular line

[b]: whether patient had mucosal bleeding, skin only bleeding or no bleeding

Variables in bold are those included as candidate predictors in the analyses. This table includes 27//29 candidate predictors, as continent and illness day at enrolment are available by design for all patients.

Supplementary Table 10: Summary of linearity assessment (n = 2206, imputed data).

Covariate	Illness Day 1 (n = 357)		Illness Day 2 (n = 1252)		Illness Day 3 (n = 2198)		All Baseline Data (n = 2206)	
	Deviance	p-value	Deviance	p-value	Deviance	p-value	Deviance	p-value
Age	3.34	0.342	2.09	0.554	3.46	0.327	2.50	0.475
Temperature	3.79	0.285	7.01	0.072	2.13	0.546	9.81	0.020
Respiratory rate	1.10	0.776	0.51	0.917	0.23	0.973	3.56	0.313
Pulse rate	0.43	0.934	3.90	0.273	4.57	0.206	3.59	0.309
Systolic blood pressure	1.34	0.719	1.06	0.787	6.00	0.112	1.06	0.786
Pulse pressure	3.31	0.347	0.62	0.891	2.49	0.478	3.14	0.371
Platelet count	1.56	0.668	6.08	0.108	10.69	0.014	12.21	0.007
Total white blood cell count	1.61	0.657	13.63	0.003	5.55	0.136	13.25	0.004
% Lymphocyte	2.15	0.541	0.85	0.838	3.07	0.381	5.36	0.147

Legend for Supplementary Table 10:

Deviances and P-values were based on two-sided likelihood ratio tests comparing the model that included all continuous variables as restricted cubic spline with 4 degrees of freedom with one that modelled the continuous variable of interest as a linear term.

Illness day 1, Illness day 2, Illness day 3, refer to imputed datasets with information for illness day 1, illness day 2, illness day 3, respectively. In the last column all the baseline data is collated into one combined dataset.

Supplementary Table 11: Summary of interaction assessment (n = 2206, imputed data).

Interaction test	Illness Day 1 (n = 357)	Illness Day 2 (n = 1252)	Illness Day 3 (n = 2198)	All Baseline Data (n = 2206)
Overall interaction test	0.035	0.015	0.049	0.013
Continent * Age	0.046	0.005	0.017	0.003
Continent * Chills	0.566	0.996	0.525	0.356
Continent * Lethargy/Restlessness	-	0.813	0.352	0.310
Continent * Dizzyness	0.015	0.165	0.059	0.012
Continent * Rash	0.303	0.295	0.760	0.090
Continent * Flush	0.166	0.796	0.562	0.269
Age * Lethargy/Restlessness	0.246	0.007	0.179	0.040
Age * Persistent vomiting	0.843	0.552	0.112	0.863
Age * Significant abdominal pain/tenderness	0.662	0.413	0.792	0.415
Age * Platelet count	0.091	0.557	0.057	0.941
Age * Total white blood cell count	0.886	0.482	0.528	0.505
Age * % Lymphocyte	0.433	0.465	0.842	0.275

Legend for Supplementary Table 11:

Values are p-values derived from two-sided likelihood ratio tests comparing the model that included all pre-defined interactions between continent and age with the selected covariates and the model that excluded the interaction of interest. Each individual interaction was only considered if the overall interaction test for that model (simultaneous test of all interactions) had a p-value < 0.05.

Illness day 1, Illness day 2, Illness day 3, refer to imputed datasets with baseline information for illness day 1, illness day 2, illness day 3, respectively. In the last column all the baseline data is collated into one combined dataset

Supplementary Table 12: Regression coefficients of models using complete case & imputed data.

Intercept and Predictors	Logistic regression coefficients (95% confidence interval)		
	Baseline Model	Dynamic-1 Model	Dynamic-2 Model
Intercept	-5.46 (-5.8)	-6.33 (-5.8)	-11.34 (-6.0)
Continent (Latin America)	0.05 (-0.46)	-0.16 (-0.42)	-0.36 (-0.46)
Age	-0.14 (-0.12)	-0.15 (-0.15)	-0.15 (-0.16)
Chills	-0.36 (-0.27)	-0.61 (-0.52)	-
Lethargy/restlessness	-0.10 (0.00)	-0.36 (-0.47)	0.47 (-1.3)
Cough	-	-0.52 (-0.43)	-1.25 (-1.1)
Rhinitis	-	0.63 (-)	-
Conjunctival injection	0.26 (0.24)	0.21 (0.24)	0.32 (0.32)
Dizzyness	0.25 (-)	-0.03 (-0.03)	-0.07 (-0.05)
Headache	-	-0.16 (-0.40)	-0.2 (-0.59)
Retro-orbital pain	-	- (-0.15)	-
Joint/muscle pain	0.40 (0.37)	0.17 (0.22)	-
Persistent vomiting	0.38 (0.40)	0.08 (0.19)	-0.03 (-0.34)
Diarrhoea	-	0.19 (0.23)	0.21 (0.3)
Significant abdominal pain/tenderness	0.48 (0.51)	0.26 (0.20)	-0.24 (-0.25)
Bleeding (Skin only)	-0.61 (-0.60)	-1.1 (-1.0)	-1.17 (-1.1)
Bleeding (Mucosal bleeding)	-0.16 (-0.16)	0.11 (0.01)	0.09 (-0.05)
Temperature	-	0.01 (-0.02)	0.3 (0.3)
Respiratory rate	-	- (-0.23)	- (-0.4)
Pulse	-	-0.15 (-0.05)	-0.33 (-0.1)
Pulse pressure	0.05 (-)	0.09 (0.06)	0.04 (0.01)
% Lymphocyte	-0.22 (-0.23)	-0.29 (-0.28)	-0.43 (-0.25)
Illness day at enrolment (day 2)	0.03 (0.05)	-0.12 (-0.11)	-0.02 (-0.09)
Illness day at enrolment (day 3)	-0.36 (-0.39)	-0.66 (-0.66)	-0.59 (-0.63)
Platelet count (when ≤ 150,000)	-0.15 (-0.14)	-0.13 (-0.12)	-0.1 (-0.11)
Platelet count (when > 150,000)	-0.02 (-0.03)	-0.07 (-0.08)	-0.02 (-0.02)
Total white blood cell count (when ≤ 2,000)	-2.35 (-2.5)	-4.46 (-4.1)	-8.76 (-4.2)
Total white blood cell count (when > 2,000)	-0.01 (-0.01)	-0.23 (-0.12)	-0.6 (-0.44)
Continent (Latin America) * Age	0.34 (0.28)	0.33 (0.27)	0.35 (0.29)
Continent (Latin America) * Dizzyness	-1.16 (-)	-0.66 (-)	-
Age * Lethargy/restlessness	0.31 (0.25)	0.46 (0.45)	- (0.57)
Day of illness	-	0.5 (0.38)	1.23 (0.24)
Day of illness * Chills	-	0.16 (0.15)	-
Day of illness * Lethargy/restlessness	-	0.18 (0.22)	- (0.41)
Day of illness * Cough	-	0.18 (0.16)	0.34 (0.29)
Day of illness * Rhinitis	-	-0.21 (-)	-
Day of illness * Dizzyness	-	0.09 (0.08)	0.09 (0.09)
Day of illness * Headache	-	0.08 (0.15)	0.09 (0.2)
Day of illness * Persistent vomiting	-	0.11 (0.07)	0.09 (0.18)
Day of illness * Bleeding (Skin only)	-	0.22 (0.2)	0.25 (0.23)
Day of illness * Bleeding (Mucosal bleeding)	-	-0.14 (-0.12)	-0.12 (-0.10)
Day of illness * Temperature	-	0.06 (0.07)	-
Day of illness * Respiratory rate	-	- (0.06)	- (0.09)
Day of illness * Pulse	-	0.04 (-)	0.07 (-)
Day of illness * Pulse pressure	-	-0.03 (-0.03)	-0.02 (-0.02)
Day of illness * % Lymphocyte	-	0.03 (0.02)	0.04 (-)
Day of illness * Platelet count (when > 150,000)	-	0.02 (0.02)	-
Day of illness * Platelet count (when ≤ 150,000)	-	0 (0)	-
Day of illness * Total white blood cell count (when ≤ 2,000)	-	0.91 (0.85)	1.55 (0.71)
Day of illness * Total white blood cell count (if > 2,000)	-	-0.03 (-0.05)	0.07 (0.03)
Age * Day of illness * Lethargy/restlessness	-	-0.08 (-0.09)	- (-0.11)
Change in persistent vomiting (already occurred)	-	-	0.34 (0.63)
Change in persistent vomiting (new occur)	-	-	0.12 (1.4)
Change in significant abdominal pain/tenderness (already occurred)	-	-	0.37 (0.22)
Change in significant abdominal pain/tenderness (new occur)	-	-	0.53 (0.48)
Change in % Lymphocyte	-	-	0.1 (-0.03)
Change in Platelet count (when ≤ 150,000)	-	-	0.09 (0.06)
Change in Platelet count (when > 150,000)	-	-	0.01 (0.02)
Change in Total WBC (when ≤ 2,000)	-	-	4.85 (0.85)
Change in Total WBC (when > 2,000)	-	-	0.03 (-0.06)
Day of illness * Change in persistent vomiting (already occurred)	-	-	- (-0.08)
Day of illness * Change in persistent vomiting (new occur)	-	-	- (-0.33)
Day of illness * Change in % Lymphocyte	-	-	- (0.03)
Day of illness * Change in Platelet count (when ≤ 150,000)	-	-	-0.04 (-0.03)
Day of illness * Change in Platelet count (when > 150,000)	-	-	-0.02 (-0.02)
Day of illness * Change in Total white blood cell count (when ≤ 2,000)	-	-	-0.72 (-)
Day of illness * Change in Total white blood cell count (when > 2,000)	-	-	-0.03 (-)

Legend for Supplementary Table 12:

Estimates of the regression coefficients for models developed using imputed data are presented in parentheses.

* Interaction between variables

- Regression coefficient not relevant to that model or not selected in the development process

Supplementary Figure 1: Apparent performance of the three models, evaluated at baseline (study day 1) and up to 3 days after study enrolment in different subsets of the study population in the imputed dataset (n = 2206).

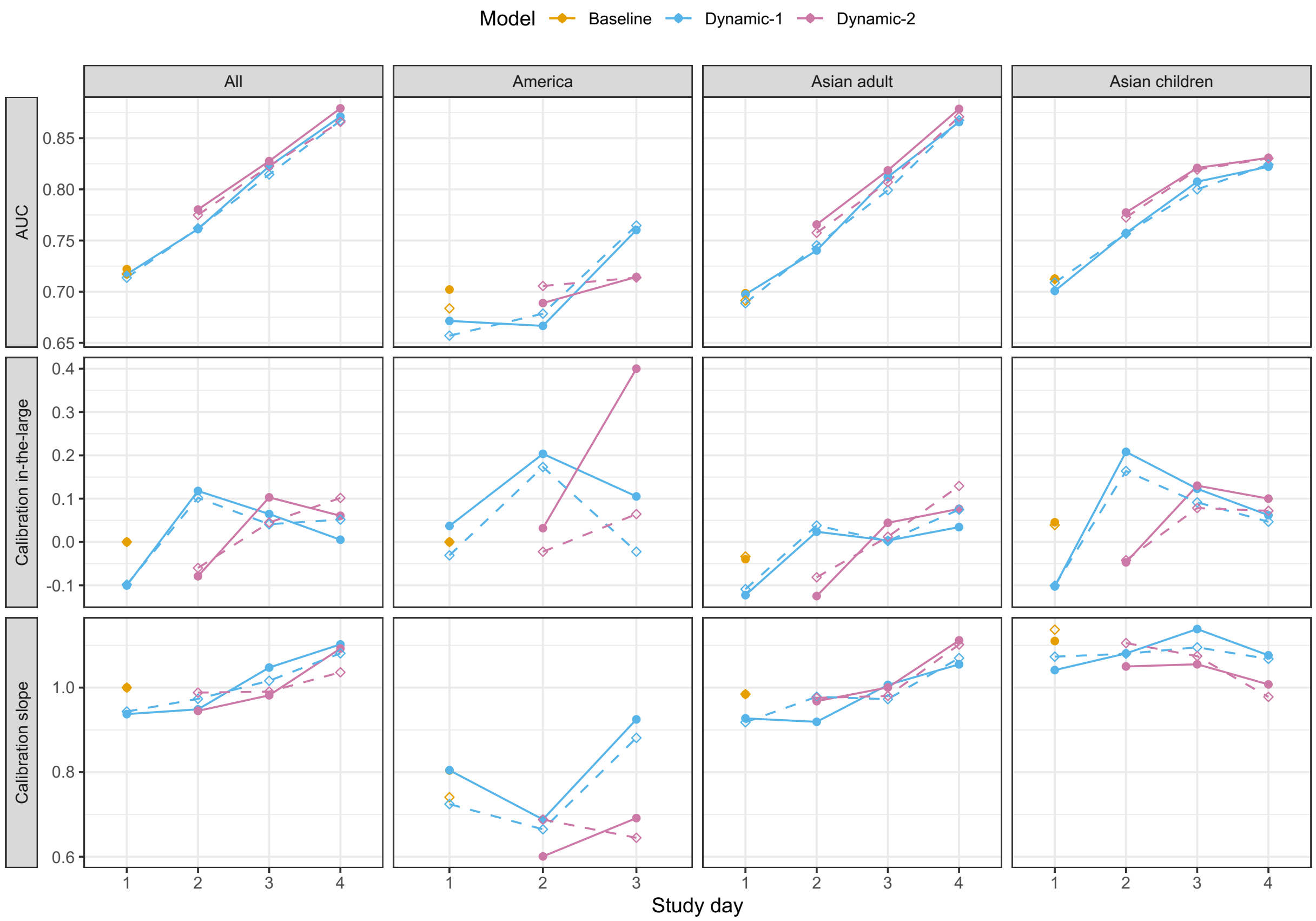

Legend for Supplementary Figure 1:

Symbols represent apparent Area Under the ROC curve (AUC), calibration in-the-large, and calibration slope of the Baseline (orange), Dynamic-1 (blue), Dynamic-2 (purple) models on each study day. Circle symbols and continuous lines refer to models developed using complete case data. Diamond symbols and dashed lines refer to models developed using imputed data.

Supplementary Table 13: Characteristics, linear predictors and membership c-statistics for each study site (n = 2206).

									Linear predictor		Membership c-statistics		
Index	Site	City	Country	Continent	Enrolment period	Age (y)	N	N event (%)	Mean	SD	Full	Age	Sex
1	2	Ho Chi Minh City	Vietnam	Asia	10/2011-10/2015	5-15	367	41 (11)	-2.19	0.83	0.99	0.80	0.53
2	3	Ho Chi Minh City	Vietnam	Asia	10/2011-10/2015	5-63	437	78 (18)	-1.82	0.69	0.97	0.74	0.54
3	7	Ho Chi Minh City	Vietnam	Asia	10/2011-10/2015	5-60	405	60 (15)	-1.83	0.76	0.98	0.68	0.50
4	20	Hanoi	Vietnam	Asia	07/2013-12/2014	8-56	77	10 (13)	-2.15	0.85	0.96	0.78	0.60
5	31	Siem Reap	Cambodia	Asia	03/2012-10/2015	5-16	199	25 (13)	-2.31	0.81	0.99	0.80	0.52
6	34	Dhaka	Bangladesh	Asia	08/2014-10/2015	9-54	57	1 (2)	-2.49	0.73	1.00	0.74	0.50
7	41	Yogyakarta	Indonesia	Asia	06/2012-09/2014	12-14	3	1 (33)	-1.22	0.63	1.00	0.94	0.54
8	42	Yogyakarta	Indonesia	Asia	10/2012-10/2015	11-22	9	0 (0)	-2.16	0.60	1.00	0.84	0.65
9	43	Yogyakarta	Indonesia	Asia	04/2013-06/2015	7-54	9	5 (56)	-1.90	0.71	0.99	0.54	0.54
10	44	Yogyakarta	Indonesia	Asia	01/2013-02/2013	21-25	4	0 (0)	-1.41	0.91	1.00	0.95	0.71
11	46	Yogyakarta	Indonesia	Asia	10/2014-06/2015	10-23	6	2 (33)	-2.26	0.40	1.00	0.70	0.63
12	47	Yogyakarta	Indonesia	Asia	04/2014-05/2015	19-21	7	0 (0)	-2.05	1.01	1.00	0.89	0.58
13	51	Kuala Lumpur	Malaysia	Asia	04/2012-09/2015	12-73	56	11 (20)	-2.22	0.97	0.96	0.78	0.52
14	52	Kuala Lumpur	Malaysia	Asia	07/2013-06/2015	11-60	73	12 (16)	-2.10	0.76	0.98	0.81	0.51
15	53	Kuala Lumpur	Malaysia	Asia	03/2013-08/2014	18-49	6	1 (17)	-1.78	0.58	1.00	0.83	0.54
16	54	Kuala Lumpur	Malaysia	Asia	02/2013-10/2015	13-65	46	3 (7)	-2.19	0.70	0.98	0.76	0.58
17	55	Kuala Lumpur	Malaysia	Asia	09/2013-05/2014	11-58	10	1 (10)	-2.19	0.66	0.99	0.75	0.61
18	61	Fortaleza	Brazil	America	06/2012-08/2015	6-57	28	3 (11)	-2.59	0.79	0.98	0.71	0.56
19	62	Recife	Brazil	America	06/2015	45	1	0 (0)	-2.55	-	1.00	0.97	0.71
20	63	Rio	Brazil	America	04/2015	34	1	0 (0)	-3.03	-	1.00	0.91	0.79
21	64	Rio	Brazil	America	04/2015-05/2015	20-57	14	3 (21)	-2.16	0.70	0.99	0.90	0.61
22	81	San Salvador	El Salvador	America	07/2012-11/2015	5-16	290	14 (5)	-2.88	0.89	0.99	0.83	0.50
23	91	Valencia	Venezuela	America	10/2013-11/2015	6-33	21	3 (14)	-2.14	0.58	0.99	0.66	0.59
24	92	Valencia	Venezuela	America	09/2013-10/2015	8-11	4	0 (0)	-2.32	1.17	1.00	0.85	0.54

Legend for Supplementary Table 13:

SD = standard deviation, N = sample size, N event = number of moderate/severe outcome

Linear predictors for each patient are derived from the full baseline prediction model, without adjustment for continent. Mean and standard deviation of the linear predictors for each site are reported. Mean and standard deviation of the linear predictors in Asia and Latin America are -2.03 (0.80) and -2.78 (0.89), respectively.

Membership c-statistics reflect the discriminative ability to separate a specific study site from all other sites (comparing predicted probabilities for patients from one site with predicted probabilities of patients not from that site; predicted probabilities derived from a membership model [multinomial logistic regression with site as outcome and candidate predictors as covariates]). A membership c-statistic of 1 means that patients from that site can be perfectly differentiated from patients from all other sites combined based on information captured in the membership model. A membership c-statistic of 0.5 means the ability to differentiate patients from that site from patients from other sites based on information captured in the membership model is equivalent to a random guess. Reported here are membership c-statistics when the membership model (multinomial logistic regression) contains all baseline candidate predictors (full), or only age (age), or only sex (sex) as covariates.

Supplementary Table 14: Prediction performance a) separately for each site with at least 10 events of moderate/severe dengue, and b) grouped by continent, based on a baseline prediction model that was developed on all other sites or the other continent.

	Event/N (%)	AUC	Calibration in-the-large	Calibration slope
By site				
Ho Chi Minh City-2	41/367 (11)	0.69 (0.61,0.78)	-0.34 (-0.69,-0.02)	1.33 (0.73,1.97)
Ho Chi Minh City -3	78/437 (18)	0.63 (0.56,0.70)	0.31 (0.05,0.55)	1.02 (0.49,1.59)
Ho Chi Minh City -7	60/405 (15)	0.60 (0.53,0.68)	0.06 (-0.23,0.33)	0.60 (0.16,1.06)
Hanoi-20	10/77 (13)	0.67 (0.49,0.86)	-0.11 (-0.84,0.52)	1.53 (0.12,3.32)
Siem Reap-31	25/199 (13)	0.60 (0.47,0.73)	0.13 (-0.33,0.54)	0.34 (-0.18,0.90)
Kuala Lumpur-51	11/56 (20)	0.79 (0.64,0.95)	0.54 (-0.19,1.19)	2.11 (0.78,3.92)
Kuala Lumpur-52	12/73 (16)	0.63 (0.47,0.80)	0.24 (-0.44,0.84)	0.69 (-0.32,1.86)
San Salvador-81	14/290 (5)	0.59 (0.44,0.75)	-0.54 (-1.13,-0.03)	0.47 (-0.26,1.28)
By continent				
Asia		0.57 (0.53,0.60)	0.02 (-0.12,0.16)	0.27 (0.11,0.42)
Latin America		0.62 (0.51,0.74)	-0.33 (-0.79,0.07)	0.63 (0.03,1.29)

Legend for Supplementary Table 14:

Estimates and 95% confidence intevals of performance criteria (Area Under the ROC Curve (AUC), calibration in-the-large, calibration slope) were calculated for each site by validating on that site the model developed without that site. Only sites with at least 10 events of moderate/severe dengue were selected for this procedure. The model is the simplified version of the reduced baseline prediction model with stepwise variable selection using the Akaike Information Criterion (only including age, persistent vomiting, pulse pressure, % lymphocyte, platelet count and white blood cells; all modelled as linear terms, described in Supplementary Notes 3).

Supplementary Figure 2: Optimism-corrected AUCs of additional exploratory models applied on each of the first 3 study days.

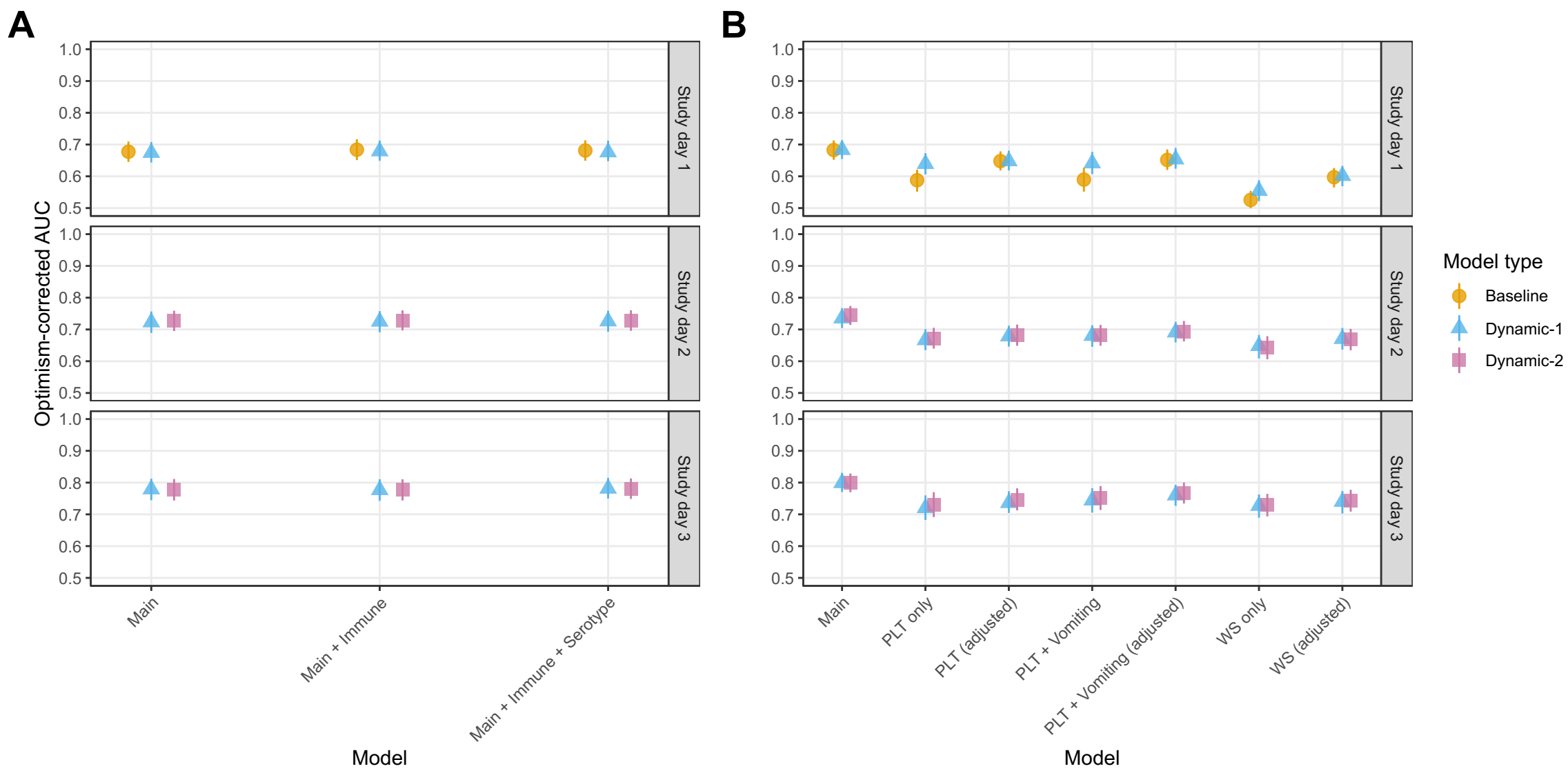

Legend for Supplementary Figure 2:

The coloured symbols represent median optimism-corrected Areas Under the ROC Curves (AUCs), and the vertical lines represent the 2.5th and 97.5th percentiles, based on the 1000 bootstrap values that compute optimism. Where necessary the values presented have been truncated for ease of visualisation: shown are AUCs between 0.5 and 1.

Colours represent model types (Baseline in orange, Dynamic-1 in blue, or Dynamic-2 in purple)

Panel A: Models adding immune status and serotype. "Main" refers to the original models, "Main + Immune" refers to the original models with immune status added, "Main + Immune + Serotype" refers to the original models with both immune status and serotype added. All models were fitted and evaluated only on patients in the primary analysis population with known immune status (probable primary or probable secondary immune status classifications – i.e. those with inconclusive immune status were excluded) and serotype (n = 1586).

Panel B: Models with platelet count and/or WHO warning signs (persistent vomiting, significant abdominal pain/tenderness, lethargy/restlessness, mucosal bleeding, platelet count ≤50K/mm3). "Main" refers to the original models, "PLT only" refers to the orginal models but includes only platelet count on the day of assessment, "PLT (adjusted)" refers to "PLT only" with illness day at enrolment and the interaction between age and continent added, "PLT + Vomiting" refers to the original models but includes only platelet count and persistent vomiting on the day of assessment, "PLT + Vomiting (adjusted)" refers to "PLT + Vomiting" with illness day at enrolment and the interaction between age and continent added, "WS only" refers to the original models but includes only WHO warning signs on the day of assessment, "WS (adjusted)" refers to "WS only" with illness day at enrolment and the interaction between age and continent added. All models were fitted and evaluated only on all patients in the primary analysis population (n = 2130).

Supplementary Figure 3: Study flowchart to describe sample sizes for analyses of suspected dengue (results presented in Supplementary Fig. 4 and Supplementary Tables 15-18).

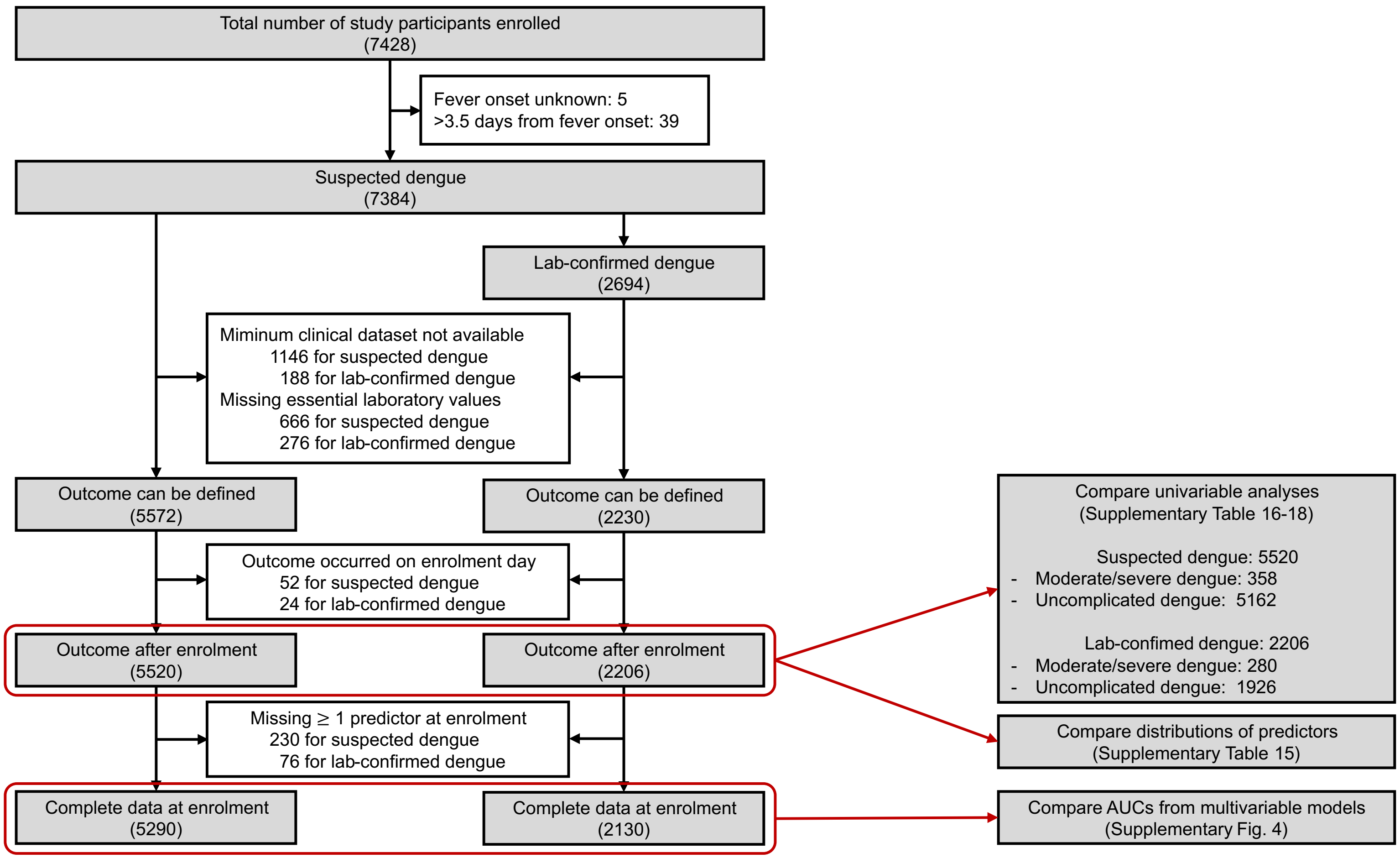

Supplementary Figure 4: Optimism-corrected AUCs of models applied to the confirmed dengue (n = 2130) and suspected dengue populations (n = 5290) on each of the first 3 study days.

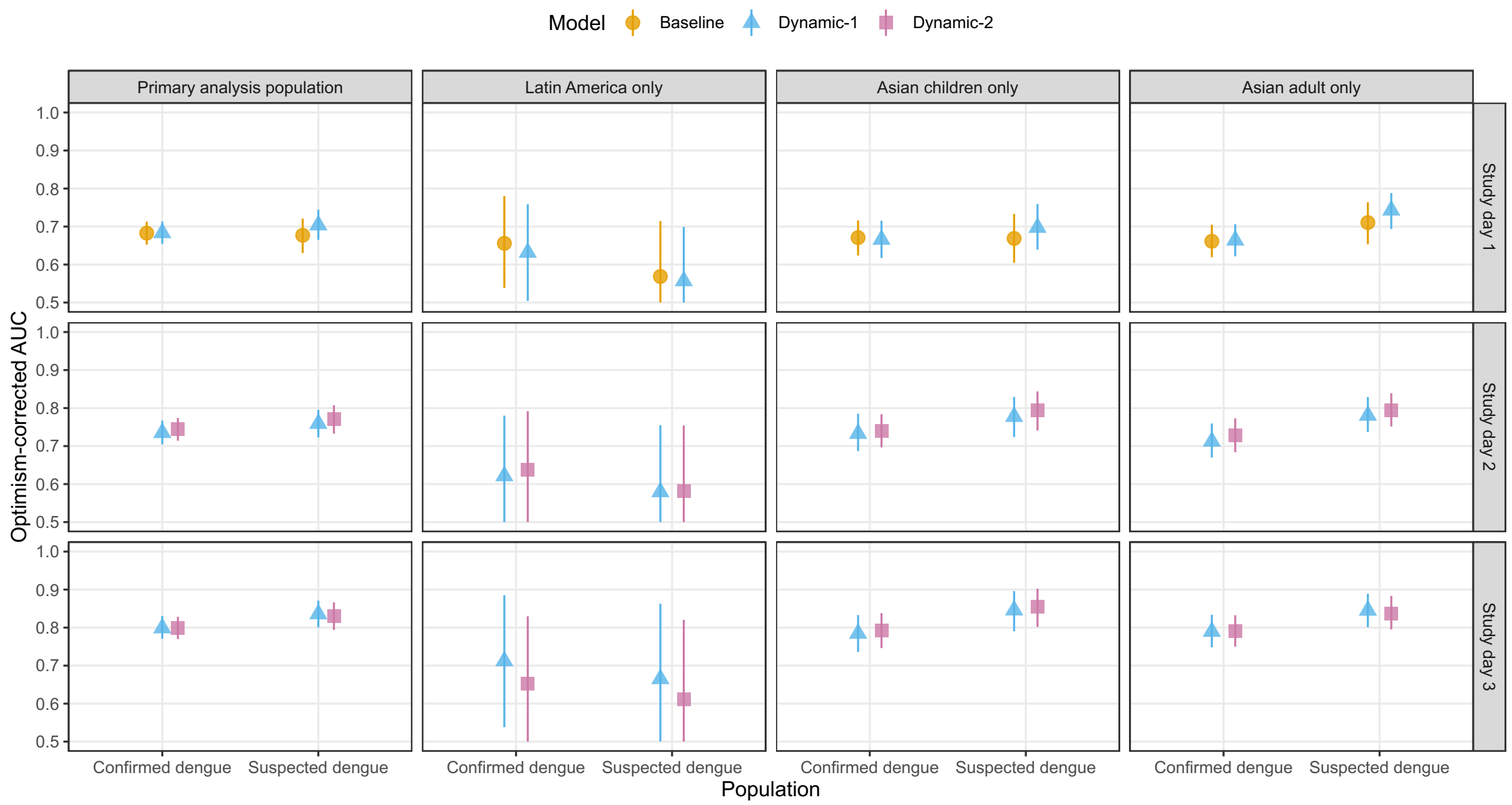

Legend for Supplementary Figure 4:

The coloured symbols represent median optimism-corrected Area Under the ROC Curves (AUCs), and the vertical lines represent the 2.5th and 97.5th percentiles, based on the 1000 bootstrap values that compute optimism. Where necessary the values presented have been truncated for ease of visualisation: shown are AUCs between 0.5 and 1.

Different colours represent the different models (Baseline in orange, Dynamic-1 in blue, and Dynamic-2 in purple), while the populations represent the population that the models were applied to (confirmed dengue or suspected dengue). Of note, the suspected dengue population includes the confirmed dengue population.

Supplementary Table 15: Distribution of candidate predictors at enrolment in the lab-confirmed dengue (n = 2206) and suspected-dengue populations (n = 5520), separately for Latin America and Asia.

Candidate predictor	Lab-confirmed dengue (N = 2206)				Suspected dengue (N = 5520)			
	Asia (N = 1832)		Latin America (N = 374)		Asia (N = 4350)		Latin America (N = 1170)	
	n	Summary	n	Summary	n	Summary	n	Summary
Age (years)	1832	17 (10, 26)	374	9 (7, 12)	4350	16 (9, 26)	1170	11 (7, 24)
Female	1832	770 (42)	374	157 (42)	4350	1827 (42)	1170	553 (47)
Past medical history	1817	268 (15)	374	91 (24)	4326	726 (17)	1170	391 (33)
Chills	1829	1509 (83)	374	302 (81)	4345	3280 (75)	1170	904 (77)
Lymphadenopathy	1832	163 (9)	374	41 (11)	4350	548 (13)	1168	207 (18)
Lethargy/restlessness	1829	97 (5)	374	2 (1)	4339	201 (5)	1165	21 (2)
Cough	1832	498 (27)	374	51 (14)	4349	1897 (44)	1167	266 (23)
Rhinitis	1831	175 (10)	374	23 (6)	4347	776 (18)	1168	111 (10)
Conjunctival injection	1832	928 (51)	374	68 (18)	4350	1624 (37)	1170	280 (24)
Dizzyness	1830	879 (48)	372	154 (41)	4341	1727 (40)	1162	417 (36)
Headache	1832	1628 (89)	374	339 (91)	4348	3693 (85)	1169	1029 (88)
Retro-orbital pain	1829	687 (38)	374	168 (45)	4345	1288 (30)	1169	498 (43)
Joint/muscle pain	1831	1283 (70)	374	318 (85)	4345	2679 (62)	1170	1014 (87)
Persistent vomiting	1831	614 (34)	374	178 (48)	4347	1341 (31)	1167	438 (38)
Diarrhoea	1832	252 (14)	374	52 (14)	4349	515 (12)	1170	161 (14)
Significant abdominal pain/tenderness	1828	86 (5)	371	73 (20)	4342	203 (5)	1156	239 (21)
Skin rash	1823	113 (6)	372	190 (51)	4332	219 (5)	1160	528 (46)
Skin flush	1832	704 (38)	373	259 (69)	4350	1130 (26)	1162	666 (57)
Bleeding	1832		374		4350		1170	
None		1570 (86)		322 (86)		3923 (90)		1041 (89)
Skin only		185 (10)		29 (8)		285 (7)		68 (6)
Mucosal bleeding		77 (4)		23 (6)		142 (3)		61 (5)
Temperature (Celsius degree)	1832	38 (38, 39)	373	38 (38, 39)	4350	38 (38, 39)	1168	38 (37, 38)
Respiratory rate (breaths/min)	1830	22 (20, 24)	372	20 (20, 22)	4346	22 (20, 24)	1160	20 (18, 22)
Pulse (beats/min)	1832	98 (88, 106)	372	100 (90, 112)	4349	98 (88, 108)	1165	98 (84, 110)
Systolic blood pressure (mmHg)	1829	110 (100, 113)	371	100 (90, 100)	4342	110 (100, 117)	1158	100 (90, 110)
Pulse pressure (mmHg)	1829	40 (39, 50)	371	40 (30, 40)	4342	40 (37, 50)	1157	40 (30, 40)
Platelet count (1,000 cells/mm3)	1827	158 (120, 198)	369	194 (158, 239)	4335	194 (148, 251)	1144	219 (175, 272)
Total WBC (1,000 cells/mm3)	1826	4 (3, 6)	369	4 (3, 6)	4334	6 (4, 9)	1143	6 (4, 8)
% Lymphocyte (%)	1817	20 (13, 28)	369	29 (21, 40)	4315	20 (13, 28)	1145	26 (17, 37)
Illness day at enrolment	1832		374		4350		1170	
1		299 (16)		58 (16)		730 (17)		210 (18)
2		765 (42)		130 (35)		2038 (47)		484 (41)
3		768 (42)		186 (50)		1582 (36)		476 (41)
Secondary infection	1600	1225 (77)	224	102 (46)	1600	1225 (77)	224	102 (46)
Serotype	1701		261		1701		261	
DENV-1		823 (48)		48 (18)		823 (48)		48 (18)
DENV-2		284 (17)		15 (6)		284 (17)		15 (6)
DENV-3		111 (7)		196 (75)		111 (7)		196 (75)
DENV-4		483 (28)		2 (1)		483 (28)		2 (1)

Legend for Supplementary Table 15:

N is the number of all patients; n is the number of patients with non-missing values.

Summary is the absolute count (%) for categorical variable(s) and median (1st and 3rd quartiles) for continuous variable(s).

WBC: white blood cell count.

Supplementary Table 16. Univariable relationships between each candidate predictor and outcome in the lab-confirmed dengue and suspected-dengue populations in Latin America.

Candidate predictor	Moderate/severe dengue (confirmed)	Uncomplicated dengue (confirmed)	Uncomplicated dengue (suspected)	Mod/severe vs. uncomplicated (confirmed)	Mod/severe vs. uncomplicated (suspected)
	Summary	Summary	Summary	OR (95% CI)	OR (95% CI)
n	24	350	1121		
Age (years)	11 (8, 24)	9 (7, 11)	11 (7, 24)	1.25 (1.07 – 1.47)	1.01 (0.92 – 1.12)
Female	9 (38%)	148 (42%)	530 (47%)	0.82 (0.35 – 1.92)	0.99 (0.56 – 1.75)
Past medical history	9 (38%)	82 (23%)	373 (33%)	1.96 (0.83 – 4.65)	1.16 (0.64 – 2.11)
Chills	17 (71%)	285 (81%)	866 (77%)	0.55 (0.22 – 1.39)	1.02 (0.51 – 2.02)
Lymphadenopathy	3 (12%)	38 (11%)	196 (18%)	1.17 (0.33 – 4.12)	1.36 (0.68 – 2.71)
Lethargy/restlessness	0 (0%)	2 (1%)	21 (2%)	-	-
Cough	3 (12%)	48 (14%)	257 (23%)	0.90 (0.26 – 3.13)	0.75 (0.36 – 1.57)
Rhinitis	1 (4%)	22 (6%)	107 (10%)	0.65 (0.08 – 5.02)	0.84 (0.30 – 2.38)
Conjunctival injection	5 (21%)	63 (18%)	270 (24%)	1.20 (0.43 – 3.33)	0.81 (0.40 – 1.64)
Dizziness	7 (29%)	147 (42%)	398 (36%)	0.56 (0.23 – 1.29)	1.14 (0.63 – 2.05)
Headache	21 (88%)	318 (91%)	987 (88%)	0.70 (0.20 – 2.49)	0.81 (0.36 – 1.84)
Retro-orbital pain	14 (58%)	154 (44%)	479 (43%)	1.78 (0.77 – 4.12)	0.85 (0.47 – 1.52)
Joint/muscle pain	20 (83%)	298 (85%)	970 (87%)	0.87 (0.29 – 2.66)	1.37 (0.53 – 3.51)
Persistent vomiting	10 (42%)	168 (48%)	422 (38%)	0.77 (0.33 – 1.79)	0.83 (0.45 – 1.52)
Diarrhoea	3 (12%)	49 (14%)	157 (14%)	0.88 (0.25 – 3.05)	0.55 (0.19 – 1.54)
Significant abdominal pain/tenderness	5 (21%)	68 (20%)	227 (21%)	1.08 (0.39 – 2.99)	1.26 (0.65 – 2.45)
Skin rash	13 (57%)	177 (51%)	503 (45%)	1.26 (0.54 – 2.96)	1.32 (0.74 – 2.35)
Skin flush	12 (52%)	247 (71%)	640 (57%)	0.45 (0.19 – 1.06)	0.88 (0.49 – 1.56)
Bleeding					
None	18 (75%)	304 (87%)	1001 (89%)	1.00	1.00
Skin only	4 (17%)	25 (7%)	62 (6%)	2.70 (0.85 – 8.60)	2.42 (0.99 – 5.93)
Mucosal bleeding	2 (8%)	21 (6%)	58 (5%)	1.61 (0.35 – 7.40)	1.29 (0.39 – 4.31)
Temperature (Celsius degree)	38 (37, 39)	38 (38, 39)	38 (37, 38)	0.89 (0.57 – 1.39)	1.18 (0.88 – 1.59)
Respiratory rate (breaths/min)	20 (20, 22)	20 (20, 22)	20 (18, 22)	0.73 (0.34 – 1.58)	1.53 (1.02 – 2.31)
Pulse (beats/min)	100 (80, 110)	100 (90, 112)	98 (84, 110)	0.90 (0.69 – 1.17)	1.03 (0.86 – 1.23)
Systolic blood pressure (mmHg)	102 (100, 110)	100 (90, 100)	100 (90, 110)	1.13 (0.97 – 1.31)	1.03 (0.95 – 1.12)
Pulse pressure (mmHg)	34 (30, 40)	40 (30, 40)	40 (30, 40)	1.02 (0.78 – 1.34)	1.07 (0.93 – 1.23)
Platelet count (1,000 cells/mm3)	164 (110, 194)	199 (159, 240)	221 (178, 273)	0.89 (0.82 – 0.96)	0.91 (0.86 – 0.95)
Total WBC (1,000 cells/mm3)	4 (3, 5)	4 (3, 6)	6 (4, 8)	0.56 (0.29 – 1.10)	0.65 (0.43 – 0.99)
% Lymphocyte (%)	24 (18, 38)	30 (22, 40)	26 (17, 37)	0.89 (0.75 – 1.05)	0.96 (0.86 – 1.07)
Illness day at enrolment					
1	5 (21%)	53 (15%)	198 (18%)	1.00	1.00
2	7 (29%)	123 (35%)	467 (42%)	0.60 (0.18 – 1.99)	0.60 (0.28 – 1.28)
3	12 (50%)	174 (50%)	456 (41%)	0.73 (0.25 – 2.17)	0.72 (0.35 – 1.51)
Secondary infection* (18 vs 206)	15 (83%)	87 (42%)	87 (42%)	6.84 (1.92 – 24.35)	6.84 (1.92 – 24.35)
Serotype * (13 vs 248)					
DENV-1	5 (38%)	43 (17%)	43 (17%)	1.00	1.00
DENV-2	1 (8%)	14 (6%)	14 (6%)	0.61 (0.07 – 5.71)	0.61 (0.07 – 5.71)
DENV-3	7 (54%)	189 (76%)	189 (76%)	0.32 (0.10 – 1.05)	0.32 (0.10 – 1.05)
DENV-4	0 (0%)	2 (1%)	2 (1%)	-	-

Legend for Supplementary Table 16:

WBC: white blood cell count; OR: Odds ratio, CI: confidence interval; *: Secondary infection and serotype data were only available in patients with confirmed dengue (numbers in parentheses represent number of non-missing data in confirmed moderate/severe dengue and confirmed uncomplicated dengue).

n is the number of all patients; summary is the absolute count (%) for categorical variable(s) and median (1st and 3rd quartiles) for continuous variable(s).

Supplementary Table 17: Univariable relationships between each candidate predictor and outcome in the lab-confirmed dengue and suspected-dengue populations in Asian children.

Candidate predictor	Moderate/severe dengue (confirmed)	Uncomplicated dengue (confirmed)	Uncomplicated dengue (suspected)	Mod/severe vs. uncomplicated (confirmed)	Mod/severe vs. uncomplicated (suspected)
	Summary	Summary	Summary	OR (95% CI)	OR (95% CI)
n	107	712	1927		
Age (years)	10 (8, 12)	10 (7, 12)	9 (7, 11)	1.26 (0.86 – 1.85)	1.79 (1.30 – 2.46)
Female	43 (40%)	323 (45%)	842 (44)	0.81 (0.54 – 1.22)	0.87 (0.61 – 1.24)
Past medical history	9 (8%)	79 (11%)	242 (13%)	0.72 (0.35 – 1.48)	0.68 (0.37 – 1.24)
Chills	79 (74%)	522 (74%)	1265 (66%)	1.01 (0.64 – 1.60)	1.53 (1.03 – 2.28)
Lymphadenopathy	18 (17%)	115 (16%)	458 (24%)	1.05 (0.61 – 1.81)	0.77 (0.50 – 1.20)
Lethargy/restlessness	8 (7%)	58 (8%)	113 (6%)	0.91 (0.42 – 1.96)	1.43 (0.75 – 2.73)
Cough	33 (31%)	192 (27%)	849 (44%)	1.21 (0.78 – 1.88)	0.64 (0.44 – 0.93)
Rhinitis	13 (12%)	64 (9%)	320 (17%)	1.40 (0.74 – 2.64)	0.73 (0.43 – 1.23)
Conjunctival injection	50 (47%)	257 (36%)	468 (24%)	1.55 (1.03 – 2.34)	2.31 (1.61 – 3.30)
Dizziness	49 (46%)	250 (35%)	524 (27%)	1.55 (1.03 – 2.34)	1.97 (1.38 – 2.82)
Headache	93 (87%)	576 (81%)	1456 (76%)	1.57 (0.87 – 2.84)	2.39 (1.40 – 4.06)
Retro-orbital pain	30 (28%)	170 (24%)	346 (18%)	1.26 (0.80 – 1.98)	1.63 (1.09 – 2.44)
Joint/muscle pain	62 (58%)	313 (44%)	700 (36%)	1.75 (1.16 – 2.64)	2.16 (1.52 – 3.07)
Persistent vomiting	62 (58%)	335 (47%)	816 (42%)	1.55 (1.03 – 2.34)	1.84 (1.29 – 2.62)
Diarrhoea	11 (10%)	59 (8%)	135 (7%)	1.27 (0.64 – 2.50)	2.06 (1.22 – 3.49)
Significant abdominal pain/tenderness	12 (11%)	36 (5%)	89 (5%)	2.36 (1.19 – 4.70)	2.62 (1.47 – 4.67)
Skin rash	6 (6%)	58 (8%)	117 (6%)	0.67 (0.28 – 1.59)	1.11 (0.55 – 2.24)
Skin flush	46 (43%)	224 (31%)	378 (20%)	1.64 (1.09 – 2.49)	2.94 (2.05 – 4.22)
Bleeding					
None	92 (86%)	564 (79%)	1675 (87%)	1.00	1.00
Skin only	10 (9%)	123 (17%)	197 (10%)	0.50 (0.25 – 0.98)	0.79 (0.42 – 1.50)
Mucosal bleeding	5 (5%)	25 (4%)	55 (3%)	1.23 (0.46 – 3.28)	1.29 (0.51 – 3.28)
Temperature (Celsius degree)	39 (38, 39)	38 (38, 39)	38 (38, 39)	1.37 (1.07 – 1.74)	1.48 (1.20 – 1.82)
Respiratory rate (breaths/min)	24 (22, 26)	24 (22, 26)	24 (22, 26)	1.01 (0.77 – 1.34)	0.98 (0.79 – 1.23)
Pulse (beats/min)	100 (90, 114)	100 (90, 110)	100 (90, 112)	1.08 (0.94 – 1.25)	1.09 (0.97 – 1.23)
Systolic blood pressure (mmHg)	105 (100, 110)	100 (100, 110)	100 (100, 110)	1.05 (0.95 – 1.15)	1.12 (1.02 – 1.22)
Pulse pressure (mmHg)	40 (40, 50)	40 (40, 48)	40 (36, 45)	1.05 (0.94 – 1.18)	1.11 (1.01 – 1.22)
Platelet count (1,000 cells/mm3)	168 (114, 214)	177 (136, 223)	222 (172, 275)	0.96 (0.92 – 0.99)	0.92 (0.90 – 0.95)
Total WBC (1,000 cells/mm3)	5 (3, 7)	4 (3, 6)	6 (4, 10)	1.12 (0.84 – 1.50)	0.57 (0.46 – 0.71)
% Lymphocyte (%)	19 (10, 27)	23 (14, 33)	21 (14, 30)	0.84 (0.77 – 0.92)	0.89 (0.82 – 0.96)
Illness day at enrolment					
1	23 (21%)	136 (19%)	375 (19%)	1.00	1.00
2	47 (44%)	298 (42%)	946 (49%)	0.93 (0.54 – 1.60)	0.78 (0.49 – 1.24)
3	37 (35%)	278 (39%)	606 (31%)	0.79 (0.45 – 1.38)	1.02 (0.63 – 1.65)
Secondary infection* (100 vs 617)	88 (88%)	462 (75%)	462 (75%)	2.46 (1.31 – 4.62)	2.46 (1.31 – 4.62)
Serotype * (103 vs 677)					
DENV-1	52 (50%)	390 (58%)	390 (58%)	1.00	1.00
DENV-2	16 (16%)	76 (11%)	76 (11%)	1.58 (0.86 – 2.91)	1.58 (0.86 – 2.91)
DENV-3	9 (9%)	38 (6%)	38 (6%)	1.78 (0.81 – 3.88)	1.78 (0.81 – 3.88)
DENV-4	26 (25%)	173 (26%)	173 (26%)	1.13 (0.68 – 1.87)	1.13 (0.68 – 1.87)

Legend for Supplementary Table 17:

WBC: white blood cell count; OR: Odds ratio, CI: confidence interval; *: Secondary infection and serotype data were only available in patients with confirmed dengue (numbers in parentheses represent number of non-missing data in confirmed moderate/severe dengue and confirmed uncomplicated dengue).

n is the number of all patients; summary is the absolute count (%) for categorical variable(s) and median (1st and 3rd quartiles) for continuous variable(s).

Supplementary Table 18: Univariable relationships between each candidate predictor and outcome in the lab-confirmed dengue and suspected-dengue populations in Asian adults.

Candidate predictor	Moderate/severe dengue (confirmed)	Uncomplicated dengue (confirmed)	Uncomplicated dengue (suspected)	Mod/severe vs. uncomplicated (confirmed)	Mod/severe vs. uncomplicated (suspected)
n	149	864	2114		
Age (years)	22 (18, 30)	25 (20, 31)	26 (20, 34)	0.91 (0.82 – 1.01)	0.84 (0.77 – 0.92)
Female	62 (42%)	342 (40%)	859 (41%)	1.09 (0.76 – 1.55)	1.02 (0.75 – 1.40)
Past medical history	23 (15%)	157 (18%)	444 (21%)	0.82 (0.51 – 1.32)	0.72 (0.47 – 1.09)
Chills	135 (91%)	773 (89%)	1760 (83%)	1.14 (0.63 – 2.05)	1.55 (0.96 – 2.51)
Lymphadenopathy	1 (1%)	29 (3%)	63 (3%)	0.19 (0.03 – 1.44)	0.19 (0.03 – 1.36)
Lethargy/restlessness	9 (6%)	22 (3%)	63 (3%)	2.46 (1.11 – 5.45)	2.32 (1.23 – 4.38)
Cough	45 (30%)	228 (26%)	942 (45%)	1.21 (0.82 – 1.77)	0.67 (0.48 – 0.92)
Rhinitis	18 (12%)	80 (9%)	416 (20%)	1.34 (0.78 – 2.32)	0.62 (0.39 – 0.97)
Conjunctival injection	100 (67%)	521 (60%)	991 (47%)	1.34 (0.93 – 1.94)	1.83 (1.33 – 2.51)
Dizziness	96 (64%)	484 (56%)	1035 (49%)	1.42 (0.99 – 2.04)	1.80 (1.31 – 2.48)
Headache	144 (97%)	815 (94%)	1954 (93%)	1.73 (0.68 – 4.42)	1.33 (0.69 – 2.58)
Retro-orbital pain	72 (48%)	415 (48%)	828 (39%)	1.01 (0.71 – 1.43)	1.28 (0.93 – 1.74)
Joint/muscle pain	137 (92%)	771 (89%)	1751 (83%)	1.38 (0.73 – 2.58)	1.51 (0.95 – 2.42)
Persistent vomiting	43 (29%)	174 (20%)	397 (19%)	1.61 (1.09 – 2.38)	1.78 (1.26 – 2.50)
Diarrhoea	26 (17%)	156 (18%)	328 (16%)	0.96 (0.61 – 1.52)	1.31 (0.89 – 1.94)
Significant abdominal pain/tenderness	7 (5%)	31 (4%)	90 (4%)	1.32 (0.57 – 3.06)	1.22 (0.60 – 2.46)
Skin rash	6 (4%)	43 (5%)	85 (4%)	0.80 (0.33 – 1.92)	1.15 (0.55 – 2.42)
Skin flush	67 (45%)	367 (42%)	624 (30%)	1.11 (0.78 – 1.57)	1.67 (1.22 – 2.29)
Bleeding					
None	142 (95%)	772 (89%)	1962 (93%)	1.00	1.00
Skin only	4 (3%)	48 (6%)	73 (3%)	0.45 (0.16 – 1.28)	0.64 (0.23 – 1.77)
Mucosal bleeding	3 (2%)	44 (5%)	79 (4%)	0.37 (0.11, 1.21)	0.44 (0.14 – 1.42)
Temperature (Celsius degree)	39 (38, 39)	38 (38, 39)	38 (38, 39)	1.27 (1.02 – 1.59)	1.59 (1.32 – 1.92)
Respiratory rate (breaths/min)	20 (20, 22)	20 (20, 22)	20 (18, 22)	1.13 (0.83 – 1.53)	1.35 (1.05 – 1.73)
Pulse (beats/min)	100 (88, 104)	97 (88, 102)	94 (85, 100)	1.06 (0.93 – 1.22)	1.14 (1.01 – 1.28)
Systolic blood pressure (mmHg)	110 (100, 120)	110 (100, 120)	110 (100, 120)	0.97 (0.90 – 1.04)	0.91 (0.85 – 0.96)
Pulse pressure (mmHg)	40 (40, 50)	40 (35, 50)	40 (38, 50)	1.05 (0.97 – 1.14)	0.99 (0.92 – 1.07)
Platelet count (1,000 cells/mm3)	132 (98, 170)	147 (113, 182)	180 (138, 227)	0.97 (0.94 – 1.00)	0.90 (0.88 – 0.93)
Total WBC (1,000 cells/mm3)	4 (3, 6)	4 (3, 6)	6 (4, 8)	1.06 (0.79 – 1.43)	0.47 (0.38 – 0.58)
% Lymphocyte (%)	15 (11, 22)	19 (14, 26)	19 (13, 26)	0.81 (0.73 – 0.90)	0.85 (0.78 – 0.93)
Illness day at enrolment					
1	25 (17%)	115 (13%)	297 (14%)	1.00	1.00
2	74 (50%)	346 (40%)	949 (45%)	0.98 (0.60 – 1.62)	0.93 (0.60 – 1.44)
3	50 (34%)	403 (47%)	868 (41%)	0.57 (0.34 – 0.96)	0.71 (0.45 – 1.12)
Secondary infection* (139 vs 744)	114 (82%)	561 (75%)	561 (75%)	1.49 (0.94 – 2.37)	1.49 (0.94 – 2.37)
Serotype * (142 vs 779)					
DENV-1	59 (42%)	322 (41%)	322 (41%)	1.00	1.00
DENV-2	28 (20%)	164 (21%)	164 (21%)	0.93 (0.57 – 1.52)	0.93 (0.57 – 1.52)
DENV-3	12 (8%)	52 (7%)	52 (7%)	1.26 (0.63 – 2.50)	1.26 (0.63 – 2.50)
DENV-4	43 (30%)	241 (31%)	241 (31%)	0.97 (0.64 – 1.49)	0.97 (0.64 – 1.49)

Legend for Supplementary Table 18:

WBC: white blood cell count; OR: Odds ratio, CI: confidence interval; *: Secondary infection and serotype data were only available in patients with confirmed dengue (numbers in parentheses represent number of non-missing data in confirmed moderate/severe dengue and confirmed uncomplicated dengue).

n is the number of all patients; summary is the absolute count (%) for categorical variable(s) and median (1st and 3rd quartiles) for continuous variable(s).

Supplementary Figure 5: A demonstration of the web application for the three models developed (URL: https://lampk.shinyapps.io/IDAMSPRED/)

Proof-of-concept Application for Dynamic Prediction of Moderate/Severe Dengue (IDAMS study)

Demographic information

Continent

(●) Asia ( ) Latin America

Age (year)

5 — 15 — 73

Illness day at enrolment

( ) 1 (●) 2 ( ) 3

Signs - symptoms & Laboratory test

Illness day	2	3	4	5	6	7	8
Current day	0	0	1	1	0	0	0
Chills	1	1	1	1			
Lethargy/Restlessness	0	1	0	1			
Cough	0	0	0	1			
Rhinitis	0	0	0	0			
Conjunctival injection	0	0	0	0			
Dizzy	0	1	1	1			
Headache	1	1	1	1			
Joint/Muscle pain	1	1	1	1			
Vomiting	1	1	1	1			
Diarrhoea	0	0	0	0			
Abdominal pain/tenderness	0	0	0	1			
Bleeding	0	0	0	0			
Body temperature [Celsius degree]	39.4	39.4	39.4	39.4			
Pulse rate [per min]	116	112	130	130			
Pulse pressure [mmHg]	42	26	47	37			
Total WBC count [1000/mm3]	11	6.4	3	4.2			
% Lymphocytes [%]	9.1	12.5	16.7	19			
Platelet count [1000/mm3]	186	144	74	19			

Predicted risk of moderate/severe dengue (on current day)

Baseline	Dynamic 1	Dynamic 2
0.20	0.88	0.87

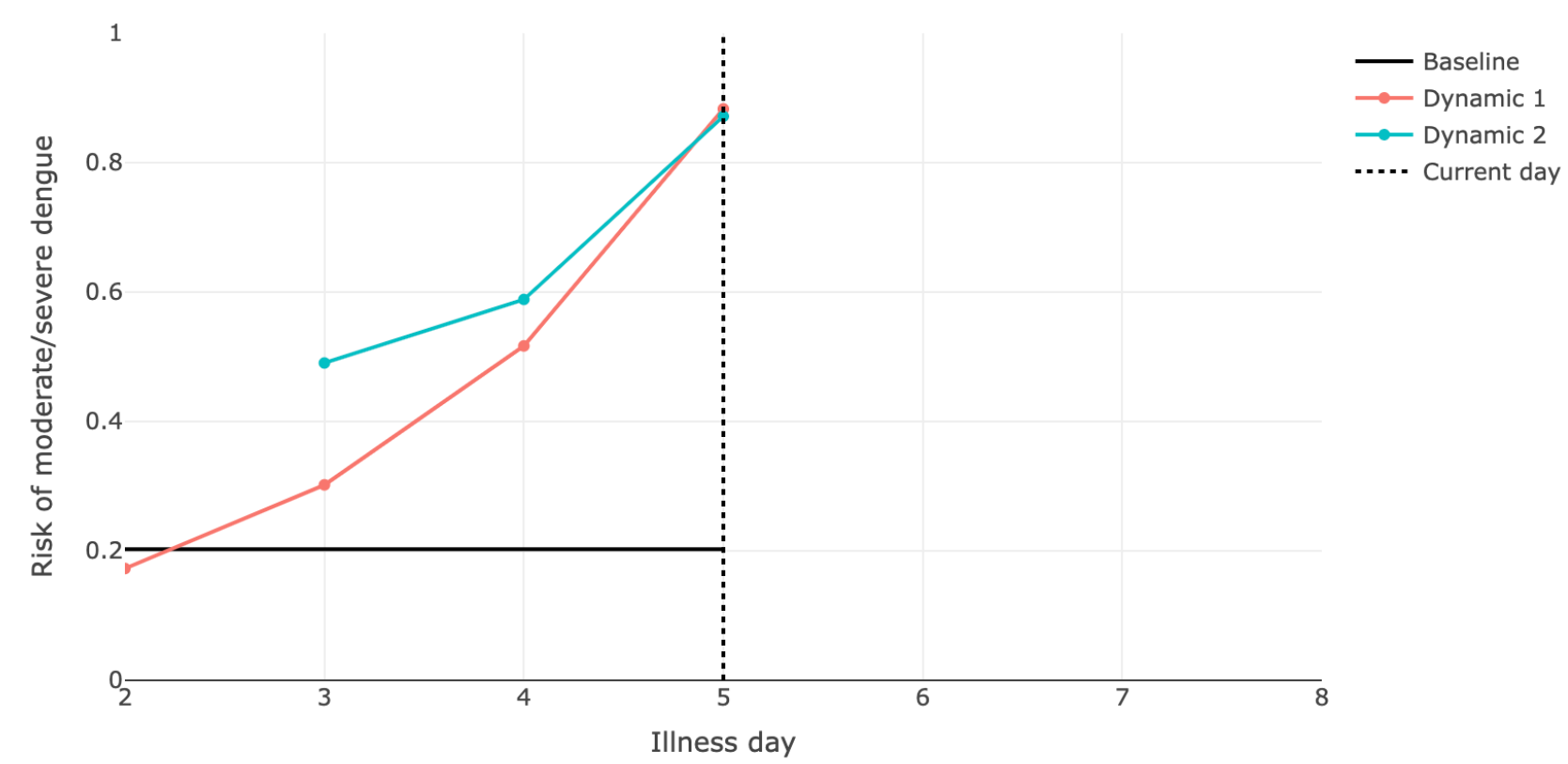

Note

To make prediction:

- Change 'Current day' to 1 on the day that need prediction.
- Change default values of predictors to the true values
- For binary predictors (highlighted in orange): 0 = 'No', 1 = 'Yes'
- For bleeding: 0 = 'No', 1 = 'Skin only bleeding', 2 = 'Mucosal bleeding'

Warning

Warning: This is just a proof-of-concept application for demonstration purpose !!!

Supplementary Figure 6: Number of cases with uncomplicated dengue (neither moderate nor severe) (Panel A) or non-severe dengue (Panel B) hospitalised for each patient pre-emptively admitted prior to development of the relevant outcome, at different risk thresholds.

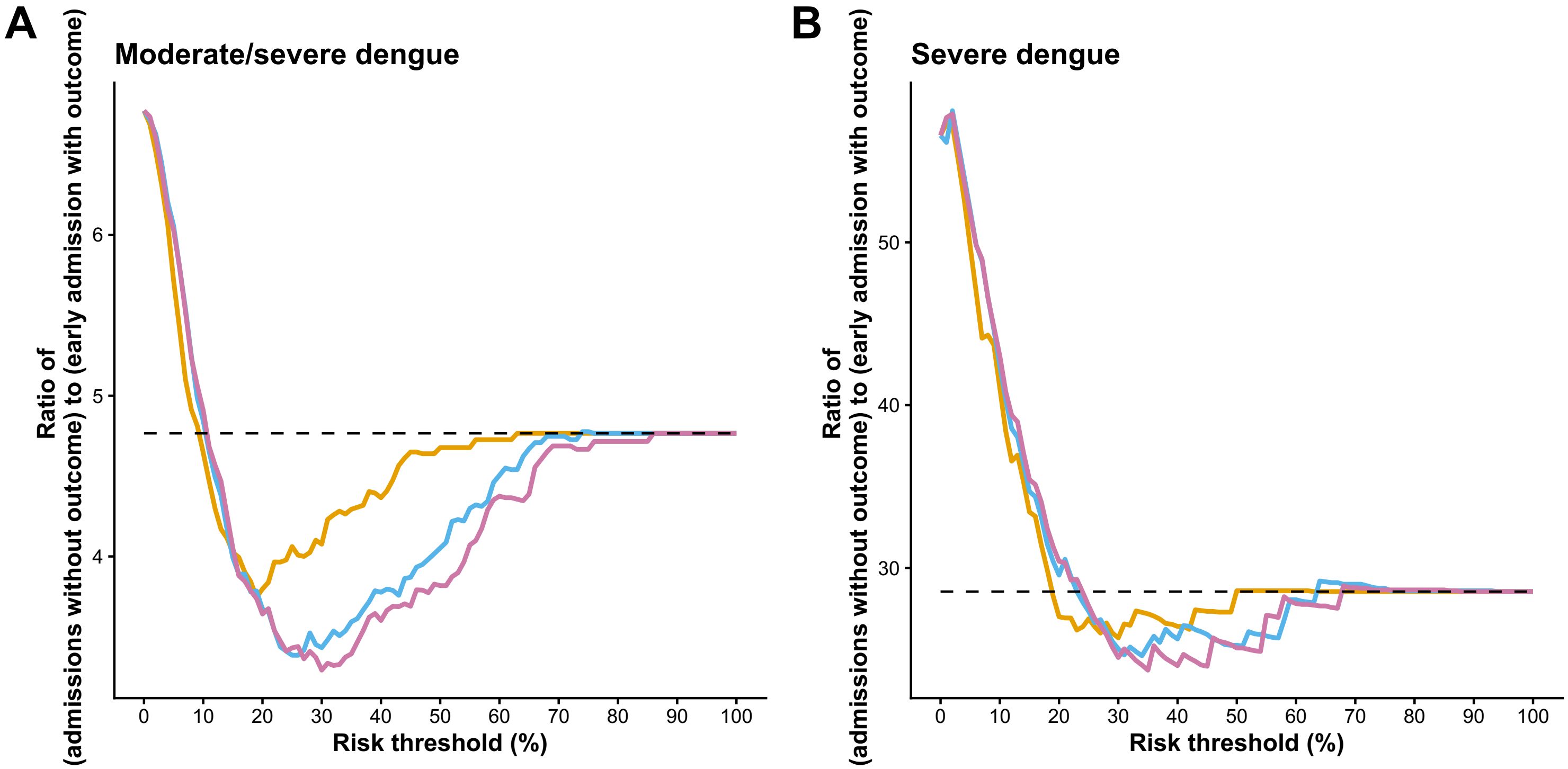

Legend for Supplementary Figure 6:

To evaluate decision rules that combine the clinicians' decisions and our developed models, we calculated the number of patients that did not proceed to the selected clinical outcome (moderate/severe, or severe, respectively), but were hospitalised, for each patient who did proceed to the selected outcome at least one day after admission. The continuous lines in different colours correspond to combinations using the Baseline (orange), Dynamic-1 (blue), or Dynamic-2 models (purple). The dashed horizontal line represents the number of patients without the clinical outcome admitted, for each patient with the outcome detected at least one day in advance, based on the clinicians' decisions only.

Supplementary Figure 7: Distributions of the predicted risks derived from the Dynamic-1 and Dynamic-2 models on the day of hospitalisation, for patients who were hospitalised for medical reasons, stratified by study site (n = 2130).

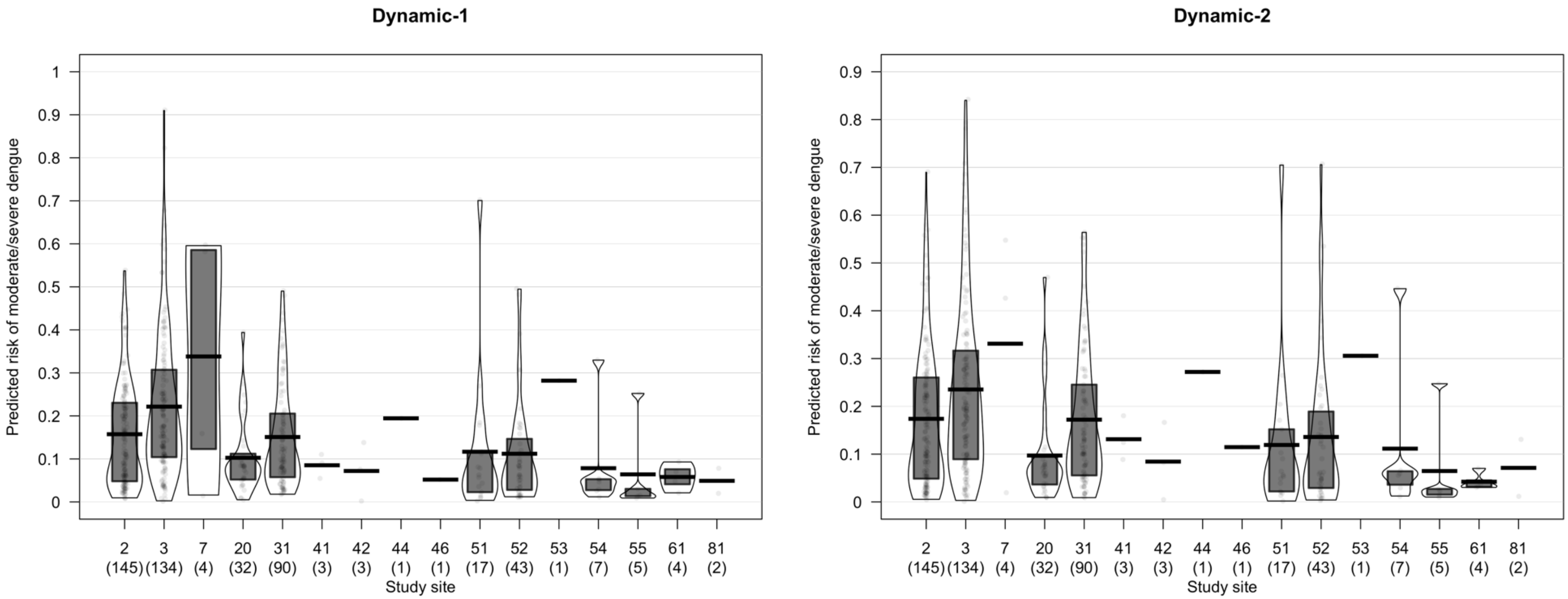

Legend for Supplementary Figure 7:

Study sites are coded numerically. Numbers in parentheses represent the sample size at each site. Individual predicted risks are displayed as points, while their distribution summaries are displayed as horizontal bars (mean), bean-shaped areas (smoothed density), and rectangles (interquartile range).

3. TRIPOD Checklist: Prediction Model Development

Section/Topic	Item	Checklist Item	Page
Title and abstract			
Title	1	Identify the study as developing and/or validating a multivariable prediction model, the target population, and the outcome to be predicted.	Main text p1
Abstract	2	Provide a summary of objectives, study design, setting, participants, sample size, predictors, outcome, statistical analysis, results, and conclusions.	Main text p3
Introduction			
Background and objectives	3a	Explain the medical context (including whether diagnostic or prognostic) and rationale for developing or validating the multivariable prediction model, including references to existing models.	Main text p4-5
	3b	Specify the objectives, including whether the study describes the development or validation of the model or both.	Main text p5
Methods			
Source of data	4a	Describe the study design or source of data (e.g., randomized trial, cohort, or registry data), separately for the development and validation data sets, if applicable.	Main text p6,18
	4b	Specify the key study dates, including start of accrual; end of accrual; and, if applicable, end of follow-up.	Main text p6,18<br>Supplementary Table 13
Participants	5a	Specify key elements of the study setting (e.g., primary care, secondary care, general population) including number and location of centres.	Main text p6<br>Supplementary Methods 1 p5
	5b	Describe eligibility criteria for participants.	Main text p6<br>Supplementary Methods 1 p5
	5c	Give details of treatments received, if relevant.	Main text p6-7<br>Table 1<br>Supplementary Methods 4 p6-7
Outcome	6a	Clearly define the outcome that is predicted by the prediction model, including how and when assessed.	Main text 19<br>Supplementary Methods 6 p8-10<br>Supplementary Table 2
	6b	Report any actions to blind assessment of the outcome to be predicted.	Supplementary Methods 6 p8-9
Predictors	7a	Clearly define all predictors used in developing or validating the multivariable prediction model, including how and when they were measured.	Main text p19<br>Supplementary Methods 7 p10-11<br>Supplementary Table 3
	7b	Report any actions to blind assessment of predictors for the outcome and other predictors.	-
Sample size	8	Explain how the study size was arrived at.	Published protocol: citation 31 in main text
Missing data	9	Describe how missing data were handled (e.g., complete-case analysis, single imputation, multiple imputation) with details of any imputation method.	Main text p24-25<br>Supplementary Notes 1 p14
Statistical analysis methods	10a	Describe how predictors were handled in the analyses.	Main text 21
	10b	Specify type of model, all model-building procedures (including any predictor selection), and method for internal validation.	Main text p20-23<br>Fig. 4
	10d	Specify all measures used to assess model performance and, if relevant, to compare multiple models.	Main text p22-23
Risk groups	11	Provide details on how risk groups were created, if done.	-
Results			

Participants	13a	Describe the flow of participants through the study, including the number of participants with and without the outcome and, if applicable, a summary of the follow-up time. A diagram may be helpful.	Main text p6<br>Fig. 1
	13b	Describe the characteristics of the participants (basic demographics, clinical features, available predictors), including the number of participants with missing data for predictors and outcome.	Main text p6-7<br>Table 1<br>Supplementary Notes 1 p14<br>Supplementary Table 9
Model development	14a	Specify the number of participants and outcome events in each analysis.	Fig. 1<br>Supplementary Fig. 3
	14b	If done, report the unadjusted association between each candidate predictor and outcome.	Table 2
Model specification	15a	Present the full prediction model to allow predictions for individuals (i.e., all regression coefficients, and model intercept or baseline survival at a given time point).	Table 3<br>Supplementary Fig. 5
	15b	Explain how to the use the prediction model.	Table 3
Model performance	16	Report performance measures (with CIs) for the prediction model.	Fig. 6,<br>Supplementary Fig. 1
Discussion			
Limitations	18	Discuss any limitations of the study (such as nonrepresentative sample, few events per predictor, missing data).	Main text p14-15
Interpretation	19b	Give an overall interpretation of the results, considering objectives, limitations, and results from similar studies, and other relevant evidence.	Main text p12-16
Implications	20	Discuss the potential clinical use of the model and implications for future research.	Main text p13,16
Other information			
Supplementary information	21	Provide information about the availability of supplementary resources, such as study protocol, Web calculator, and data sets.	Main text p9,18,26<br>Figure S5
Funding	22	Give the source of funding and the role of the funders for the present study.	Main text p31

SUPPLEMENTARY REFERENCES

1. Hue KDT, Tuan TV, Thi HTN, *et al.* Validation of an internally controlled one-step real-time multiplex RT-PCR assay for the detection and quantitation of dengue virus RNA in plasma. *J Virol Methods* 2011; **177**: 168–73.

2. Steyerberg EW, Nieboer D, Debray TPA, van Houwelingen HC. Assessment of heterogeneity in an individual participant data meta-analysis of prediction models: An overview and illustration. *Stat Med* 2019; **38**: 4290–309.
